# Crystal structures of four thio­glycosides involving carbamimido­thio­ate groups

**DOI:** 10.1107/S2056989024006455

**Published:** 2024-07-09

**Authors:** Mamdouh A. Abu-Zaied, Galal A. Nawwar, Galal H. Elgemeie, Peter G. Jones

**Affiliations:** a Green Chemistry Department, Chemical Industries Research Institute, National Research Centre, Dokki, Giza 12622, Egypt; b Chemistry Department, Faculty of Science, Helwan University, Cairo, Egypt; c Institut für Anorganische und Analytische Chemie, Technische Universität Braunschweig, Hagenring 30, D-38106 Braunschweig, Germany; Universität Greifswald, Germany

**Keywords:** crystal structure, glucose, galactose, carbamimido­thio­ate, hydrogen bonds

## Abstract

The compounds 2′,3′,4′,6′-tetra-*O*-acetyl-β-d-gluco­pyranosyl *N*′-cyano-*N*-phenyl­carbamimido­thio­ate (C_22_H_25_N_3_O_9_S, **5a**), 2′,3′,4′,6′-tetra-*O*-acetyl-β-d-galacto­pyranosyl *N*′-cyano-*N-*phenyl­carbamimido­thio­ate, (C_22_H_25_N_3_O_9_S, **5b**), 2′,3′,4′,6′-tetra-*O*-acetyl-β-d-galacto­pyranosyl *N*′-cyano-*N*-methyl­carbamimido­thio­ate (C_17_H_23_N_3_O_9_S, **5c**), and 2′,3′,4′,6′-tetra-*O*-acetyl-β-d-galacto­pyranosyl *N*′-cyano-*N*-*p*-tolyl­carbamimido­thio­ate (C_23_H_27_N_3_O_9_S, **5d**) all crystallize in *P*2_1_2_1_2_1_ with *Z* = 4. For all four structures, the configuration across the central (formal) C=N(CN) double bond of the carbamimido­thio­ate group is *Z*. The torsion angles C5—O1—C1—S (standard sugar numbering) are all close to 180°, confirming the β position of the substituent. Compound **5b** involves an intra­molecular hydrogen bond N—H⋯O1; in **5c** this contact is the weaker branch of a three-centre inter­action, whereas in **5a** and **5d** the H⋯O distances are much longer and do not represent significant inter­actions. The C—N bond lengths at the central carbon atom of the carbamimido­thio­ate group are almost equal. All C—O—C=O torsion angles of the acetyl groups correspond to a synperiplanar geometry, but otherwise all four mol­ecules display a high degree of conformational flexibility, with many widely differing torsion angles for equivalent groups. In the crystal packing, **5a**, **5c** and **5d** form layer structures involving the classical hydrogen bond N—H⋯N_cyano_ and a variety of ‘weak’ hydrogen bonds C—H⋯O or C—H⋯S. The packing of **5b** is almost featureless and involves a large number of borderline ‘weak’ hydrogen bonds. In an appendix, a potted history of wavelength preferences for structure determination is presented and it is recommended that, even for small organic crystals in non-centrosymmetric space groups, the use of Mo radiation should be considered.

## Abstract 2

The structures of the four thio­glycosides, all *Z*-configured across the C=N(CN) moiety, differ in many important torsion angles. The C—N bond lengths at the central carbon atom of the carbamimido­thio­ate group are almost equal. Three of the four structures form layers by hydrogen bonding.

## 1. Chemical context

Many synthetic nitro­gen heterocycles are utilized in medicinal chemistry (Azzam *et al.*, 2023 ▸; Elboshi *et al.*, 2024 ▸). Several of these have played an important role in the search for potent anti­viral drugs (Santos *et al.*, 2021 ▸). As part of our program aimed at developing new, effective and straightforward procedures for the synthesis of anti­metabolites (Elgemeie *et al.*, 1998*a* ▸,*b* ▸, 2015 ▸, 2022 ▸; Mohamed-Ezzat *et al.*, 2024 ▸), we have described several effective syntheses of folic acid, pyrimidine nucleoside and mercaptopurine analogues. One of these (Elgemeie *et al.*, 2015 ▸) presented the synthesis and structure of a carbamimido­thio­ate, namely methyl *N*′-cyano-*N*-(1,5-dimethyl-3-oxo-2-phenyl-2,3-di­hydro-1*H*-pyrazol-4-yl)carbamimido­thio­ate. Recently, the synthesis of nucleoside anal­ogues and their integration into DNA sequences for the investigation of ligand–DNA and protein–DNA inter­actions has attracted increased attention (Dantsu *et al.*, 2021 ▸). Numerous nucleoside derivatives that involve an alteration or removal of the functional groups of heterocyclic bases have been synthesized (Hammad *et al.*, 2018 ▸; Masoud *et al.*, 2017 ▸). The synthesis of oligo­deoxy­nucleotides with a single functional group at a preselected position, involving various novel thio­glycosides that demonstrate antagonistic activity, is made possible by such analogues (Pérez-Rentero *et al.*, 2012 ▸; Warren *et al.*, 1998 ▸). The use of di­hydro­pyridine thio­glycosides as substrates or inhibitors of glycosyl­ation of proteins was reported (Scala *et al.*, 1997 ▸). These results have made the synthesis of modified and acyclic thio­glycosides relevant in the quest for more potent agents (Elgemeie *et al.*, 2017 ▸; Galmarini *et al.*, 2003 ▸).

**Figure 1:**
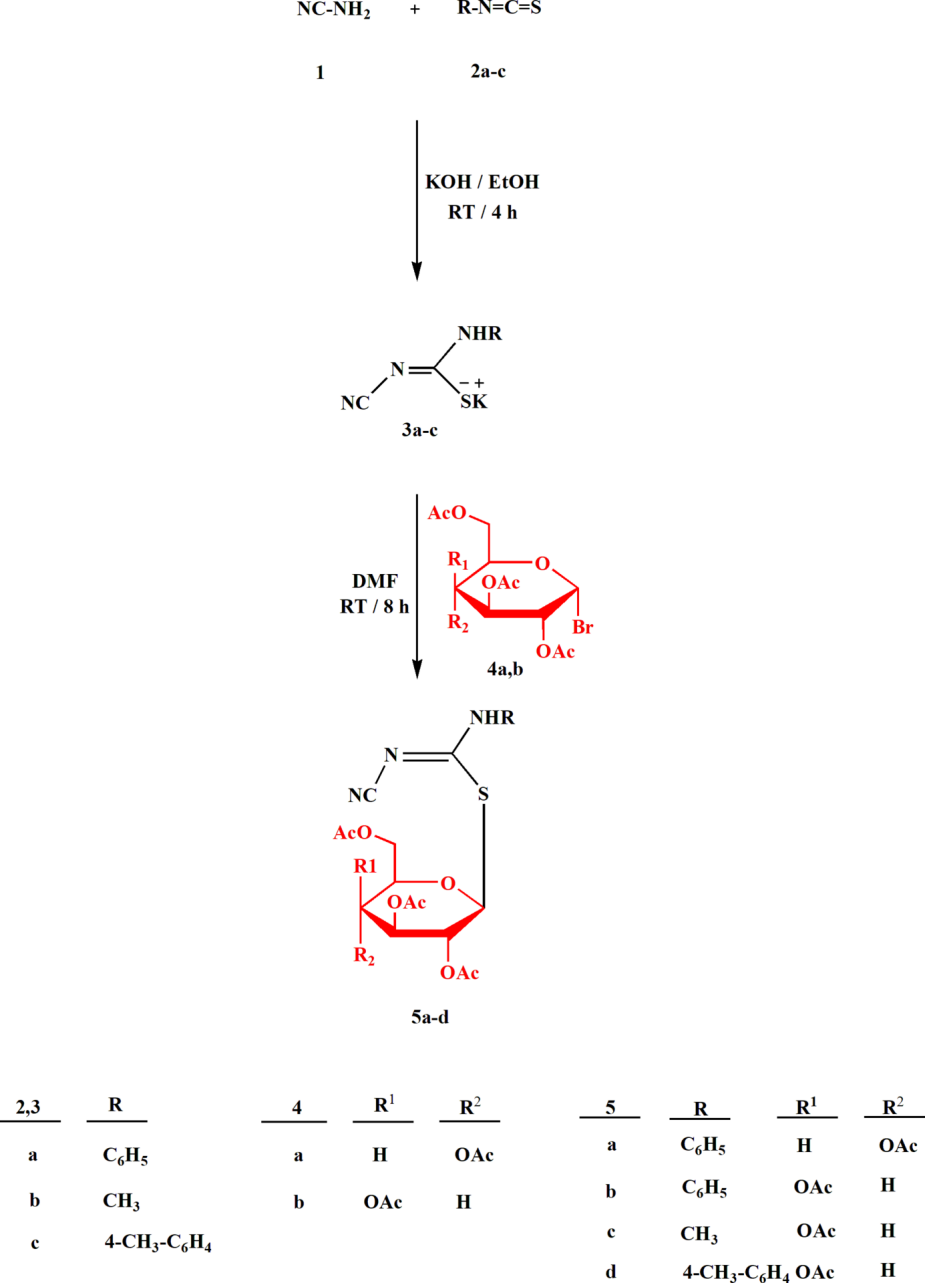
The reaction scheme for the syntheses of compounds **5a**–**d**.

This work reports the one-pot synthesis of glycosyl iso­thio­urea derivatives as a new class of acyclic thio­glycosides, the *N*′-cyano-*N-*(alkyl or ar­yl)carbamimido­thio­ates **5a**–**d**. The potassium 1-cyano-iso­thio­urea salts **3a**–**c** were chosen as the key reagents. The sequences of reactions are summarized in Fig. 1 ▸. Cyanamide **1** was heated with substituted iso­thio­cyanate derivatives **2a**–**c** in KOH/EtOH to give the corresponding stable potassium *N*-substituted carbamimido­thio­ates **3a**–**c**. These salts reacted with 2,3,4,6-tetra-*O*-acetyl-*α*-d-gluco- or galacto­pyranosyl bromides **4a**,**b** in DMF at room temperature to afford the corresponding *S*-glycosides **5a**–**d** in high yield. The structures of the compounds **5a**–**d** were established by their elemental analyses and spectroscopic data (see *Synthesis and crystallization*). For example, the ^1^H NMR spectra of **5a** showed the anomeric proton as a doublet at *δ* 5.82 ppm; the other six glucose protons resonated at *δ* 4.05–5.39 ppm and the four acetyl groups appeared as four singlets at *δ* 1.92–2.05 ppm. The structures of compounds **5a**–**d** were unambiguously confirmed by single-crystal X-ray structure determinations, which are reported here.
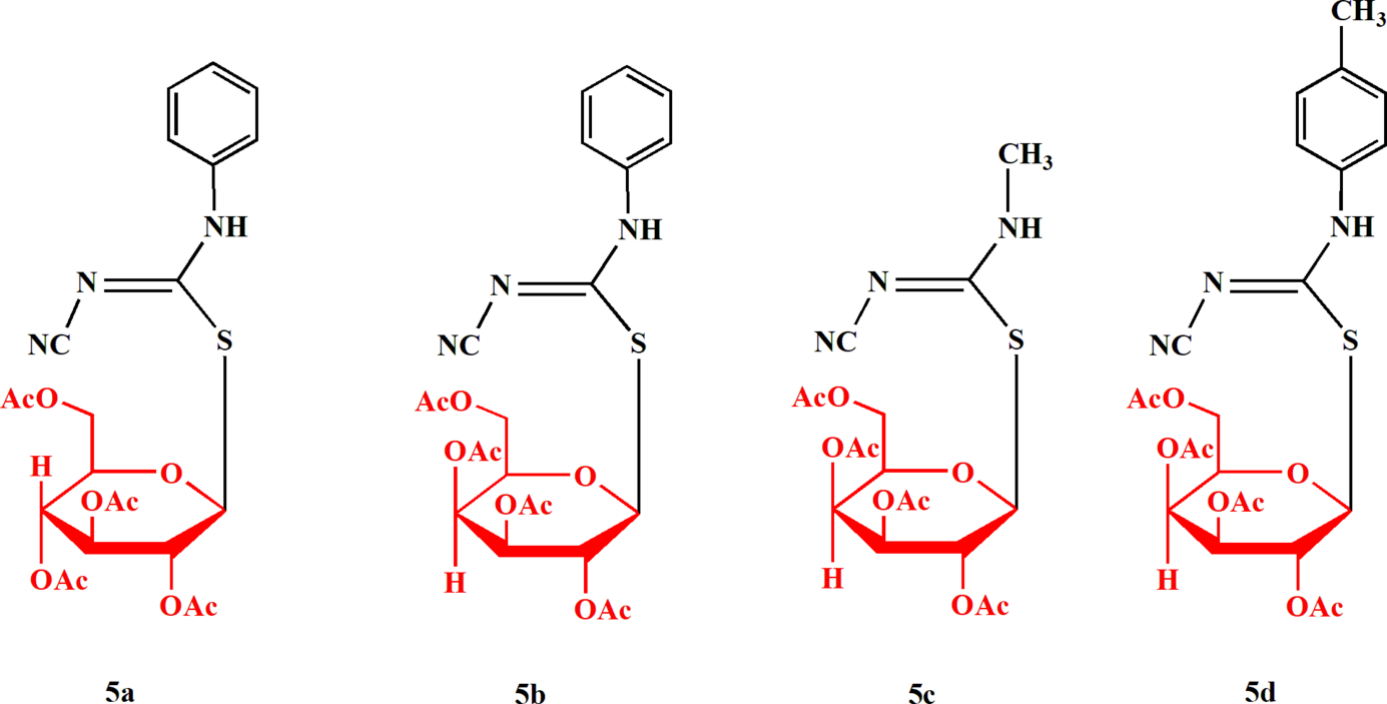


## 2. Structural commentary

**Figure 2:**
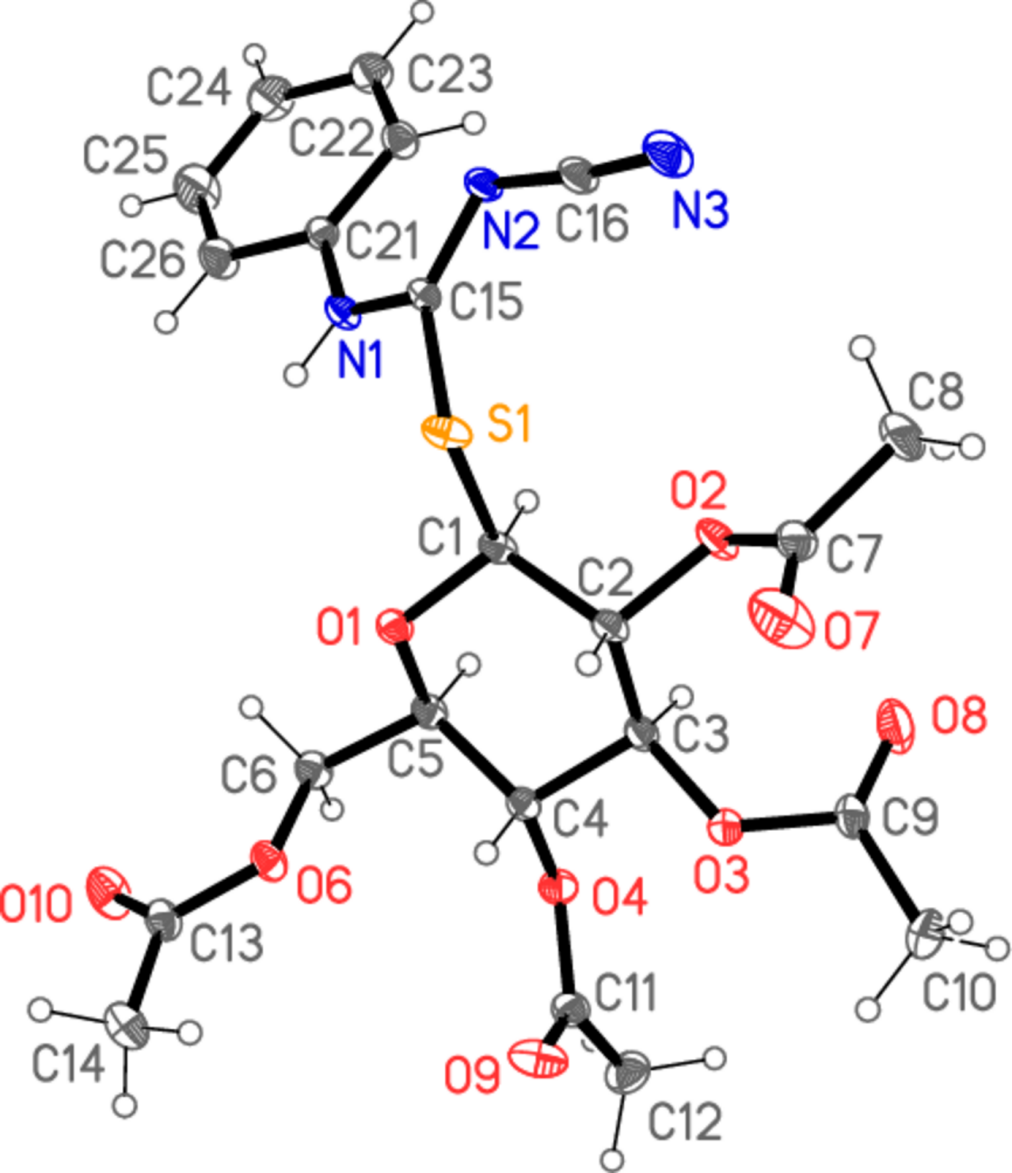
The mol­ecule of compound **5a** in the crystal. Ellipsoids represent 50% probability levels. Only the major site of the disordered acetyl group at O3 (atoms C9, C10, O8) is shown.

All four compounds **5a**–**d** crystallize solvent-free in space group *P*2_1_2_1_2_1_ with *Z* = 4. The mol­ecular structures are shown in Figs. 2 ▸–5 ▸ ▸ ▸, with selected mol­ecular dimensions in Tables 1 ▸–4 ▸ ▸ ▸. For all four structures, the configuration across the formal double bond C15=N2 is *Z*, with the cyano group and the sulfur atom mutually *cis*, which avoids a steric ‘collision’ between the cyano and aryl groups (where present). The absolute configurations at C1–C5 are *SRSSR* for **5a** and *SRSRR* for the other structures, the designations at C4 corresponding to the change of sugar from glucose in **5a** to galactose in **5b**–**d**. The torsion angles C5—O1—C1—S1 are all close to 180°, confirming the β (equatorial) positions of the substituent at the sugar ring. The C1—S1 bond lengths are consistently longer than C15—S1, corresponding to the different hybridizations of the carbon atoms. Compound **5b** involves an intra­molecular hydrogen bond from the NH group to the sugar oxygen atom O1, with H01⋯O1 = 2.11 (2) Å; in **5c** the longer H01⋯O1 distance, 2.52 (3) Å, represents the weaker branch of a three-centre inter­action, whereas in **5a** and **5d** the H01⋯O1 distances are even longer at 2.84 (2) and 2.90 (2) Å, respectively, and the NH group is thus effectively only involved in inter­molecular hydrogen bonds (see *Supra­molecular features*).

The carbamimido­thio­ate groups are consistently numbered as S1—C15 (—N1—C*xx*)=N2—C16≡N3, where *xx* is 21 for the aryl substituents but 17 for the methyl substituent. The six atoms (excluding C*xx*) are approximately coplanar, with r.m.s. deviations of 0.05, 0.03, 0.009 and 0.005 Å for **5a**–**d** in that order; the inter­planar angles to the aryl group are 20.04 (1), 47.89 (2) and 48.05 (4)°, respectively, for **5a**, **5b** and **5d**. The small inter­planar angle for **5a** is associated with a short intra­molecular H22⋯N2 contact of 2.34 Å. The sugar atom C1 lies 1.529 (1) Å out of the carbamimido­thio­ate plane for **5a**, 0.541 (1) Å for **5b**, 0.148 (2) Å for **5c** and 0.321 (2) Å for **5d**, corresponding to a wide range of C1—S1—C15—N1 torsion angles. The bond lengths are broadly as expected, in accordance with the different hybridizations of C*xx*. The near equality of bond lengths for C15—N1 (the aryl- or methyl-substituted nitro­gen) and the formal double bond C15=N2 (the cyano-substituted nitro­gen) indicate a considerable degree of delocalization in this region, as do the angle sums at N1 (359–360°) and the *sp*^2^ angles at C15, N1 and N2. The C15—N1 bond is slightly longer (by *ca* 0.02 Å) than C15—N2 except for the *N*-methyl derivative **5c**, where it is 0.07 Å shorter. The glucose derivative **5a**, however, shows some appreciable differences; thus the angle S1—C15—N1, 115.89 (4)°, is narrow, while C15—N1—C21 is very wide at 128.26 (5)°. Also, the angle at sulfur is appreciably narrower for **5a**, 100.24 (3)° compared to a mean value of 104.7° for **5b**–**d**. These differences, even between **5a** and **5b**, which have the same phenyl substituent at N1, can scarcely be attributed directly to the change of sugar, because the relevant atom C4 is quite remote from the affected carbamimido­thio­ate group. Similarly, the strong intra­molecular hydrogen bond in **5b** is absent in **5a**, but it is difficult to see how this would directly cause the observed differences.

A further explanation might be sought based on the torsion angles of the carbamimido­thio­ate groups. For all four compounds, the torsion angles S1—C15—N1—C*xx* corres­pond to an anti­periplanar geometry, and S1—C15—N2—C16 to synperiplanar. For the three galactose derivatives **5b**–**d**, the torsion angles O1—C1—S1—C15 are roughly constant at about −70°, whereas for **5a** the value is −101.53 (4)°; the groupings C1—S1—C15—N1 for **5b**–**d** are roughly synperiplanar (torsion angles 7–17°), but the value for **5a** is 57.56 (5)°. Finally, the torsion angles C15—N1—C21—C22 are widely different for **5a**, **5c** and **5d**, corresponding to different rotations of the aryl ring. Despite the many degrees of torsional freedom, it seems that the unusual values for some bond angles of **5a** may tentatively be connected with its lack of synperiplanarity for the region C1—S1—C15—N1. Without detailed calculations, however, this is difficult to prove (and takes no account of packing effects, see following section).

**Figure 3:**
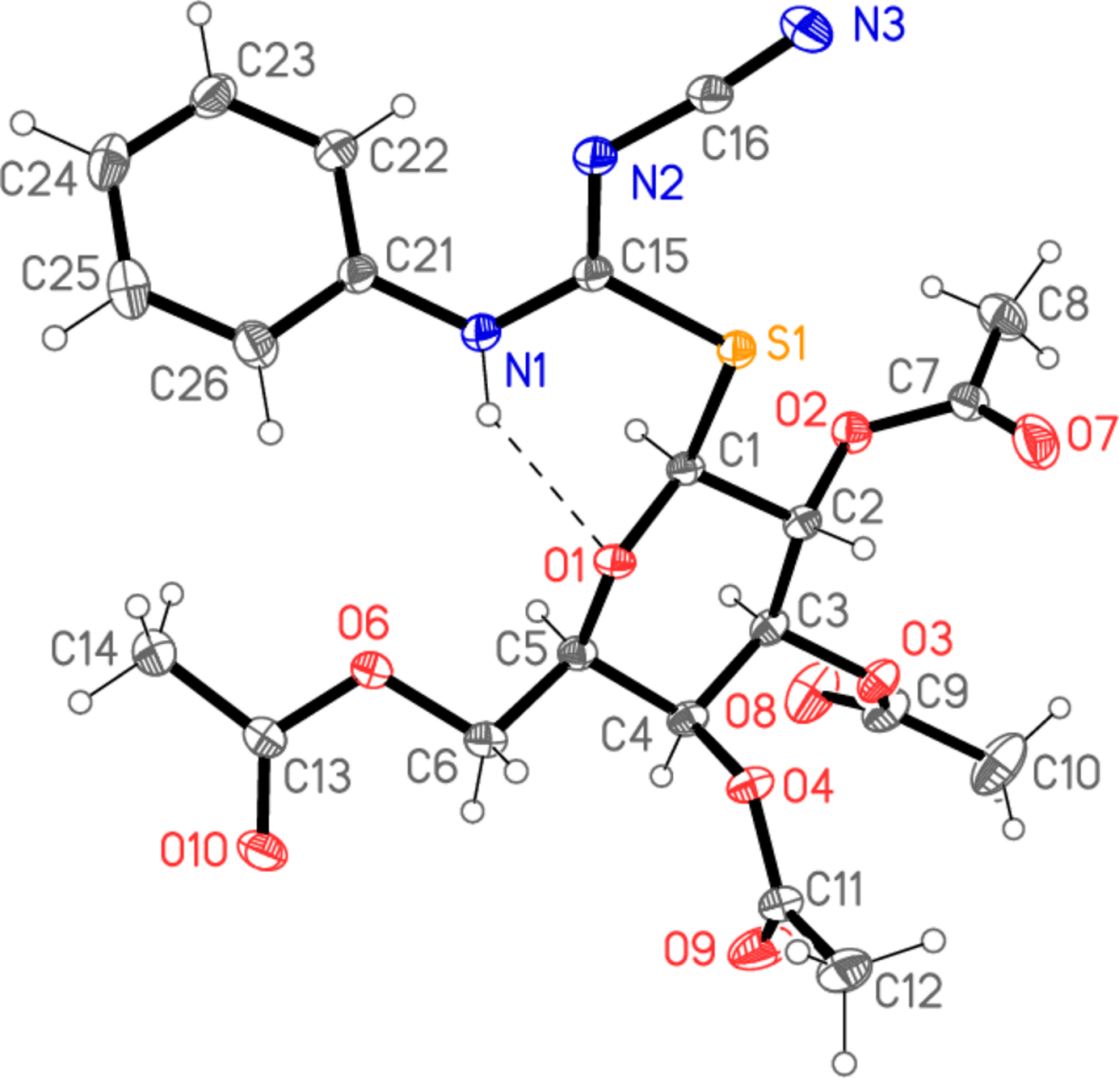
The mol­ecule of compound **5b** in the crystal. Ellipsoids represent 50% probability levels. The dashed line indicates an intra­molecular hydrogen bond.

In the acetyl­ated sugar moieties, all the C—O—C=O torsion angles of the acetyl groups correspond to a synperi­planar geometry, but otherwise these too display a high degree of conformational flexibility. For the galactoses, the torsion angles C3—C4—O4—C11, C1—C2—O2—C7 and (to a lesser extent) C2—C3—O3—C9 remain reasonably constant, but there are large differences in C4—C5—C6—O6, which is −167.08 (6)° for **5b**, where the extended configuration is clear in Fig. 3 ▸, but −63.41 (17)° for **5c** and −61.75 (13)° for **5d**. The glucose derivative **5a** necessarily deviates appreciably from **5b**–**d** in torsion angles involving the region at C4, where the configuration is reversed.

## 3. Supra­molecular features

All the compounds involve several potential hydrogen-bond acceptors *A* (nine oxygens, two nitro­gens and the sulfur; the nitrile nitro­gen atom is the acceptor for all three inter­molecular hydrogen bonds, see below) but only one classical hydrogen-bond donor (the NH group). This means that several C—H⋯*A* ‘weak’ hydrogen bonds might be expected, and this is indeed the case. For completeness, the hydrogen-bond tables (Tables 5 ▸–8 ▸ ▸ ▸) contain a number of borderline cases, not all of which are discussed. The packing diagrams are drawn to include only the shortest contacts, for the sake of clarity. The space group *P*2_1_2_1_2_1_ is well known for its propensity to provide complex three-dimensional packing patterns if the inter­actions involve more than one 2_1_ axis.

**Figure 6:**
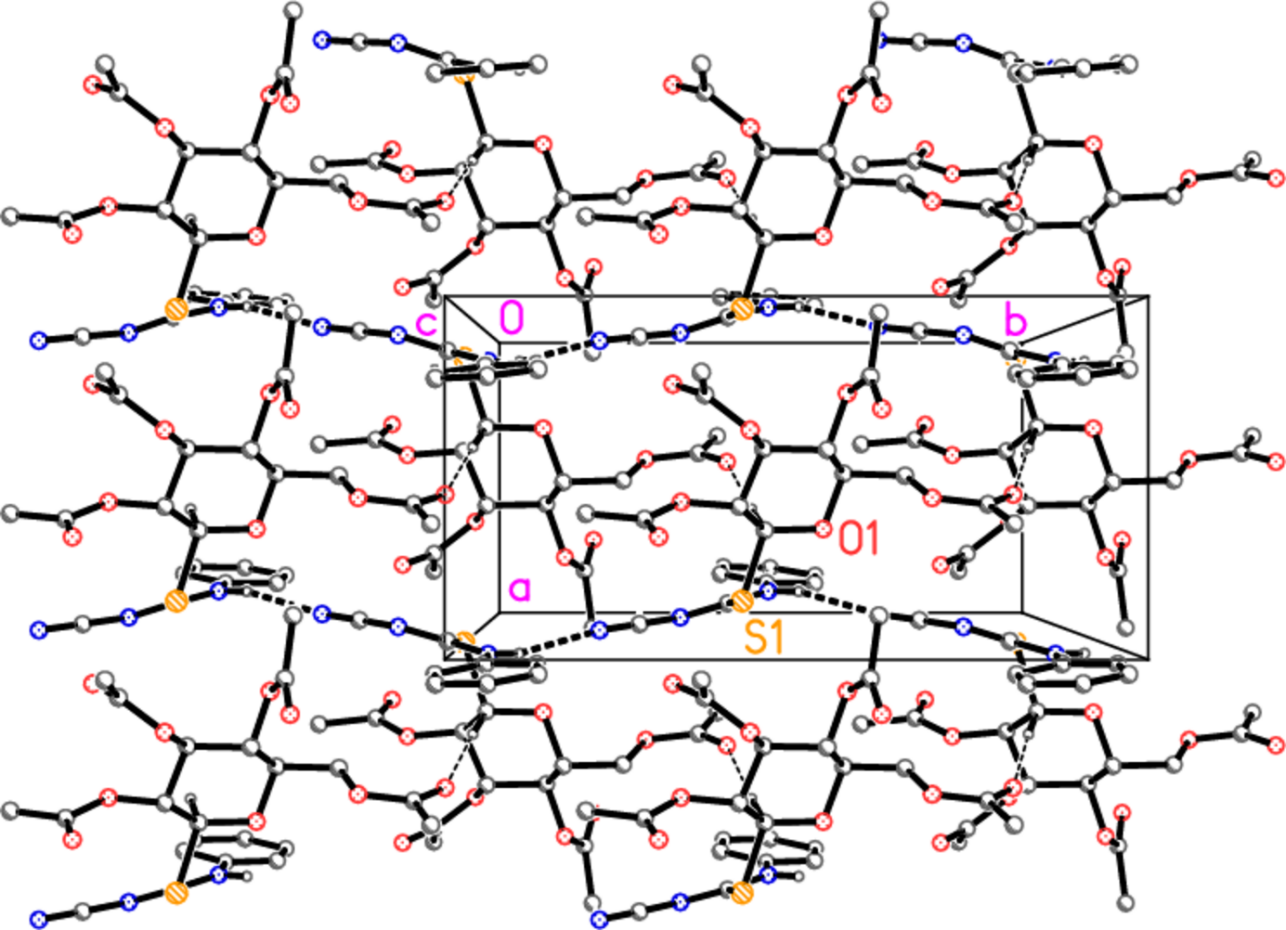
Packing diagram of compound **5a** viewed parallel to the *c* axis, showing one layer in the region *z* ≃ 0.25. Dashed lines indicate the hydrogen bonds H01⋯N3 (thick) and H1⋯O10 (thin). Hydrogen atoms not involved in hydrogen bonding are omitted for clarity. Two atoms are labelled to indicate the asymmetric unit.

**Figure 7:**
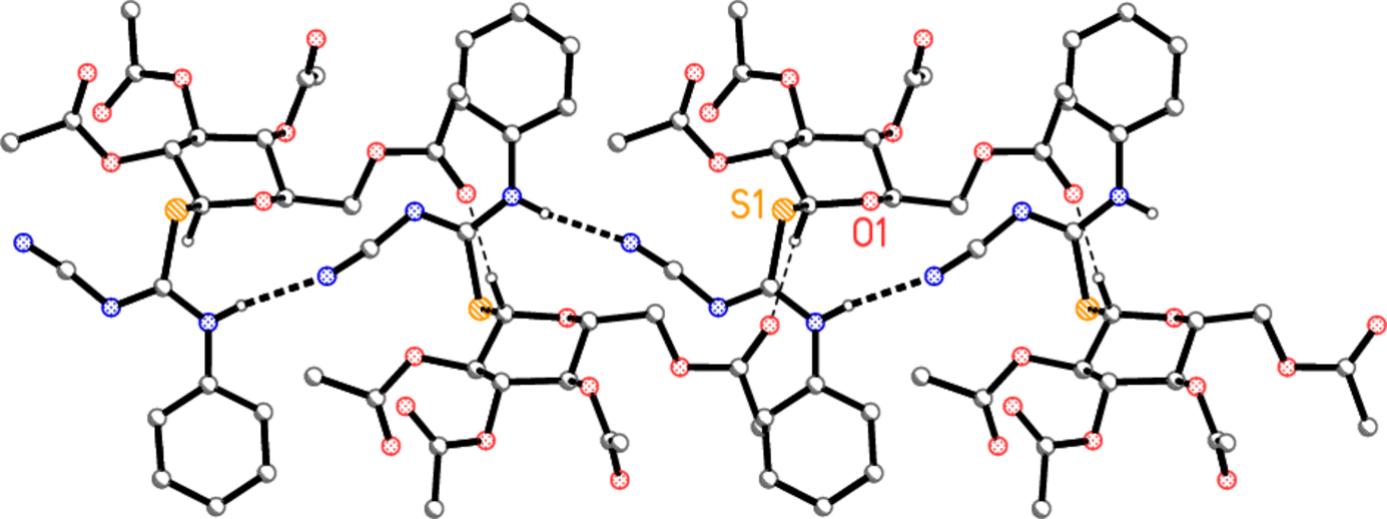
The layer from Fig. 6 ▸ is shown here in projection parallel to the *a* axis.

In compound **5a**, the classical hydrogen bond N1—H01⋯N3 and the ‘weak’ but short inter­action C1—H1⋯O10 combine via the 2_1_ axis parallel to *b* to form a layer structure parallel to the *ab* plane (Fig. 6 ▸). Because the layer is quite thick, a side view in projection parallel to the *a* axis is shown as Fig. 7 ▸ as an aid to inter­pretation.

**Figure 8:**
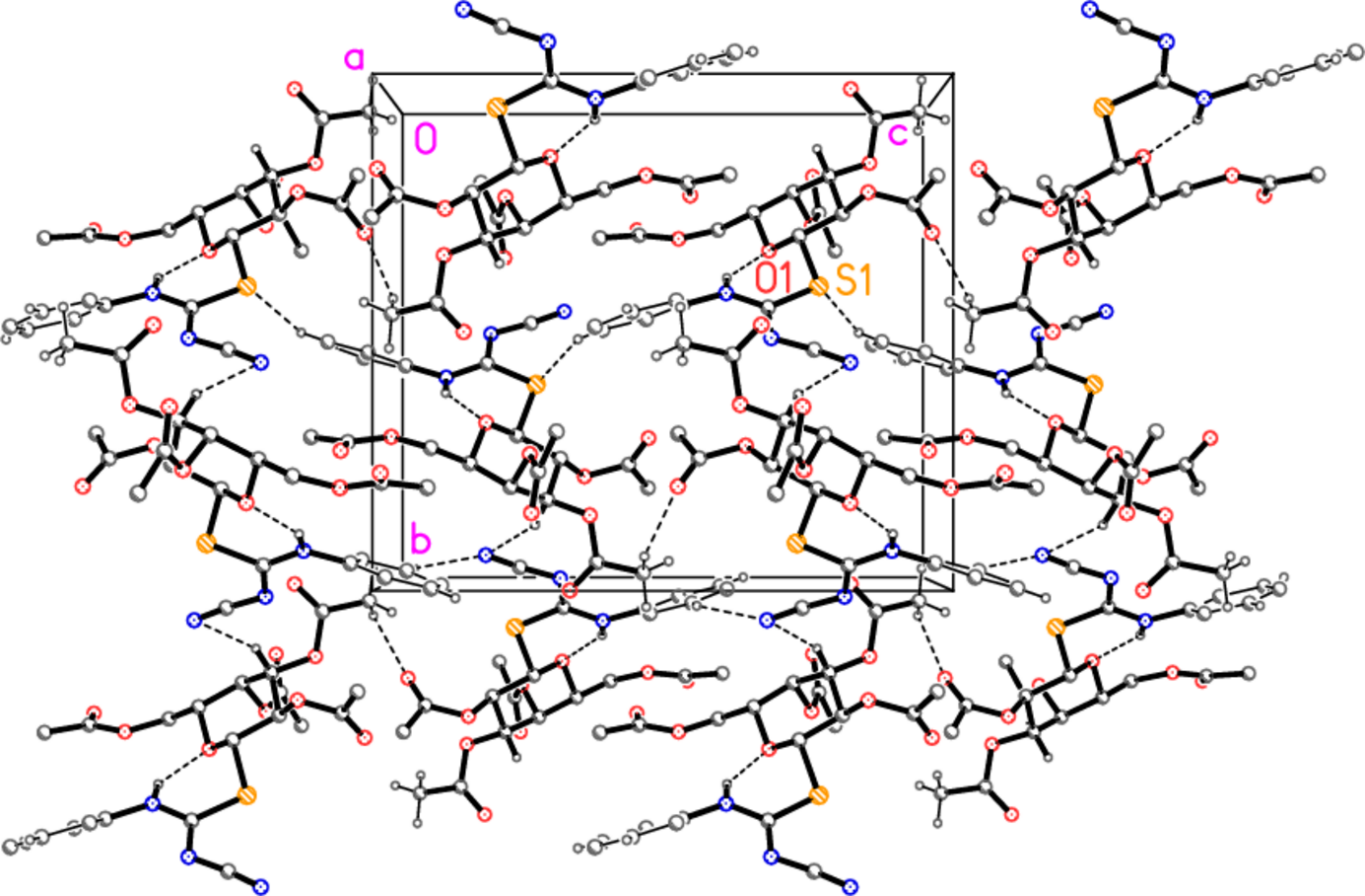
Packing diagram of compound **5b** viewed parallel to the *a* axis, showing the region *x* ≃ 0.75. Thin dashed lines indicate ‘weak’ hydrogen bonds. Hydrogen atoms not involved in hydrogen bonding are omitted for clarity. Two atoms are labelled to indicate the asymmetric unit.

The packing of compound **5b** involves no strikingly short (< 2.5 Å) H—*A* contacts; the NH group is involved in an intra­molecular hydrogen bond (see above), and its inter­molecular contact to O10, at 2.68 (2) Å and with an angle of only 101 (1)° at H01, can probably be neglected. We were unable to construct a clearly assimilable packing diagram, but Fig. 8 ▸ shows the pattern generated by the contacts H25⋯S1, H10*C*⋯O7, H3⋯N3 and H23⋯N3.

**Figure 9:**
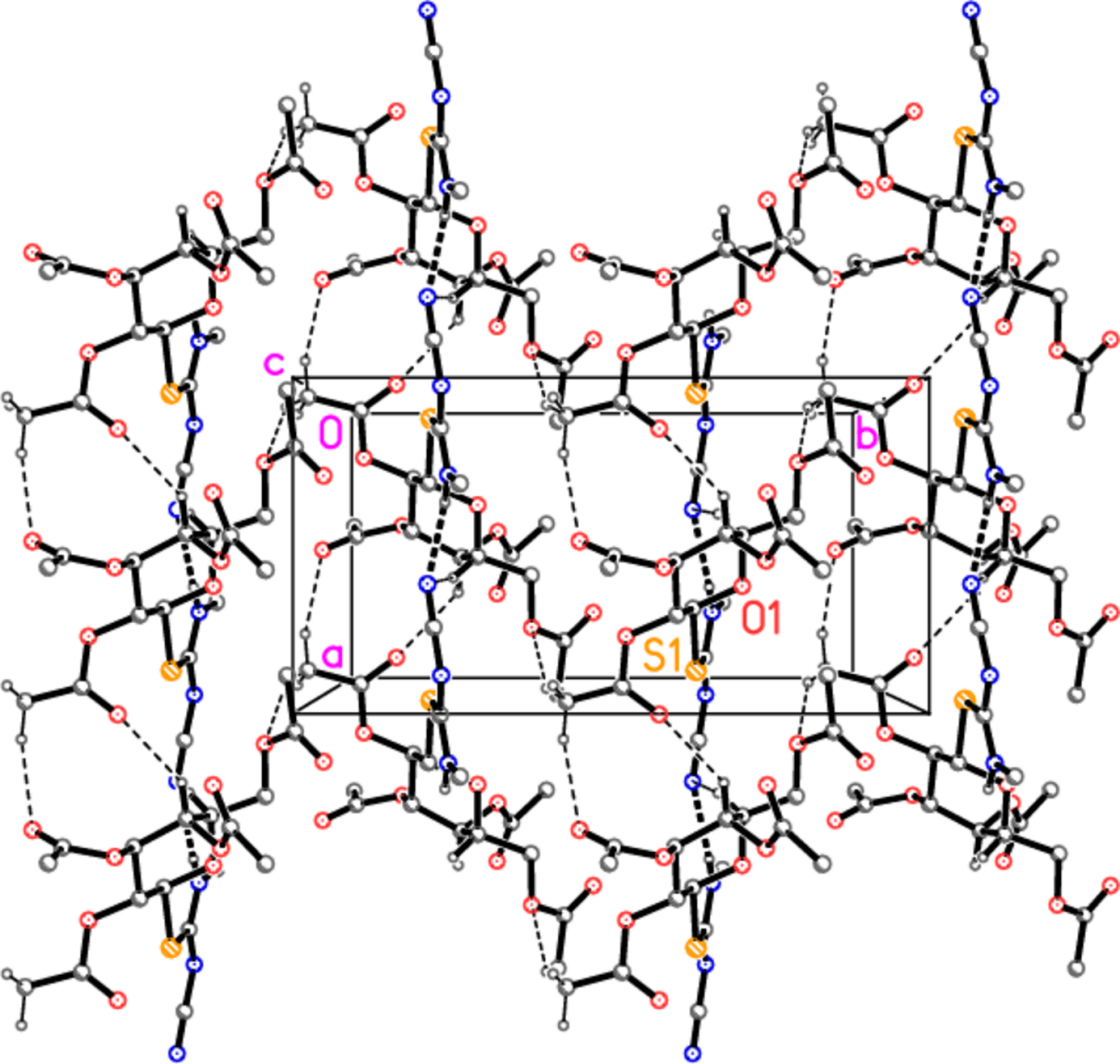
Packing diagram of compound **5c** viewed parallel to the *c* axis, showing one layer in the region *z* ≃ 0.25. Dashed lines indicate the hydrogen bonds H01⋯N3 (thick) and four H⋯O (thin). The longer contact H1⋯O10 is also present in this layer, but is omitted for clarity, as are hydrogen atoms not involved in hydrogen bonding. Two atoms are labelled to indicate the asymmetric unit.

**Figure 10:**
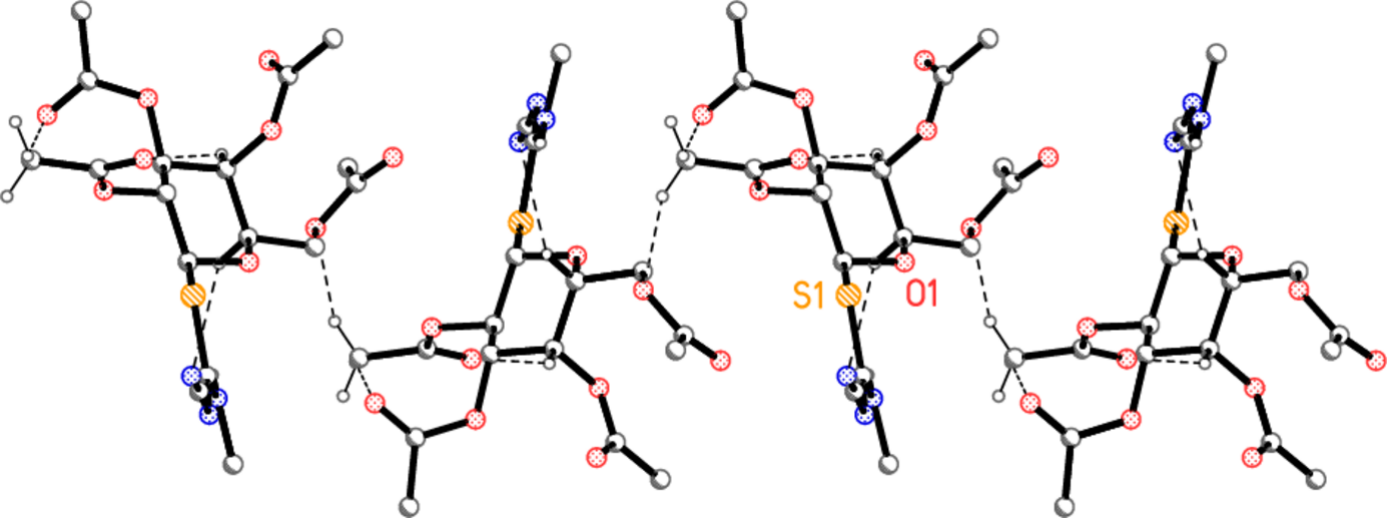
The layer from Fig. 9 ▸ is shown here in projection parallel to the *a* axis.

**Figure 11:**
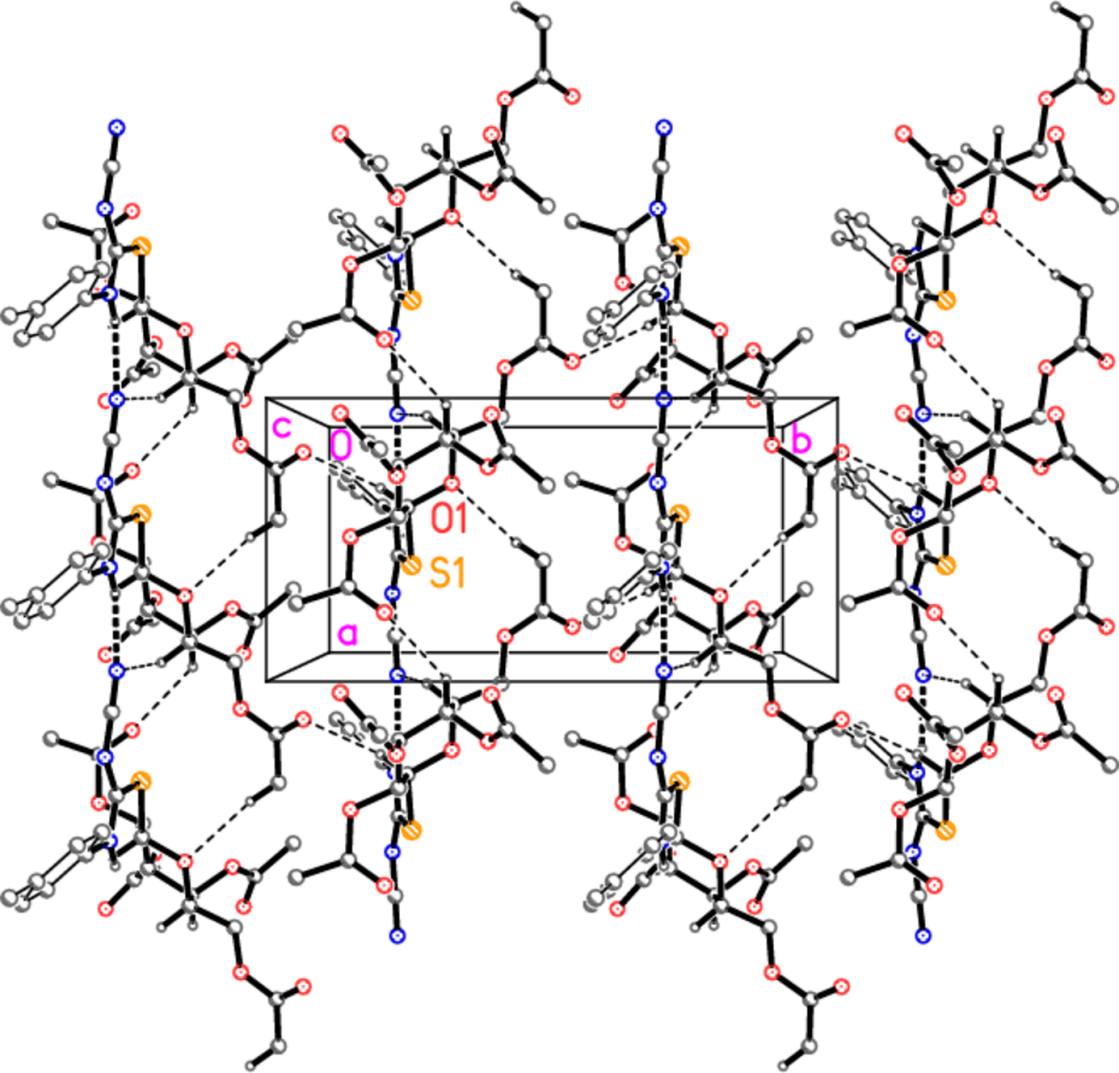
Packing diagram of compound **5d** viewed parallel to the *c* axis, showing one layer in the region *z* ≃ 0.75. Dashed lines indicate the hydrogen bonds H01⋯N3 (thick) and four H⋯O (thin). Hydrogen atoms not involved in hydrogen bonding are omitted for clarity. Two atoms are labelled to indicate the asymmetric unit.

In compound **5c**, the classical hydrogen bond H01⋯N3 combines with the four shortest ‘weak’ contacts to form a layer structure parallel to the *ab* plane (Fig. 9 ▸), and the same is true for compound **5d** (Fig. 11). Again, side views of the layers, in projection, are shown as Figs. 10 ▸ ▸ and 12 ▸, respectively.

## 4. Database survey

The searches employed the routine ConQuest (Bruno *et al.*, 2002 ▸), part of Version 2024.1.0 of the Cambridge Structural Database (Groom *et al.*, 2016 ▸). A search for the acyclic residue C^any^—S^2^—C^3^(—N^2^—C^2^—N^1^)—N^3^H—C^any^, where the superscripts indicate coordination numbers, found (apart from our own structure, Elgemeie *et al.*, 2015 ▸; refcode TOZCES) seven structures, namely: methyl *N*-(4-chloro­phen­yl)-*N*′-cyano­carbamimido­thio­ate (refcode EDETAJ; Lu, 2007 ▸); methyl *N*′-cyano-*N*-(4-meth­oxy­benz­yl)carbamimido­thio­ate and methyl *N*-(1-benzyl­piperidin-4-yl)-*N*′-cyano­carbamimido­thio­ate (OWAHIE and OWAHOK; Lu, 2011 ▸); methyl *N*′-cyano-*N*-(3-pyridinylmeth­yl)imido­thio­carbamate (XAZKIT; Lan *et al.*, 2006 ▸); methyl *N*-benzyl-*N*′-cyano­carbamimido­thio­ate and methyl *N*′-cyano-*N*-phenyl­carbamimido­thio­ate (FUZYIL and FUZXOQ; Wang *et al.*, 2020 ▸). In each case, the sulfur atom was bonded to a methyl group rather than the sugar residues in compounds **5a**–**d**. The structure of methyl *N*-(4-chloro­phen­yl)-*N*′-cyano­carb­amimido­thio­ate was reported again by Wang *et al.* (2020 ▸; EDETAJ01); inspection of the data indicates that this is a new measurement of the same structure (and not a new polymorph). Six of the structures show a *Z* configuration across the formal C=N double bond, but TOZCES is *E*-configured, with the cyano and SMe groups mutually *trans*. The bond lengths (Å) in the central SCN_2_ moiety were: S—C 1.742–1.768, av. 1.749 (9); C—N(CN) 1.31–1.326, av. 1.317 (5); C—NHC 1.311–1.349, av. 1.327 (13). This corresponds reasonably well to our values of 1.7593–1.7759, av. 1.7646; 1.3084–1.323, av. 1.3305; and 1.316–1.3382, av. 1.3155 Å. The six atoms of the carbamimido­thio­ate group are essentially coplanar in all these structures (maximum mean deviation 0.039 Å), but in two cases (OWAHOK and XAZKIT) the methyl group at sulfur is rotated out of the plane (by 0.84 Å).

## 5. Synthesis and crystallization

***General procedure for the synthesis of 5a–d***

The reaction scheme is given in Fig. 1 ▸. Cyanamide **1** (0.42 g, 0.01 mol) was added to a cold solution of potassium hydroxide (0.56 g, 0.01 mol) in absolute ethanol (20 mL) and the mixture was stirred for 10 min. The appropriate substituted iso­thio­cyanate derivative (**2a**, **2b** or **2c**; 0.01 mol), was then added gradually over a period of 15 min and the mixture was stirred at room temperature for 4 h, after which the reaction was complete. The solvent was evaporated under reduced pressure, and the residue was dissolved in DMF (15 mL). A solution of 2,3,4,6-tetra-*O*-acetyl-α-d-gluco- or galacto-pyranosyl bromide **4a** or **4b** (4.2 g, 0.01 mol) in DMF was then added dropwise over 30 min. Stirring was continued at room temperature until the reaction was judged complete by thin-layer chromatography (6–8 h). The mixture was poured into ice–water, and the resulting precipitate was collected by filtration, dried, and crystallized from ethanol to give compounds **5a**–**d**.

*(Z)-2′,3′,4′,6′-Tetra-O-acetyl-β-D-gluco­pyranosyl N′-cyano-N-phenyl­carbamimido­thio­ate (**5a**)*

Colourless crystals, yield 4.31 g (85%). M.p. 427–428 K; IR (KBr, cm^−1^) 3444 (NH), 3027 (aromatic CH), 2198 (CN), 1758 (4 × C=O), 1608 (C=N); ^1^H NMR (400 MHz, DMSO-*d*_6_): *δ* 1.92, 1.97, 2.00, 2.05 (4*s*, 12H, 4 × OAc), 4.05 (*dd*, 1H, *J* = 12.0, 1.6 Hz, H-6′), 4.15–4.22 (*m*, 2H, H-6", H-5′), 5.02–5.09 (*m*, 2H, H-3′, H-4′), 5.39 (*t*, 1H, *J* = 9.6 Hz, H-2′), 5.82 (*d*, 1H, *J* = 10.0 Hz, H-1′), 7.29 (*t*, 1H, *J* = 7.2 Hz, Ar-H), 7.43 (*t*, 2H, *J* = 8.4 Hz, Ar-H), 7.45 (*d*, 2H, *J* = 8.0 Hz, Ar-H), 10.51 (*br. s*, D_2_O exch., 1H, NH); ^13^C NMR (100 MHz, DMSO-*d*_6_): *δ* 20.73–20.86 (4 × CH_3_CO), 62.21 (C-6′), 68.06 (C-4′), 69.91 (C-2′), 73.07 (C-3′), 75.63 (C-5′), 81.66 (C-1′), 114.37 (CN), 124.30 (2C, Ar-C), 127.24 (Ar-C), 129.48 (2C, Ar-C), 137.44 (Ar-C), 163.57 (C=N), 169.77–170.46 (4 C=O). Analysis calculated for C_22_H_25_N_3_O_9_S (507.51): C 52.06, H 4.97, N 8.28, S 6.32. Found: C 52.16, H 4.88, N 8.18, S 6.22%.

*(Z)-2′,3′,4′,6′-Tetra-O-acetyl-β-D-galacto­pyranosyl N′-cyano-N-phenyl­carbamimido­thio­ate (**5b**)*

Colourless crystals, yield 4.52 g (89%). M.p. 453–454 K; IR (KBr, cm^−1^) 3440 (NH), 3023 (aromatic CH), 2221 (CN), 1724 (4 × C=O), 1592 (C=N); ^1^H NMR (400 MHz, DMSO-*d*_6_): *δ* 1.89, 1.99, 2.03, 2.05 (4*s*, 12H, 4 × OAc), 3.99–4.25 (*m*, 3H, H-6′, H-6", H-5′), 5.22–5.27 (*m*, 2H, H-4′, H-2′), 5.41 (*dd*, 1H, *J* = 9.4, 2.8 Hz, H-3′), 5.65 (*d*, 1H, *J* = 10.2 Hz, H-1′), 7.14 (*br. t*, 2H, *J* = 7.6 Hz, Ar-H), 7.44 (*br. t*, 2H, *J* = 8.2 Hz, Ar-H), 7.48 (*br. d*, 1H, *J* = 8.1Hz, Ar-H), 10.44 (*s*, D_2_O exch., 1H, NH). Analysis calculated for C_22_H_25_N_3_O_9_S (507.51): C 52.06, H 4.97, N 8.28, S 6.32. Found: C 52.18, H 4.80, N 8.15, S 6.32%.

*(Z)-2′,3′,4′,6′-Tetra-O-acetyl-β-D-galacto­pyranosyl N′-cyano-N-methyl­carbamimido­thio­ate (**5c**)*

Colourless crystals, yield 3.96 g (89%). M.p. 468–469 K; IR (KBr, cm^−1^) 3455 (NH), 2942 (CH), 2175 (CN), 1751 (4 × C=O), 1627 (C=N); ^1^H NMR (400 MHz, DMSO-*d*_6_): *δ* 2.01, 2.03, 2.07, 2.14 (4*s*,12H, 4 × OAc), 2.88 (*d*, 3H, *J* = 4.4 Hz, CH_3_), 4.02–4.09 (*m*, 2H, H-6′, H-6"), 4.37 (*t*, 1H, *J* = 6.0 Hz, H-5′), 5.09 (*t*, 1H, *J* = 10.0 Hz, H-2′), 5.26 (*dd*, 1H, *J* = 7.8, 3.2 Hz, H-3′), 3.37 (*t*, 1H, *J* = 2.8 Hz, H-4′), 5.57 (*d*, 1H, *J* = 10.0 Hz, H-1′), 8.42 (*br. s*, D_2_O exch., 1H, NH); ^13^C NMR (100 MHz, DMSO-*d*_6_): *δ* 20.77–20.97 (4 × CH_3_CO), 30.95 (CH_3_), 61.84 (C-6′), 67.03 (C-4′), 67.69 C-2′), 71.04 (C-3′), 74.76 (C-5′), 81.17 (C-1′), 115.46 (CN), 164.60 (C=N), 169.86–170.40 (4 C=O). Analysis calculated for C_17_H_23_N_3_O_9_S (445.44): C 45.84, H 5.20, N 9.43, S 7.20. Found: C 45.76, H 5.12, N 9.34, S 7.13%.

*(Z)-2′,3′,4′,6′-Tetra-O-acetyl-β-D-galacto­pyranosyl N′-cyano-N-p-tolyl­carbamimido­thio­ate (**5d**)*

Colourless crystals, yield 4.43 g (85%). M.p. 456–457 K; IR (KBr, cm^−1^) 3440 (NH), 3054 (aromatic CH), 2950 (CH), 2210 (CN), 1748 (4 × C=O), 1592 (C=N); ^1^H NMR (400 MHz, DMSO-*d*_6_): *δ* 1.91, 1.94, 2.01, 2.08 (4*s*, 12H, 4 × OAc), 2.30 (*s*, 3H, CH_3_), 4.07–4.12 (*m*, 2H, H-6′, H-6"), 4.41–4.48 (*m*, 1H, H-5′), 5.16 (*t*, 1H, *J* = 9.6 Hz, H-4′), 5.33 (*t*, 2H, *J* = 14.4, 9.6 Hz, H-3′, H-2′), 5.71 (*d*, 1H, *J* = 10.0 Hz, H-1′), 7.23 (*d*, 2H, *J* = 7.2 Hz, Ar-H), 7.39 (*d*, 2H, *J* = 6.4 Hz, Ar-H), 10.30 (*br. s*, D_2_O exch., 1H, NH). Analysis calculated for C_23_H_27_N_3_O_9_S (521.54): C 52.97, H 5.22, N 8.06, S 6.15. Found: C 52.88, H 5.15, N 8.16, S 6.10%.

## 6. Refinement

Crystal data, data collection and structure refinement details are summarized in Table 9 ▸.

The hydrogen atoms of the NH groups were refined freely. The methyl groups were included as idealized rigid groups allowed to rotate but not tip (command ‘AFIX 137’), with C—H = 0.99 Å and H—C—H = 109.5°. Other hydrogen atoms were included using a riding model starting from calculated positions (C—H_methyl­ene_ = 0.99, C—H_methine_ = 1.00, C—H_arom_ = 0.95 Å). The *U*(H) values were fixed at 1.5 × *U*_eq_ of the parent carbon atoms for the methyl group and 1.2 × *U*_eq_ for other hydrogens.

For compound **5a**, the acetyl group at O3 (atoms C9, C10, O8) was disordered over two positions. The occupation factor of the minor component refined to 0.085 (2). Appropriate restraints were employed to improve refinement stability, but the dimensions of disordered groups (and particularly the minor components) should always be inter­preted with caution. In the discussion sections above, the minor component is not considered.

Badly fitting reflections were omitted as follows: **5a**, seven reflections with deviations > 8σ; **5b**, five reflections with deviations > 8σ; **5c**, one reflection with deviation > 10σ; **5d**, three reflections with deviations > 7σ.

## 7. Appendix: The choice of radiation type for X-ray measurements

The large and well-formed crystals of compounds **5a** and **5b** were clearly suitable for measurements using Mo *K*α radiation; **5c** consisted of smaller crystals, and one of these was measured with Cu radiation. For compound **5d**, we originally recorded a dataset using Cu *K*α radiation; data for this are given in Table 9 ▸ in the final column ‘**5d**(Cu)’. The reasons for this, and for preferring the Mo dataset (measured later with the same crystal), will be discussed here (together with a potted history of the fashions in X-ray wavelengths over the last 50 years, as experienced by PGJ) as they may be of general inter­est, in particular to younger crystallographers. One central criterion is the ability to determine absolute configuration (more generally ‘absolute structure’, see below) and the other, connected with this, is the need to collect data of adequate intensity.

The first automated four-circle diffractometers were introduced in the early 1970s, and the institute where I was working acquired such a diffractometer (shared by both chemistry departments) in 1974; the tubes were changed from copper (henceforth Cu) to molybdenum (henceforth Mo) radiation or *vice versa* every six months. These were the only types of X-ray tube generally available at the time. As an X-ray beginner, I was told that Cu radiation was used for organic structures and Mo radiation for inorganic structures. As I soon realised, this is an oversimplification; a more accurate formulation would be that Cu radiation is used for crystals that diffract less strongly and Mo for those that diffract more strongly, because Cu radiation has a higher intrinsic intensity (main beam intensities were much weaker then; the nominal diameter of the main beam for Mo measurements was *ca* 0.7 mm, and the chosen crystals were often of this size, unless they were highly absorbing materials, in order to maximize the measured intensities). This was brought home to me by a typical beginner’s mistake, my unwise measurement of a large crystal of the ‘organic’ compound sodium acetyl­phospho­nate acetic acid solvate using Cu radiation; the diffraction was extremely strong, but the absorption and (particularly) extinction effects were so pronounced that the structure was unusable, and had to be repeated using Mo radiation (Jones & Kennard, 1978 ▸), which, with its shorter wavelength, is absorbed less strongly. Like many oversimplifications, the assumption that inorganic materials (including metal complexes) diffract more strongly than organic materials has some validity; the scattering power of a crystal of a given size will depend on the number of electrons in the crystal, which in turn depends on the density, and densities are generally greater for inorganic materials (a second-order effect is that their *U* values tend to be lower).

One important reason for using Mo radiation is to reduce absorption effects, because absorption corrections at the time largely relied on face-indexing the crystals, a procedure that was often difficult or impossible. The quality of datasets increased significantly when the first generalized absorption correction methods became available; this major step was provided by Flack (1974 ▸), who introduced the ψ-scan method to diffractometry. Nowadays, the highly redundant datasets (see below) have made the ‘multi-scan’ (Blessing, 1995 ▸) the method of choice. The use of Cu radiation decreased drastically during and after the 1970s, which is partly attributable to the more serious absorption effects, but also to the limited amount of data that could be measured; a complete sphere of Cu reflections was inaccessible because of the restricted geometry of bulky diffractometer components (typically, measurements above 2θ = 120° were difficult to obtain) and even a complete sphere of Cu data to the theoretical limit of 180° would only correspond to 2θ = 55° using Mo radiation. Routine measurements were usually conducted at room temperature with Mo radiation and 2θ_max_ = 50°; above this value, significant intensity was difficult to detect for many organic and organometallic samples.

The other connection between Cu radiation and organic crystals is associated with enanti­omerically pure materials, of which many natural products constitute an important sub-class; the determination of absolute configuration, often of great importance for these materials, relies on the measurement of generally small intensity differences between Friedel opposite reflections *hkl* and −*h*, −*k*, −*l*, caused by the phenomenon ‘anomalous dispersion’ (perhaps not a well-chosen name, because physicists tell us that there is nothing anomalous about it); the longer the wavelength, the more pronounced are these effects. The correct absolute configuration should then give a better *R* value than the incorrect, inverted, structure. Measurable differences at the time could only be detected in the presence of heavy atoms (as a rule of thumb, elements of the fourth or higher periods), and the effects were generally unobservable for light-atom structures, so that it was often necessary to synthesize heavy-atom derivatives of natural products in order to determine their absolute configuration. It should also not be forgotten that direct methods in the early 1970s were still in their infancy and often unreliable (especially for weak datasets), and a heavy-atom derivative was often needed to solve the structure in the first place (by the Patterson method).

As an example of an old structure determination of a natural product, the peptide l-serylglycine (Jones *et al.*, 1978 ▸) shows the standard practices of the time. The structure was measured using Cu radiation (for a week) to a 2θ_max_ value of only 116°. A total of 1186 intensities were measured, giving 713 unique data > 4σ(*F*); weak reflections were omitted from the measurement. Friedel opposites were presumably not merged, but the data are no longer directly available. The number of parameters is not given, but must have been roughly 125. There is no mention of the absolute configuration. From a modern viewpoint, albeit nearly 50 years on and with hindsight, this seems embarrassingly lackadaisical.

In general, *any* non-centrosymmetric structure must be compared with the corresponding inverted structure to ensure that the structure is correctly refined; the procedure is not confined to the space groups adopted by enanti­omerically pure materials (the ‘Sohncke’ space groups such as *P*2_1_2_1_2_1_; these were often informally and incorrectly called the ‘chiral’ space groups, but this name should now be used for the space groups that occur in pairs with opposite sense of the screw axis, such as *P*3_1_ and *P*3_2_ – formerly known as ‘enanti­omorphic’ space groups). The general procedure is now normally referred to as the ‘determination of absolute structure’, as suggested by Jones (1984*a* ▸), although the use of the word ‘absolute’ has correctly been questioned by Glazer & Stadnicka (1989 ▸).

For datasets where the determination of absolute structure was not expected to succeed, some bad habits were common (Jones, 1984*b* ▸, 1986 ▸); either the Friedel opposite reflections −*h*, −*k*, −*l* were not measured at all (because the space group, often determined only after data collection, was assumed to be centrosymmetric, or to save diffractometer time at a time when measurements were very slow by today’s standards), or the Friedel pairs *hkl* and −*h*, −*k*, −*l* were considered exactly equivalent and were merged (in *SHELX* using the command ‘MERG 3’, which is now effectively banned).

The determination of absolute structure/configuration relies on the existence of a method to test which configuration gives a significantly better fit. The ‘Hamilton *R* method’ (Hamilton, 1965 ▸) was the first statistical test to be generally used, but the results were capable of misinter­pretation. The first significant improvement was made by Rogers (1981 ▸; see also Jones, 1984*a* ▸), by refining a factor η that multiplied the anomalous scattering parameters *f*". The correct structure should then give an η value of +1 and the incorrect (inverted) structure a value of −1. This method gave a standard deviation for η, so that the reliability of the determination could also be judged. The next improvement was introduced by Flack (1983 ▸; reviewed by Watkin & Cooper, 2020 ▸), using the parameter *x* to estimate the extent of inversion twinning, whereby both the parent structure and the inverted structure are present in the same crystal. This had mathematical advantages over the η method and became the accepted method of determining absolute configuration; a value of *x* = 0 indicated the correct structure and 1 the incorrect (inverted) structure. The latest improvement was provided by Parsons *et al.* (2013 ▸; see also Parsons, 2017 ▸), who used the quotients [(*I*^+^) − (*I*^−^)]/[(*I*^ + ^) + (*I*^−^)] (where *I*^+^ is the intensity of *hkl* and *I*^−^ the intensity of −*h*, −*k*, −*l*) to improve the sensitivity with which the *x* parameter could be determined. The excellent review article by Linden (2017 ▸) on the determination of absolute structure was published just before the Parsons method became generally known.

In the 1990s, the use of area detectors increased the speed and precision of intensity measurements. Whereas the older ‘serial’ diffractometers measured one reflection at a time, and a dataset, even if consisting of only the independent data, took days or weeks to record, it was now possible to measure tens or hundreds of reflections per exposure (‘frame’). Datasets typically consist of hundreds of frames, whereby each reflection is measured many times; the redundancy leads to a statistical improvement in data precision (by merging many equivalents of each reflection) and enables the ‘multi-scan’ absorption correction. Continuous improvements in detector sensitivity and source intensity have brought measurement times down to hours rather than days.

The second, vital, development in the 1990s was the development of routine measurements at low temperature (without the restriction of severe icing problems). This is largely attributable to the efforts of Stalke. The advantages are well-known: the most important is the reduction of thermal motion, which in turn reduces the *U* values and thereby leads to an increase in the number and intensity of reflections that are available at higher angle (see *e.g.* Kottke & Stalke, 1993 ▸, and references therein). In our opinion, any X-ray structure determination at room temperature (in the absence of extenuating circumstances such as phase changes at low temperature) represents a missed opportunity to collect good data. A recent issue of *Acta Cryst. E* contained 21 low-temperature and 16 room-temperature structures.

The third major change was the use of refinements based on *F*^2^ rather than *F*, and using all data, including the weak reflections; this was introduced into the *SHELX* program system in the 1990s (Sheldrick, 2008 ▸, 2015*a* ▸,*b* ▸). It is fitting to pay tribute here to George Sheldrick, who has developed and maintained *SHELX* for some 50 years, and has always been quick to incorporate the newest developments (*e.g.* the Parsons method).

In the 2000s, the introduction of microsources, with typical beam diameters of 0.1–0.2 mm, for Cu radiation appreciably increased the available intensity. This had two important consequences. First, structures from weakly diffracting organic crystals with average dimensions as small as 10–50 µm, previously considered unmeasurable, could now be successfully determined (*e.g.* Abu-Zaied *et al.*, 2024 ▸); secondly, the anomalous scattering of oxygen atoms, previously regarded as negligible, was now often sufficient to determine the absolute structure reliably. The less bulky detectors and a favourable modified kappa geometry also meant that data could be collected to much higher angles (currently 2θ_max_ ≃ 160°). This led to a renaissance in the use of Cu radiation.

It was first recognized by Escudero-Adán *et al.* (2014 ▸) that the absolute configuration of light-atom structures could be determined reliably even using Mo radiation, if high-energy sources were used and the datasets were recorded at low temperature to higher (by the standards of the time) diffraction angles 2θ, typically 55°. The reason is that the anomalous scattering is approximately independent of 2θ, whereas the normal scattering decreases with increasing 2θ, so that the contribution of the anomalous scattering becomes more pronounced at high angles. The problem was that few light-atom structures diffracted to sufficiently high angles, but the Parsons method has made matters easier in this respect; thus we found that a steroid derivative, containing four oxygen atoms as anomalous scatterers, measured by us using a standard Mo source to 2θ = 61° (cholest-5-en-3-yl 3-formyl­phenyl carbonate, C_35_H_50_O_4_; refcode LUCVOX; Jones & Kuś, 2020 ▸), gave a correct (known) absolute configuration with *x* = 0.15 (16), whereas the *x* value without the Parsons modification had been indeterminate. The development of Mo microsources has helped further; with these, light-atom crystals can diffract significantly to 80° or more, and the absolute structure can then often be determined successfully with Mo radiation even for light-atom structures. The improvements in detector sensitivity have also made an important contribution.

Returning finally to the two datasets measured for **5d**, we originally thought that the very small crystals would need to be measured using Cu radiation. Although the absolute configuration of this galactose derivative is known, a confirmation using X-ray methods is always welcome. The crystal diffracted so strongly with Cu radiation, however, that we decided to re-measure the same crystal using Mo radiation. For practical purposes, both measurements were designed to run until the following day; the measurement times were *ca* 6 h for the Cu dataset and 22 h for the Mo dataset. Both datasets could certainly have been measured significantly faster, had it been necessary. The diffraction pattern for Mo extended to (at least) 2θ = 72° (in our experience, the intensity statistics of the data reduction often indicate that significant intensity is still present at angles where no maxima can be recognised in the frames), and the Flack *x* parameter is unambiguous at 0.005 (18). [Of course, the presence of sulfur, which nowadays counts as a ‘heavy’ atom, greatly facilitates the determination of the *x* parameter; this is usually no problem with elements of the third period. A good example of a structure with no atom heavier than oxygen is l-arabinose, which we measured to 2θ = 157° using Mo radiation, and which gave an *x* value of 0.03 (11) (refcode ABINOS04; Jones, 2023 ▸)]. The number of independent intensities is doubled compared to the Cu data (11008, *cf*. Cu 5508), so that the s.u.’s of mol­ecular dimensions are somewhat lower (by a factor of approximately 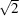, as would be expected if other things are equal). Furthermore, despite the generally effective absorption corrections that are now employed, it should not be forgotten that absorption effects are lower with Mo radiation. We therefore prefer the Mo dataset for **5d**, and would indeed recommend that, even for small organic crystals, the use of Mo radiation should not be dismissed out of hand. Both datasets are included here and are thus available to the inter­ested reader.

## Supplementary Material

Crystal structure: contains datablock(s) 5a, 5b, 5c, 5d, 5dCu, global. DOI: 10.1107/S2056989024006455/yz2056sup1.cif

Structure factors: contains datablock(s) 5a. DOI: 10.1107/S2056989024006455/yz20565asup2.hkl

Structure factors: contains datablock(s) 5b. DOI: 10.1107/S2056989024006455/yz20565bsup3.hkl

Structure factors: contains datablock(s) 5c. DOI: 10.1107/S2056989024006455/yz20565csup4.hkl

Structure factors: contains datablock(s) 5d. DOI: 10.1107/S2056989024006455/yz20565dsup5.hkl

Structure factors: contains datablock(s) 5dCu. DOI: 10.1107/S2056989024006455/yz20565dCusup6.hkl

CCDC references: 2367765, 2367766, 2367767, 2367768, 2367769

Additional supporting information:  crystallographic information; 3D view; checkCIF report

## Figures and Tables

**Figure 4:**
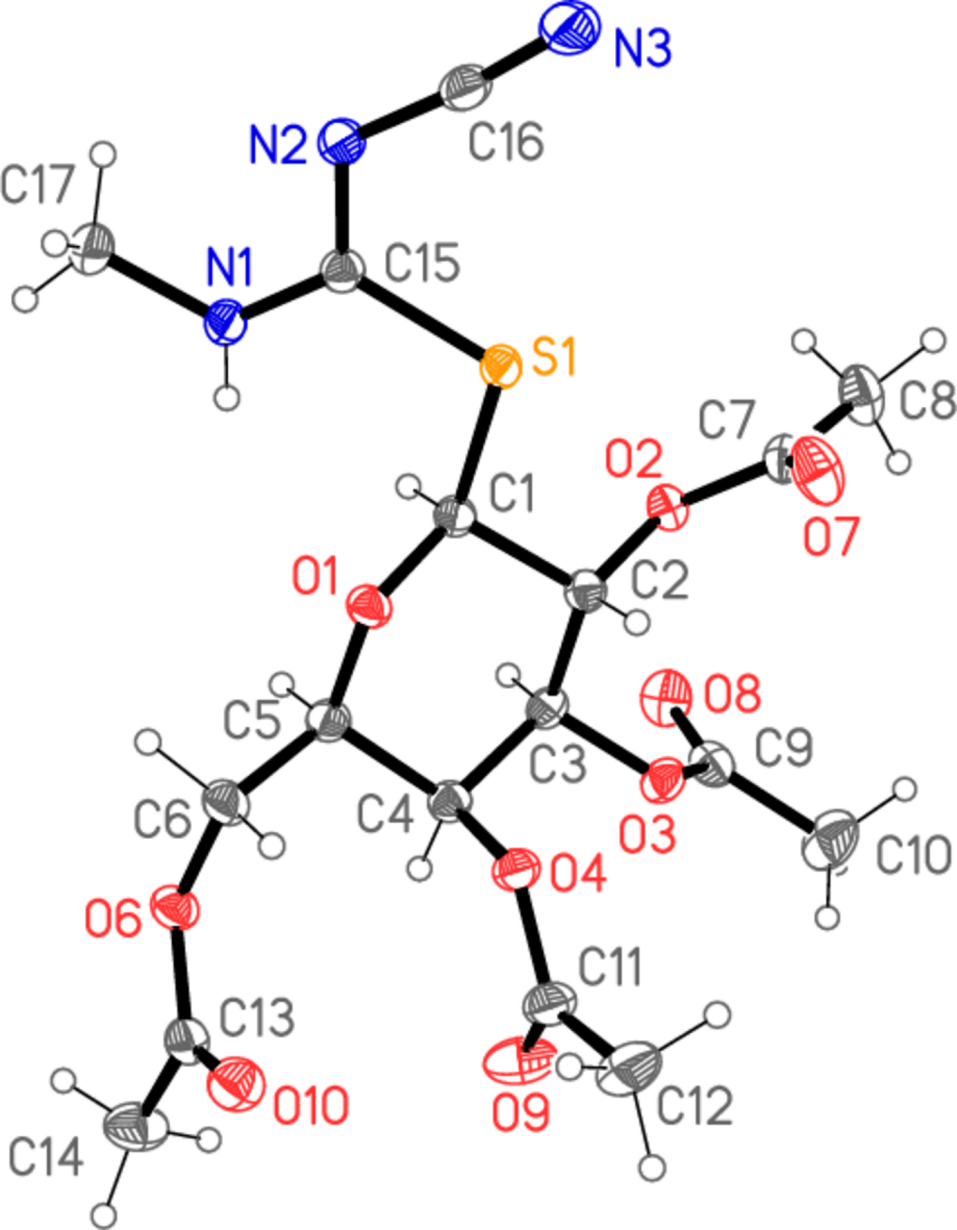
The mol­ecule of compound **5c** in the crystal. Ellipsoids represent 50% probability levels. The intra­molecular contact H01⋯O1, not drawn explicitly, is the weaker branch of a three-centre hydrogen bond (see *Supra­molecular features*).

**Figure 5:**
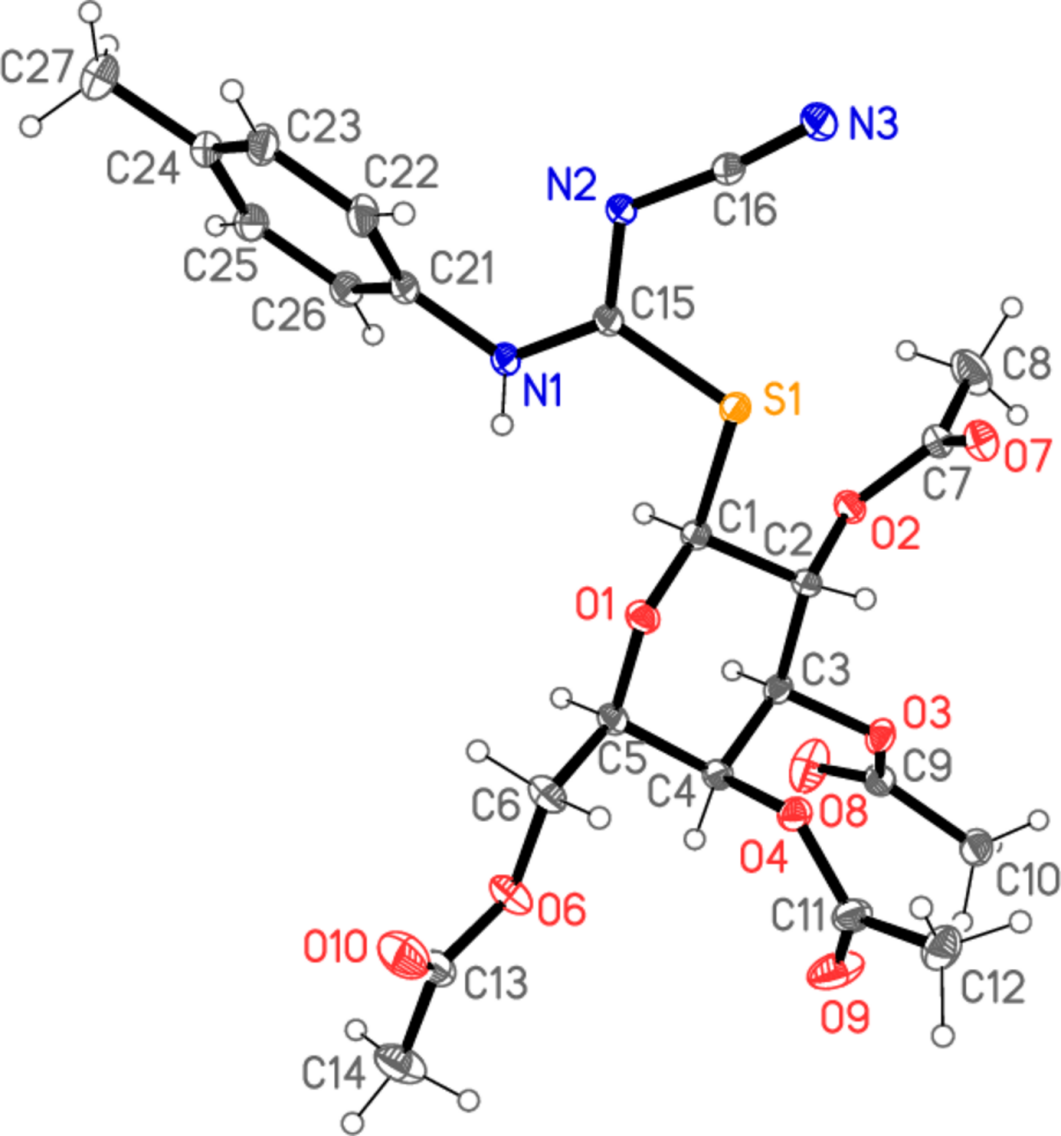
The mol­ecule of compound **5d** in the crystal. Ellipsoids represent 50% probability levels.

**Figure 12:**
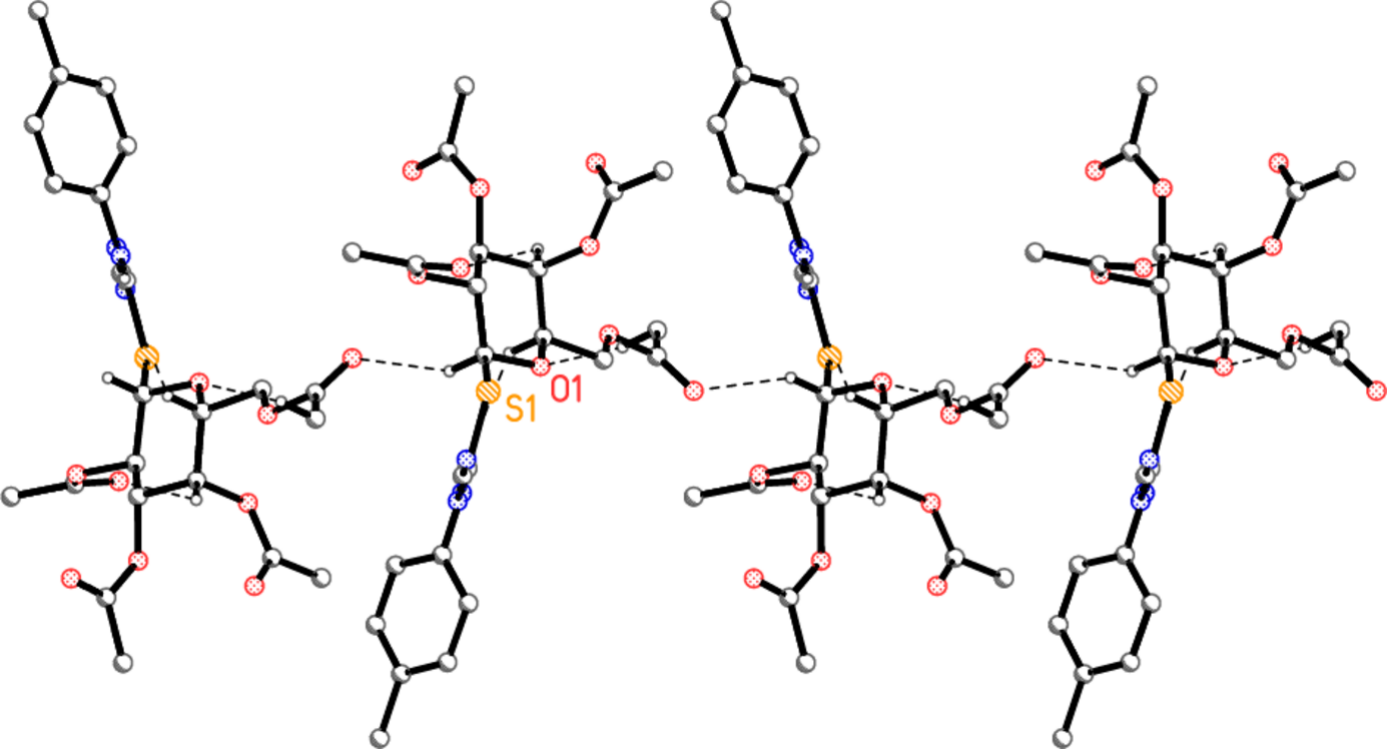
The layer from Fig. 11 ▸ is shown here in projection parallel to the *a* axis.

**Table 1:** Selected geometric parameters (Å, °) for **5a**[Chem scheme1]

C1—S1	1.8148 (6)	C15—S1	1.7759 (6)
C15—N2	1.3117 (7)	C16—N2	1.3260 (8)
C15—N1	1.3365 (7)	C21—N1	1.4187 (8)
			
C15—S1—C1	100.24 (3)		
			
C2—C3—O3—C9	−97.88 (7)	O9—C11—O4—C4	10.40 (10)
C3—O3—C9—O8	8.68 (12)	C3—C4—O4—C11	102.50 (6)
C4—C5—C6—O6	45.31 (7)	O10—C13—O6—C6	−3.73 (11)
N1—C15—S1—C1	57.56 (5)	C5—C6—O6—C13	178.14 (6)
O1—C1—S1—C15	−101.53 (4)	S1—C15—N1—C21	−170.91 (5)
S1—C1—O1—C5	179.34 (4)	C22—C21—N1—C15	16.38 (10)
O7—C7—O2—C2	2.09 (14)	S1—C15—N2—C16	8.22 (9)
C1—C2—O2—C7	135.56 (7)		

**Table 2:** Selected geometric parameters (Å, °) for **5b**[Chem scheme1]

C1—S1	1.8117 (7)	C15—S1	1.7613 (8)
C15—N2	1.3084 (10)	C16—N2	1.3203 (11)
C15—N1	1.3382 (10)	C21—N1	1.4250 (10)
			
C15—S1—C1	104.12 (3)		
			
C4—C5—C6—O6	−167.08 (6)	O9—C11—O4—C4	2.34 (14)
N1—C15—S1—C1	16.99 (8)	C3—C4—O4—C11	−108.49 (8)
O1—C1—S1—C15	−66.61 (5)	O10—C13—O6—C6	7.97 (12)
S1—C1—O1—C5	−178.96 (5)	C5—C6—O6—C13	151.83 (7)
O7—C7—O2—C2	−3.38 (14)	S1—C15—N1—C21	−174.06 (7)
C1—C2—O2—C7	126.34 (7)	C22—C21—N1—C15	46.92 (12)
O8—C9—O3—C3	1.08 (16)	S1—C15—N2—C16	5.47 (12)
C2—C3—O3—C9	−137.99 (8)		

**Table 3:** Selected geometric parameters (Å, °) for **5c**[Chem scheme1]

C1—S1	1.8093 (16)	C15—S1	1.7628 (17)
C15—N1	1.316 (2)	C16—N2	1.322 (2)
C15—N2	1.323 (2)	C17—N1	1.458 (2)
			
C15—S1—C1	103.86 (8)		
			
C4—C5—C6—O6	−63.41 (17)	O8—C9—O3—C3	−1.3 (3)
N1—C15—S1—C1	6.76 (16)	C2—C3—O3—C9	−103.98 (17)
N2—C15—S1—C1	−176.51 (13)	O9—C11—O4—C4	−2.9 (3)
O1—C1—S1—C15	−70.91 (12)	C3—C4—O4—C11	−106.48 (16)
S1—C1—O1—C5	178.36 (10)	O10—C13—O6—C6	1.7 (3)
O7—C7—O2—C2	−7.4 (2)	C5—C6—O6—C13	123.04 (16)
C1—C2—O2—C7	108.23 (15)	S1—C15—N2—C16	2.4 (2)

**Table 4:** Selected geometric parameters (Å, °) for **5d**[Chem scheme1]

C1—S1	1.8090 (11)	C15—S1	1.7593 (12)
C15—N2	1.3190 (15)	C16—N2	1.3188 (16)
C15—N1	1.3311 (15)	C21—N1	1.4388 (16)
			
C15—S1—C1	106.62 (5)		
			
C4—C5—C6—O6	−61.75 (13)	O9—C11—O4—C4	−3.0 (2)
N1—C15—S1—C1	10.44 (12)	C3—C4—O4—C11	−97.88 (12)
O1—C1—S1—C15	−83.66 (8)	O10—C13—O6—C6	−2.8 (2)
S1—C1—O1—C5	−178.19 (7)	C5—C6—O6—C13	−172.45 (11)
O7—C7—O2—C2	−1.84 (18)	S1—C15—N1—C21	−176.54 (9)
C1—C2—O2—C7	100.94 (12)	C22—C21—N1—C15	−50.03 (18)
O8—C9—O3—C3	12.60 (19)	S1—C15—N2—C16	0.87 (17)
C2—C3—O3—C9	−142.63 (10)		

**Table 5:** Hydrogen-bond geometry (Å, °) for **5a**[Chem scheme1]

*D*—H⋯*A*	*D*—H	H⋯*A*	*D*⋯*A*	*D*—H⋯*A*
N1—H01⋯N3^i^	0.845 (15)	2.118 (16)	2.9367 (8)	163.2 (15)
C1—H1⋯O10^ii^	1.00	2.31	3.3110 (8)	177
C3—H3⋯S1^iii^	1.00	2.93	3.8268 (6)	149
C10—H10*A*⋯O7^iv^	0.98	2.66	3.4036 (12)	133
C22—H22⋯N2	0.95	2.34	2.8979 (9)	117
C26—H26⋯O8^v^	0.95	2.54	3.2616 (10)	133

Symmetry codes: (i) 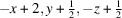; (ii) 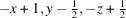; (iii) 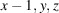; (iv) 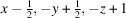; (v) 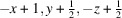.

**Table 6:** Hydrogen-bond geometry (Å, °) for **5b**[Chem scheme1]

*D*—H⋯*A*	*D*—H	H⋯*A*	*D*⋯*A*	*D*—H⋯*A*
N1—H01⋯O1	0.891 (19)	2.112 (19)	2.8910 (9)	145.5 (17)
C3—H3⋯N3^i^	1.00	2.54	3.4648 (12)	153
C6—H6*B*⋯O8^ii^	0.99	2.62	3.5823 (12)	164
C10—H10*B*⋯O6^iii^	0.98	2.59	3.5652 (14)	172
C10—H10*C*⋯O7^iv^	0.98	2.56	3.4276 (17)	148
C23—H23⋯N3^v^	0.95	2.51	3.2506 (13)	134
C25—H25⋯S1^vi^	0.95	2.90	3.7968 (10)	157
N1—H01⋯O10^vii^	0.891 (19)	2.675 (19)	2.9710 (11)	100.6 (13)

Symmetry codes: (i) 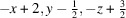; (ii) 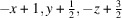; (iii) 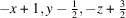; (iv) 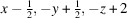; (v) 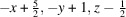; (vi) 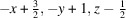; (vii) 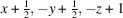.

**Table 7:** Hydrogen-bond geometry (Å, °) for **5c**[Chem scheme1]

*D*—H⋯*A*	*D*—H	H⋯*A*	*D*⋯*A*	*D*—H⋯*A*
N1—H01⋯O1	0.86 (3)	2.52 (3)	3.0637 (19)	122 (2)
N1—H01⋯N3^i^	0.86 (3)	2.14 (3)	2.880 (2)	144 (2)
C4—H4⋯O7^i^	1.00	2.41	3.225 (2)	138
C5—H5⋯N3^i^	1.00	2.47	3.258 (2)	136
C1—H1⋯O10^ii^	1.00	2.62	3.355 (2)	130
C8—H8*C*⋯O6^ii^	0.98	2.54	3.436 (2)	153
C8—H8*B*⋯O8^iii^	0.98	2.45	3.386 (3)	159

Symmetry codes: (i) 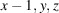; (ii) 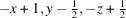; (iii) 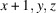.

**Table 8:** Hydrogen-bond geometry (Å, °) for **5d**[Chem scheme1]

*D*—H⋯*A*	*D*—H	H⋯*A*	*D*⋯*A*	*D*—H⋯*A*
N1—H01⋯N3^i^	0.87 (2)	2.15 (2)	2.9259 (16)	150 (2)
C14—H14*A*⋯O1^i^	0.98	2.47	3.4320 (18)	165
C4—H4⋯O7^i^	1.00	2.51	3.1985 (15)	126
C5—H5⋯N3^i^	1.00	2.52	3.2414 (17)	129
C1—H1⋯O10^ii^	1.00	2.37	3.2765 (16)	151
C25—H25⋯O9^ii^	0.95	2.59	3.4223 (19)	147
C10—H10*A*⋯O9^iii^	0.98	2.45	3.1038 (19)	124
C10—H10*C*⋯O3^iv^	0.98	2.63	3.4217 (17)	138
C12—H12*A*⋯O8^iii^	0.98	2.53	3.4790 (19)	164

Symmetry codes: (i) 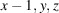; (ii) 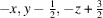; (iii) 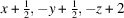; (iv) 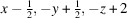.

**Table 9:** Experimental details

	**5a**	**5b**	**5c**	**5d**	**5dCu**
Crystal data					
Chemical formula	C_22_H_25_N_3_O_9_S	C_22_H_25_N_3_O_9_S	C_17_H_23_N_3_O_9_S	C_23_H_27_N_3_O_9_S	C_23_H_27_N_3_O_9_S
*M*_r_	507.51	507.51	445.44	521.53	521.53
Crystal system, space group	Orthorhombic, *P*2_1_2_1_2_1_	Orthorhombic, *P*2_1_2_1_2_1_	Orthorhombic, *P*2_1_2_1_2_1_	Orthorhombic, *P*2_1_2_1_2_1_	Orthorhombic, *P*2_1_2_1_2_1_
Temperature (K)	100	100	100	100	100
*a*, *b*, *c* (Å)	7.11266 (11), 13.76500 (18), 24.3850 (3)	10.10503 (14), 14.5978 (2), 16.4256 (2)	7.34295 (7), 13.92258 (13), 21.07464 (18)	7.42044 (17), 14.9634 (4), 22.7580 (5)	7.41967 (5), 14.96519 (12), 22.75622 (16)
*V* (Å^3^)	2387.44 (6)	2422.96 (6)	2154.52 (3)	2526.94 (11)	2526.78 (3)
*Z*	4	4	4	4	4
Radiation type	Mo *K*α	Mo *K*α	Cu *K*α	Mo *K*α	Cu *K*α
μ (mm^−1^)	0.19	0.19	1.81	0.18	1.63
Crystal size (mm)	0.22 × 0.20 × 0.12	0.3 × 0.2 × 0.2	0.18 × 0.08 × 0.06	0.15 × 0.05 × 0.04	0.15 × 0.05 × 0.04

Data collection					
Diffractometer	XtaLAB Synergy	XtaLAB Synergy	XtaLAB Synergy	XtaLAB Synergy	XtaLAB Synergy
Absorption correction	Multi-scan (*CrysAlis PRO*; Rigaku OD, 2023 ▸)	Multi-scan (*CrysAlis PRO*; Rigaku OD, 2023 ▸)	Multi-scan (*CrysAlis PRO*; Rigaku OD, 2023 ▸)	Multi-scan (*CrysAlis PRO*; Rigaku OD, 2023 ▸)	Multi-scan (*CrysAlis PRO*; Rigaku OD, 2023 ▸)
*T*_min_, *T*_max_	0.873, 1.000	0.741, 1.000	0.694, 1.000	0.881, 1.000	0.781, 1.000
No. of measured, independent and observed [*I* > 2σ(*I*)] reflections	316930, 19593, 17947	308676, 16031, 14496	101235, 4696, 4656	146926, 12241, 11008	122360, 5508, 5458
*R*_int_	0.074	0.071	0.047	0.085	0.033
θ values (°)	θ_max_ = 44.9, θ_min_ = 2.2	θ_max_ = 41.4, θ_min_ = 2.4	θ_max_ = 80.4, θ_min_ = 3.8	θ_max_ = 36.3, θ_min_ = 2.3	θ_max_ = 80.6, θ_min_ = 3.5
(sin θ/λ)_max_ (Å^−1^)	0.993	0.930	0.639	0.833	0.640

Refinement					
*R*[*F*^2^ > 2σ(*F*^2^)], *wR*(*F*^2^), *S*	0.029, 0.079, 1.06	0.031, 0.084, 1.06	0.024, 0.065, 1.05	0.036, 0.094, 1.04	0.023, 0.059, 1.04
No. of reflections	19593	16031	4696	12241	5508
No. of parameters	338	324	280	334	334
No. of restraints	6	0	0	0	0
H-atom treatment	H atoms treated by a mixture of independent and constrained refinement	H atoms treated by a mixture of independent and constrained refinement	H atoms treated by a mixture of independent and constrained refinement	H atoms treated by a mixture of independent and constrained refinement	H atoms treated by a mixture of independent and constrained refinement
Δρ_max_, Δρ_min_ (e Å^−3^)	0.48, −0.21	0.45, −0.18	0.21, −0.24	0.45, −0.21	0.22, −0.18
Absolute structure	Flack *x* determined using 7711 quotients [(*I*^+^)−(*I*^−^)]/[(*I*^+^)+(*I*^−^)] (Parsons *et al.*, 2013 ▸)	Flack *x* determined using 6240 quotients [(*I*^+^)−(*I*^−^)]/[(*I*^+^)+(*I*^−^)] (Parsons *et al.*, 2013 ▸)	Flack *x* determined using 1977 quotients [(*I*^+^)−(*I*^−^)]/[(*I*^+^)+(*I*^−^)] (Parsons *et al.*, 2013 ▸)	Flack *x* determined using 4554 quotients [(*I*^+^)−(*I*^−^)]/[(*I*^+^)+(*I*^−^)] (Parsons *et al.*, 2013 ▸)	Flack *x* determined using 2334 quotients [(*I*^+^)−(*I*^−^)]/[(*I*^+^)+(*I*^−^)] (Parsons *et al.*, 2013 ▸)
Absolute structure parameter	0.000 (9)	−0.002 (10)	0.000 (4)	0.005 (18)	−0.004 (2)

Computer programs: *CrysAlis PRO* (Rigaku OD, 2023 ▸), *SHELXT* (Sheldrick, 2015*a* ▸), *SHELXL2019/3* (Sheldrick, 2015*b* ▸) and *XP*, (Bruker, 1998 ▸).

## supplementary crystallographic information

### 2',3',4',6'-Tetra-O-acetyl-β-D-glucopyranosyl N'-cyano-N-phenylcarbamimidothioate (5a) Crystal data

**Table:**

C_22_H_25_N_3_O_9_S	*D*_x_ = 1.412 Mg m^−^^3^
*M**_r_* = 507.51	Mo *K*α radiation, λ = 0.71073 Å
Orthorhombic, *P*2_1_2_1_2_1_	Cell parameters from 133018 reflections
*a* = 7.11266 (11) Å	θ = 2.2–45.1°
*b* = 13.76500 (18) Å	µ = 0.19 mm^−^^1^
*c* = 24.3850 (3) Å	*T* = 100 K
*V* = 2387.44 (6) Å^3^	Tablet, colourless
*Z* = 4	0.22 × 0.20 × 0.12 mm
*F*(000) = 1064	

### 2',3',4',6'-Tetra-O-acetyl-β-D-glucopyranosyl N'-cyano-N-phenylcarbamimidothioate (5a) Data collection

**Table:**

XtaLAB Synergy diffractometer	19593 independent reflections
Radiation source: micro-focus sealed X-ray tube, PhotonJet (Mo) X-ray Source	17947 reflections with *I* > 2σ(*I*)
Mirror monochromator	*R*_int_ = 0.074
Detector resolution: 10.0000 pixels mm^-1^	θ_max_ = 44.9°, θ_min_ = 2.2°
ω scans	*h* = −14→14
Absorption correction: multi-scan (CrysAlisPro; Rigaku OD, 2023)	*k* = −27→27
*T*_min_ = 0.873, *T*_max_ = 1.000	*l* = −48→48
316930 measured reflections	

### 2',3',4',6'-Tetra-O-acetyl-β-D-glucopyranosyl N'-cyano-N-phenylcarbamimidothioate (5a) Refinement

**Table:**

Refinement on *F*^2^	Hydrogen site location: mixed
Least-squares matrix: full	H atoms treated by a mixture of independent and constrained refinement
*R*[*F*^2^ > 2σ(*F*^2^)] = 0.029	*w* = 1/[σ^2^(*F*_o_^2^) + (0.048*P*)^2^ + 0.0766*P*] where *P* = (*F*_o_^2^ + 2*F*_c_^2^)/3
*wR*(*F*^2^) = 0.079	(Δ/σ)_max_ = 0.001
*S* = 1.06	Δρ_max_ = 0.48 e Å^−^^3^
19593 reflections	Δρ_min_ = −0.21 e Å^−^^3^
338 parameters	Absolute structure: Flack *x* determined using 7711 quotients [(*I*^+^)-(*I*^-^)]/[(*I*^+^)+(*I*^-^)] (Parsons *et al.*, 2013)
6 restraints	Absolute structure parameter: 0.000 (9)
Primary atom site location: dual	

### 2',3',4',6'-Tetra-O-acetyl-β-D-glucopyranosyl N'-cyano-N-phenylcarbamimidothioate (5a) Fractional atomic coordinates and isotropic or equivalent isotropic displacement parameters (Å^2^)

**Table:**

	*x*	*y*	*z*	*U*_iso_*/*U*_eq_	Occ. (<1)
C1	0.67902 (9)	0.49177 (4)	0.30041 (2)	0.01352 (8)	
H1	0.607670	0.469895	0.267301	0.016*	
C2	0.59604 (9)	0.44485 (4)	0.35209 (2)	0.01403 (8)	
H2	0.681305	0.455499	0.384141	0.017*	
C3	0.40161 (9)	0.48626 (4)	0.36397 (2)	0.01407 (8)	
H3	0.309321	0.461262	0.336444	0.017*	
O3	0.34163 (8)	0.46062 (4)	0.41825 (2)	0.01747 (8)	
O8	0.18714 (14)	0.32864 (5)	0.38655 (3)	0.02920 (16)	0.915 (2)
C9	0.22538 (12)	0.38311 (5)	0.42355 (3)	0.01913 (13)	0.915 (2)
C10	0.15689 (18)	0.37581 (8)	0.48172 (5)	0.0293 (2)	0.915 (2)
H10A	0.243788	0.335424	0.503006	0.044*	0.915 (2)
H10B	0.031472	0.346335	0.482175	0.044*	0.915 (2)
H10C	0.150750	0.440894	0.497907	0.044*	0.915 (2)
O8'	0.0406 (10)	0.4496 (6)	0.4126 (3)	0.0240 (13)*	0.085 (2)
C9'	0.1765 (11)	0.4345 (6)	0.4407 (3)	0.0173 (12)*	0.085 (2)
C10'	0.1820 (15)	0.3951 (8)	0.4982 (4)	0.0233 (17)*	0.085 (2)
H10D	0.289209	0.423227	0.517864	0.035*	0.085 (2)
H10E	0.195208	0.324269	0.497011	0.035*	0.085 (2)
H10F	0.065248	0.412132	0.517263	0.035*	0.085 (2)
C4	0.40567 (8)	0.59702 (4)	0.36240 (2)	0.01314 (7)	
H4	0.484944	0.622506	0.393062	0.016*	
C5	0.48523 (9)	0.63045 (4)	0.30735 (2)	0.01386 (8)	
H5	0.409177	0.601467	0.276990	0.017*	
C6	0.49213 (9)	0.73964 (4)	0.30034 (3)	0.01568 (9)	
H6A	0.573273	0.756840	0.268815	0.019*	
H6B	0.364335	0.765314	0.293363	0.019*	
C7	0.63786 (13)	0.27985 (5)	0.37910 (3)	0.02216 (13)	
C8	0.62011 (18)	0.17779 (6)	0.35875 (4)	0.0324 (2)	
H8A	0.493557	0.167691	0.343918	0.049*	
H8B	0.641808	0.132528	0.389119	0.049*	
H8C	0.713332	0.166268	0.329882	0.049*	
C11	0.16447 (10)	0.66570 (5)	0.41830 (3)	0.01716 (9)	
C12	−0.04426 (12)	0.68034 (7)	0.42059 (4)	0.02455 (13)	
H11A	−0.087665	0.708723	0.385980	0.037*	
H11B	−0.075191	0.724242	0.450918	0.037*	
H11C	−0.106371	0.617621	0.426385	0.037*	
C13	0.58837 (10)	0.87774 (4)	0.35053 (3)	0.01704 (9)	
C14	0.65175 (12)	0.91368 (5)	0.40561 (3)	0.02219 (12)	
H14A	0.664811	0.858562	0.430759	0.033*	
H14B	0.558608	0.959336	0.420204	0.033*	
H14C	0.773211	0.946622	0.401839	0.033*	
C15	0.93811 (8)	0.42870 (4)	0.22430 (2)	0.01206 (7)	
C16	1.00819 (10)	0.26983 (4)	0.23998 (3)	0.01645 (9)	
C21	0.87594 (8)	0.50362 (4)	0.13364 (2)	0.01345 (8)	
C22	0.84654 (10)	0.41959 (5)	0.10275 (3)	0.01692 (9)	
H22	0.840788	0.357998	0.120248	0.020*	
C23	0.82570 (12)	0.42666 (6)	0.04608 (3)	0.02085 (11)	
H23	0.807747	0.369485	0.024898	0.025*	
C24	0.83094 (14)	0.51651 (7)	0.02030 (3)	0.02580 (14)	
H24	0.817216	0.520920	−0.018375	0.031*	
C25	0.85640 (16)	0.59990 (6)	0.05146 (3)	0.02884 (16)	
H25	0.858150	0.661515	0.033920	0.035*	
C26	0.87938 (13)	0.59439 (5)	0.10806 (3)	0.02130 (11)	
H26	0.897212	0.651772	0.129069	0.026*	
S1	0.92163 (2)	0.45177 (2)	0.29580 (2)	0.01623 (3)	
O1	0.67426 (7)	0.59454 (3)	0.30422 (2)	0.01441 (7)	
O2	0.58121 (9)	0.34328 (3)	0.34013 (2)	0.01906 (8)	
O4	0.21602 (7)	0.63100 (4)	0.36817 (2)	0.01543 (7)	
O6	0.56703 (8)	0.78073 (3)	0.35006 (2)	0.01623 (7)	
O7	0.69457 (18)	0.30441 (5)	0.42334 (3)	0.0405 (2)	
O9	0.27345 (10)	0.68139 (5)	0.45493 (3)	0.02604 (11)	
O10	0.55671 (11)	0.92843 (4)	0.31108 (3)	0.02551 (11)	
N1	0.89858 (8)	0.50367 (3)	0.19144 (2)	0.01386 (7)	
H01	0.898 (2)	0.5583 (11)	0.2072 (6)	0.028 (4)*	
N2	0.98973 (8)	0.34357 (4)	0.20540 (2)	0.01468 (7)	
N3	1.02603 (12)	0.19911 (5)	0.26556 (3)	0.02431 (12)	

### 2',3',4',6'-Tetra-O-acetyl-β-D-glucopyranosyl N'-cyano-N-phenylcarbamimidothioate (5a) Atomic displacement parameters (Å^2^)

**Table:**

	*U*^11^	*U*^22^	*U*^33^	*U*^12^	*U*^13^	*U*^23^
C1	0.0178 (2)	0.01183 (17)	0.01096 (17)	0.00272 (15)	−0.00130 (15)	−0.00107 (14)
C2	0.0204 (2)	0.00989 (16)	0.01183 (17)	0.00177 (16)	−0.00138 (16)	−0.00075 (13)
C3	0.0183 (2)	0.01066 (17)	0.01323 (18)	−0.00019 (15)	−0.00076 (16)	−0.00004 (14)
O3	0.0228 (2)	0.01426 (16)	0.01539 (16)	−0.00305 (15)	0.00414 (15)	−0.00120 (13)
O8	0.0446 (4)	0.0185 (2)	0.0244 (3)	−0.0130 (3)	−0.0016 (3)	0.0010 (2)
C9	0.0250 (3)	0.0126 (2)	0.0198 (3)	−0.0017 (2)	0.0019 (2)	0.00355 (19)
C10	0.0409 (5)	0.0214 (3)	0.0256 (4)	−0.0045 (3)	0.0118 (4)	0.0057 (3)
C4	0.01485 (19)	0.01058 (16)	0.01398 (18)	0.00118 (14)	−0.00102 (15)	−0.00005 (14)
C5	0.0158 (2)	0.01188 (17)	0.01390 (18)	0.00211 (15)	−0.00130 (16)	0.00081 (14)
C6	0.0188 (2)	0.01234 (18)	0.0159 (2)	0.00268 (16)	−0.00159 (18)	0.00251 (15)
C7	0.0364 (4)	0.0122 (2)	0.0179 (2)	0.0047 (2)	0.0072 (2)	0.00379 (17)
C8	0.0528 (6)	0.0112 (2)	0.0332 (4)	0.0038 (3)	0.0116 (4)	0.0020 (2)
C11	0.0204 (2)	0.0134 (2)	0.0176 (2)	0.00282 (18)	0.00212 (19)	0.00011 (16)
C12	0.0197 (3)	0.0261 (3)	0.0278 (3)	0.0027 (2)	0.0066 (2)	0.0009 (3)
C13	0.0180 (2)	0.01118 (18)	0.0219 (2)	0.00189 (16)	−0.00122 (19)	0.00177 (16)
C14	0.0245 (3)	0.0162 (2)	0.0259 (3)	0.0002 (2)	−0.0045 (2)	−0.0019 (2)
C15	0.01312 (18)	0.00960 (15)	0.01346 (17)	0.00101 (13)	−0.00051 (14)	−0.00093 (13)
C16	0.0212 (2)	0.01096 (18)	0.0171 (2)	0.00392 (17)	0.00095 (19)	0.00014 (15)
C21	0.01434 (19)	0.01211 (17)	0.01390 (18)	0.00085 (14)	0.00055 (15)	0.00071 (14)
C22	0.0216 (2)	0.0149 (2)	0.0143 (2)	−0.00132 (18)	−0.00154 (18)	−0.00077 (16)
C23	0.0257 (3)	0.0224 (3)	0.0144 (2)	−0.0003 (2)	−0.0011 (2)	−0.00121 (19)
C24	0.0355 (4)	0.0277 (3)	0.0142 (2)	0.0006 (3)	0.0007 (2)	0.0038 (2)
C25	0.0463 (5)	0.0212 (3)	0.0190 (3)	0.0003 (3)	0.0002 (3)	0.0072 (2)
C26	0.0317 (3)	0.0137 (2)	0.0184 (2)	0.0010 (2)	0.0007 (2)	0.00354 (18)
S1	0.01891 (6)	0.01705 (6)	0.01272 (5)	0.00635 (5)	−0.00441 (4)	−0.00329 (4)
O1	0.01621 (16)	0.01094 (14)	0.01608 (16)	0.00274 (12)	0.00068 (13)	0.00052 (12)
O2	0.0298 (2)	0.00939 (14)	0.01796 (17)	0.00238 (15)	−0.00270 (18)	−0.00127 (13)
O4	0.01475 (16)	0.01583 (16)	0.01572 (17)	0.00233 (13)	−0.00017 (13)	−0.00029 (13)
O6	0.02063 (19)	0.01058 (14)	0.01749 (17)	0.00061 (13)	−0.00200 (15)	0.00184 (12)
O7	0.0826 (7)	0.0213 (2)	0.0174 (2)	0.0096 (3)	−0.0058 (3)	0.00543 (19)
O9	0.0288 (3)	0.0292 (3)	0.0201 (2)	0.0075 (2)	−0.0032 (2)	−0.0089 (2)
O10	0.0360 (3)	0.01366 (17)	0.0269 (2)	0.00210 (19)	−0.0068 (2)	0.00636 (17)
N1	0.01859 (19)	0.00905 (14)	0.01394 (16)	0.00104 (13)	−0.00146 (14)	−0.00041 (12)
N2	0.01933 (19)	0.00998 (14)	0.01473 (17)	0.00311 (14)	0.00178 (16)	−0.00016 (13)
N3	0.0364 (3)	0.0139 (2)	0.0227 (2)	0.0072 (2)	0.0012 (2)	0.00323 (18)

### 2',3',4',6'-Tetra-O-acetyl-β-D-glucopyranosyl N'-cyano-N-phenylcarbamimidothioate (5a) Geometric parameters (Å, º)

**Table:**

C1—O1	1.4180 (7)	C8—H8A	0.9800
C1—C2	1.5343 (8)	C8—H8B	0.9800
C1—S1	1.8148 (6)	C8—H8C	0.9800
C1—H1	1.0000	C11—O9	1.2022 (10)
C2—O2	1.4321 (7)	C11—O4	1.3627 (8)
C2—C3	1.5236 (9)	C11—C12	1.4993 (11)
C2—H2	1.0000	C12—H11A	0.9800
C3—O3	1.4347 (8)	C12—H11B	0.9800
C3—C4	1.5254 (8)	C12—H11C	0.9800
C3—H3	1.0000	C13—O10	1.2094 (9)
O3—C9'	1.345 (8)	C13—O6	1.3440 (8)
O3—C9	1.3560 (9)	C13—C14	1.5007 (11)
O8—C9	1.2042 (11)	C14—H14A	0.9800
C9—C10	1.5033 (12)	C14—H14B	0.9800
C10—H10A	0.9800	C14—H14C	0.9800
C10—H10B	0.9800	C15—N2	1.3117 (7)
C10—H10C	0.9800	C15—N1	1.3365 (7)
O8'—C9'	1.204 (10)	C15—S1	1.7759 (6)
C9'—C10'	1.504 (12)	C16—N3	1.1632 (9)
C10'—H10D	0.9800	C16—N2	1.3260 (8)
C10'—H10E	0.9800	C21—C22	1.3962 (9)
C10'—H10F	0.9800	C21—C26	1.3967 (9)
C4—O4	1.4346 (8)	C21—N1	1.4187 (8)
C4—C5	1.5276 (8)	C22—C23	1.3932 (9)
C4—H4	1.0000	C22—H22	0.9500
C5—O1	1.4345 (8)	C23—C24	1.3879 (11)
C5—C6	1.5135 (8)	C23—H23	0.9500
C5—H5	1.0000	C24—C25	1.3884 (13)
C6—O6	1.4400 (8)	C24—H24	0.9500
C6—H6A	0.9900	C25—C26	1.3919 (11)
C6—H6B	0.9900	C25—H25	0.9500
C7—O7	1.2004 (11)	C26—H26	0.9500
C7—O2	1.3519 (9)	N1—H01	0.845 (15)
C7—C8	1.4952 (11)		
			
O1—C1—C2	110.91 (5)	O7—C7—C8	126.24 (7)
O1—C1—S1	109.24 (4)	O2—C7—C8	110.39 (7)
C2—C1—S1	106.79 (4)	C7—C8—H8A	109.5
O1—C1—H1	109.9	C7—C8—H8B	109.5
C2—C1—H1	109.9	H8A—C8—H8B	109.5
S1—C1—H1	109.9	C7—C8—H8C	109.5
O2—C2—C3	109.70 (5)	H8A—C8—H8C	109.5
O2—C2—C1	105.78 (5)	H8B—C8—H8C	109.5
C3—C2—C1	110.36 (5)	O9—C11—O4	123.78 (7)
O2—C2—H2	110.3	O9—C11—C12	125.91 (7)
C3—C2—H2	110.3	O4—C11—C12	110.31 (7)
C1—C2—H2	110.3	C11—C12—H11A	109.5
O3—C3—C2	110.69 (5)	C11—C12—H11B	109.5
O3—C3—C4	105.95 (5)	H11A—C12—H11B	109.5
C2—C3—C4	110.61 (5)	C11—C12—H11C	109.5
O3—C3—H3	109.8	H11A—C12—H11C	109.5
C2—C3—H3	109.8	H11B—C12—H11C	109.5
C4—C3—H3	109.8	O10—C13—O6	123.05 (7)
C9'—O3—C3	134.5 (3)	O10—C13—C14	125.28 (6)
C9—O3—C3	117.56 (5)	O6—C13—C14	111.66 (6)
O8—C9—O3	123.80 (7)	C13—C14—H14A	109.5
O8—C9—C10	126.31 (8)	C13—C14—H14B	109.5
O3—C9—C10	109.88 (7)	H14A—C14—H14B	109.5
C9—C10—H10A	109.5	C13—C14—H14C	109.5
C9—C10—H10B	109.5	H14A—C14—H14C	109.5
H10A—C10—H10B	109.5	H14B—C14—H14C	109.5
C9—C10—H10C	109.5	N2—C15—N1	122.54 (5)
H10A—C10—H10C	109.5	N2—C15—S1	121.55 (4)
H10B—C10—H10C	109.5	N1—C15—S1	115.89 (4)
O8'—C9'—O3	115.0 (7)	N3—C16—N2	172.95 (7)
O8'—C9'—C10'	127.9 (8)	C22—C21—C26	120.18 (6)
O3—C9'—C10'	117.0 (7)	C22—C21—N1	123.61 (5)
C9'—C10'—H10D	109.5	C26—C21—N1	116.19 (5)
C9'—C10'—H10E	109.5	C23—C22—C21	119.55 (6)
H10D—C10'—H10E	109.5	C23—C22—H22	120.2
C9'—C10'—H10F	109.5	C21—C22—H22	120.2
H10D—C10'—H10F	109.5	C24—C23—C22	120.58 (7)
H10E—C10'—H10F	109.5	C24—C23—H23	119.7
O4—C4—C3	107.79 (5)	C22—C23—H23	119.7
O4—C4—C5	109.65 (5)	C23—C24—C25	119.49 (7)
C3—C4—C5	109.28 (5)	C23—C24—H24	120.3
O4—C4—H4	110.0	C25—C24—H24	120.3
C3—C4—H4	110.0	C24—C25—C26	120.86 (7)
C5—C4—H4	110.0	C24—C25—H25	119.6
O1—C5—C6	107.81 (5)	C26—C25—H25	119.6
O1—C5—C4	106.87 (5)	C25—C26—C21	119.31 (7)
C6—C5—C4	114.23 (5)	C25—C26—H26	120.3
O1—C5—H5	109.3	C21—C26—H26	120.3
C6—C5—H5	109.3	C15—S1—C1	100.24 (3)
C4—C5—H5	109.3	C1—O1—C5	111.70 (5)
O6—C6—C5	107.87 (5)	C7—O2—C2	117.73 (5)
O6—C6—H6A	110.1	C11—O4—C4	117.08 (5)
C5—C6—H6A	110.1	C13—O6—C6	116.05 (5)
O6—C6—H6B	110.1	C15—N1—C21	128.26 (5)
C5—C6—H6B	110.1	C15—N1—H01	114.5 (10)
H6A—C6—H6B	108.4	C21—N1—H01	116.9 (10)
O7—C7—O2	123.37 (7)	C15—N2—C16	119.21 (6)
			
O1—C1—C2—O2	171.22 (5)	C23—C24—C25—C26	0.92 (16)
S1—C1—C2—O2	−69.85 (5)	C24—C25—C26—C21	−0.25 (15)
O1—C1—C2—C3	52.63 (6)	C22—C21—C26—C25	−1.08 (12)
S1—C1—C2—C3	171.56 (4)	N1—C21—C26—C25	−179.45 (8)
O2—C2—C3—O3	77.33 (6)	N2—C15—S1—C1	−123.75 (5)
C1—C2—C3—O3	−166.51 (5)	N1—C15—S1—C1	57.56 (5)
O2—C2—C3—C4	−165.56 (5)	O1—C1—S1—C15	−101.53 (4)
C1—C2—C3—C4	−49.39 (6)	C2—C1—S1—C15	138.46 (4)
C2—C3—O3—C9'	−141.6 (5)	C2—C1—O1—C5	−63.21 (6)
C4—C3—O3—C9'	98.5 (5)	S1—C1—O1—C5	179.34 (4)
C2—C3—O3—C9	−97.88 (7)	C6—C5—O1—C1	−169.35 (5)
C4—C3—O3—C9	142.17 (6)	C4—C5—O1—C1	67.42 (6)
C3—O3—C9—O8	8.68 (12)	O7—C7—O2—C2	2.09 (14)
C3—O3—C9—C10	−172.23 (7)	C8—C7—O2—C2	−177.59 (7)
C3—O3—C9'—O8'	−14.2 (10)	C3—C2—O2—C7	−105.42 (7)
C3—O3—C9'—C10'	168.7 (6)	C1—C2—O2—C7	135.56 (7)
O3—C3—C4—O4	−65.90 (6)	O9—C11—O4—C4	10.40 (10)
C2—C3—C4—O4	174.10 (4)	C12—C11—O4—C4	−169.32 (6)
O3—C3—C4—C5	174.99 (5)	C3—C4—O4—C11	102.50 (6)
C2—C3—C4—C5	54.99 (6)	C5—C4—O4—C11	−138.63 (5)
O4—C4—C5—O1	179.85 (4)	O10—C13—O6—C6	−3.73 (11)
C3—C4—C5—O1	−62.21 (6)	C14—C13—O6—C6	175.24 (6)
O4—C4—C5—C6	60.71 (6)	C5—C6—O6—C13	178.14 (6)
C3—C4—C5—C6	178.65 (5)	N2—C15—N1—C21	10.42 (10)
O1—C5—C6—O6	−73.30 (6)	S1—C15—N1—C21	−170.91 (5)
C4—C5—C6—O6	45.31 (7)	C22—C21—N1—C15	16.38 (10)
C26—C21—C22—C23	1.71 (10)	C26—C21—N1—C15	−165.31 (7)
N1—C21—C22—C23	179.96 (6)	N1—C15—N2—C16	−173.19 (6)
C21—C22—C23—C24	−1.03 (12)	S1—C15—N2—C16	8.22 (9)
C22—C23—C24—C25	−0.28 (14)		

### 2',3',4',6'-Tetra-O-acetyl-β-D-glucopyranosyl N'-cyano-N-phenylcarbamimidothioate (5a) Hydrogen-bond geometry (Å, º)

**Table:**

*D*—H···*A*	*D*—H	H···*A*	*D*···*A*	*D*—H···*A*
N1—H01···N3^i^	0.845 (15)	2.118 (16)	2.9367 (8)	163.2 (15)
C1—H1···O10^ii^	1.00	2.31	3.3110 (8)	177
C3—H3···S1^iii^	1.00	2.93	3.8268 (6)	149
C10—H10*A*···O7^iv^	0.98	2.66	3.4036 (12)	133
C22—H22···N2	0.95	2.34	2.8979 (9)	117
C26—H26···O8^v^	0.95	2.54	3.2616 (10)	133

Symmetry codes: (i) −*x*+2, *y*+1/2, −*z*+1/2; (ii) −*x*+1, *y*−1/2, −*z*+1/2; (iii) *x*−1, *y*, *z*; (iv) *x*−1/2, −*y*+1/2, −*z*+1; (v) −*x*+1, *y*+1/2, −*z*+1/2.

### 2',3',4',6'-Tetra-O-acetyl-β-D-galactopyranosyl N'-cyano-N-phenylcarbamimidothioate (5b) Crystal data

**Table:**

C_22_H_25_N_3_O_9_S	*D*_x_ = 1.391 Mg m^−^^3^
*M**_r_* = 507.51	Mo *K*α radiation, λ = 0.71073 Å
Orthorhombic, *P*2_1_2_1_2_1_	Cell parameters from 135804 reflections
*a* = 10.10503 (14) Å	θ = 2.4–41.4°
*b* = 14.5978 (2) Å	µ = 0.19 mm^−^^1^
*c* = 16.4256 (2) Å	*T* = 100 K
*V* = 2422.96 (6) Å^3^	Tetrahedron, colourless
*Z* = 4	0.3 × 0.2 × 0.2 mm
*F*(000) = 1064	

### 2',3',4',6'-Tetra-O-acetyl-β-D-galactopyranosyl N'-cyano-N-phenylcarbamimidothioate (5b) Data collection

**Table:**

XtaLAB Synergy diffractometer	16031 independent reflections
Radiation source: micro-focus sealed X-ray tube, PhotonJet (Mo) X-ray Source	14496 reflections with *I* > 2σ(*I*)
Mirror monochromator	*R*_int_ = 0.071
Detector resolution: 10.0000 pixels mm^-1^	θ_max_ = 41.4°, θ_min_ = 2.4°
ω scans	*h* = −18→18
Absorption correction: multi-scan (CrysAlisPro; Rigaku OD, 2023)	*k* = −26→26
*T*_min_ = 0.741, *T*_max_ = 1.000	*l* = −30→30
308676 measured reflections	

### 2',3',4',6'-Tetra-O-acetyl-β-D-galactopyranosyl N'-cyano-N-phenylcarbamimidothioate (5b) Refinement

**Table:**

Refinement on *F*^2^	Hydrogen site location: mixed
Least-squares matrix: full	H atoms treated by a mixture of independent and constrained refinement
*R*[*F*^2^ > 2σ(*F*^2^)] = 0.031	*w* = 1/[σ^2^(*F*_o_^2^) + (0.0544*P*)^2^ + 0.0728*P*] where *P* = (*F*_o_^2^ + 2*F*_c_^2^)/3
*wR*(*F*^2^) = 0.084	(Δ/σ)_max_ = 0.013
*S* = 1.06	Δρ_max_ = 0.45 e Å^−^^3^
16031 reflections	Δρ_min_ = −0.18 e Å^−^^3^
324 parameters	Absolute structure: Flack *x* determined using 6240 quotients [(*I*^+^)-(*I*^-^)]/[(*I*^+^)+(*I*^-^)] (Parsons *et al.*, 2013)
0 restraints	Absolute structure parameter: −0.002 (10)
Primary atom site location: dual	

### 2',3',4',6'-Tetra-O-acetyl-β-D-galactopyranosyl N'-cyano-N-phenylcarbamimidothioate (5b) Special details

**Table:**

Geometry. The symmetry employed for this shelxl refinement is uniquely defined by the following loop, which should always be used as a source of symmetry information in preference to the above space-group names. They are only intended as comments.
Refinement. The symmetry employed for this shelxl refinement is uniquely defined by the following loop, which should always be used as a source of symmetry information in preference to the above space-group names. They are only intended as comments.

### 2',3',4',6'-Tetra-O-acetyl-β-D-galactopyranosyl N'-cyano-N-phenylcarbamimidothioate (5b) Fractional atomic coordinates and isotropic or equivalent isotropic displacement parameters (Å^2^)

**Table:**

	*x*	*y*	*z*	*U*_iso_*/*U*_eq_	
C1	0.78000 (7)	0.30228 (5)	0.74073 (4)	0.01539 (10)	
H1	0.842334	0.261031	0.710862	0.018*	
C2	0.73116 (7)	0.25567 (5)	0.81904 (5)	0.01599 (10)	
H2	0.683510	0.300471	0.854564	0.019*	
C3	0.64215 (8)	0.17512 (5)	0.79800 (5)	0.01690 (11)	
H3	0.697254	0.124494	0.774907	0.020*	
C4	0.53520 (7)	0.20081 (5)	0.73704 (5)	0.01690 (11)	
H4	0.487293	0.144413	0.719005	0.020*	
C5	0.60052 (7)	0.24636 (5)	0.66399 (5)	0.01635 (10)	
H5	0.663885	0.202193	0.638582	0.020*	
C6	0.50147 (8)	0.27647 (6)	0.60069 (5)	0.01953 (12)	
H6A	0.437540	0.226701	0.589314	0.023*	
H6B	0.452020	0.330892	0.619876	0.023*	
C7	0.86533 (9)	0.24392 (6)	0.93881 (5)	0.02163 (13)	
C8	0.98311 (11)	0.19611 (8)	0.97327 (7)	0.02888 (17)	
H8A	0.953831	0.147211	1.009959	0.043*	
H8B	1.037409	0.240053	1.003579	0.043*	
H8C	1.035493	0.169657	0.928899	0.043*	
C9	0.57591 (10)	0.05350 (6)	0.88563 (6)	0.02542 (16)	
C10	0.51045 (16)	0.03493 (8)	0.96596 (9)	0.0409 (3)	
H10A	0.568115	0.055828	1.010181	0.061*	
H10B	0.494479	−0.030986	0.971610	0.061*	
H10C	0.425993	0.067802	0.968533	0.061*	
C11	0.32012 (8)	0.23140 (7)	0.78995 (5)	0.02279 (14)	
C12	0.23371 (10)	0.30612 (9)	0.82268 (7)	0.0326 (2)	
H12A	0.257946	0.318850	0.879329	0.049*	
H12B	0.140923	0.286798	0.820185	0.049*	
H12C	0.245700	0.361630	0.789941	0.049*	
C13	0.51254 (8)	0.28766 (6)	0.45699 (5)	0.01942 (12)	
C14	0.60642 (10)	0.29793 (8)	0.38706 (6)	0.02799 (17)	
H14A	0.635766	0.361791	0.383460	0.042*	
H14B	0.561686	0.280639	0.336407	0.042*	
H14C	0.683247	0.258094	0.395553	0.042*	
C15	0.93368 (7)	0.44946 (5)	0.68253 (5)	0.01753 (11)	
C16	1.07409 (8)	0.53539 (6)	0.75814 (5)	0.02136 (13)	
C21	0.94674 (8)	0.44529 (5)	0.53444 (5)	0.01757 (11)	
C22	1.08346 (8)	0.44191 (6)	0.52310 (5)	0.02145 (13)	
H22	1.139890	0.424160	0.566583	0.026*	
C23	1.13650 (10)	0.46468 (7)	0.44773 (6)	0.02549 (15)	
H23	1.229554	0.462378	0.439753	0.031*	
C24	1.05447 (12)	0.49080 (6)	0.38397 (5)	0.02644 (17)	
H24	1.091369	0.507208	0.332812	0.032*	
C25	0.91818 (11)	0.49283 (7)	0.39531 (5)	0.02647 (16)	
H25	0.861821	0.509730	0.351523	0.032*	
C26	0.86378 (9)	0.47010 (6)	0.47083 (5)	0.02296 (13)	
H26	0.770643	0.471623	0.478597	0.028*	
S1	0.86470 (2)	0.40549 (2)	0.77293 (2)	0.01858 (4)	
O1	0.67241 (5)	0.32676 (4)	0.68992 (4)	0.01607 (9)	
O2	0.84565 (6)	0.21987 (4)	0.85986 (4)	0.01974 (10)	
O3	0.58385 (7)	0.14504 (4)	0.87307 (4)	0.02067 (11)	
O4	0.44272 (6)	0.26432 (4)	0.77268 (4)	0.01925 (9)	
O6	0.57635 (6)	0.29829 (5)	0.52853 (4)	0.02020 (10)	
O7	0.79450 (9)	0.29683 (8)	0.97457 (6)	0.0389 (2)	
O8	0.61654 (11)	−0.00264 (5)	0.83803 (6)	0.0384 (2)	
O9	0.28822 (9)	0.15313 (7)	0.77933 (7)	0.0389 (2)	
O10	0.39564 (7)	0.27080 (5)	0.45226 (5)	0.02582 (13)	
N1	0.88912 (7)	0.42008 (5)	0.61039 (4)	0.01991 (11)	
H01	0.8146 (19)	0.3876 (13)	0.6127 (12)	0.035 (4)*	
N2	1.02637 (8)	0.51190 (5)	0.68629 (5)	0.02161 (12)	
N3	1.12347 (9)	0.56121 (7)	0.81801 (6)	0.02844 (15)	

### 2',3',4',6'-Tetra-O-acetyl-β-D-galactopyranosyl N'-cyano-N-phenylcarbamimidothioate (5b) Atomic displacement parameters (Å^2^)

**Table:**

	*U*^11^	*U*^22^	*U*^33^	*U*^12^	*U*^13^	*U*^23^
C1	0.0132 (2)	0.0157 (2)	0.0173 (2)	−0.00148 (19)	0.00097 (19)	−0.00066 (19)
C2	0.0147 (2)	0.0155 (2)	0.0177 (2)	0.0007 (2)	0.00178 (19)	0.0007 (2)
C3	0.0173 (3)	0.0135 (2)	0.0198 (3)	0.0000 (2)	0.0037 (2)	0.0012 (2)
C4	0.0155 (2)	0.0138 (2)	0.0214 (3)	−0.0014 (2)	0.0026 (2)	−0.0011 (2)
C5	0.0148 (2)	0.0151 (2)	0.0192 (3)	−0.0019 (2)	0.0001 (2)	−0.0015 (2)
C6	0.0152 (2)	0.0221 (3)	0.0213 (3)	−0.0026 (2)	−0.0006 (2)	0.0002 (2)
C7	0.0204 (3)	0.0240 (3)	0.0204 (3)	−0.0040 (3)	−0.0015 (2)	0.0004 (2)
C8	0.0304 (4)	0.0276 (4)	0.0286 (4)	−0.0007 (3)	−0.0100 (3)	0.0048 (3)
C9	0.0278 (4)	0.0165 (3)	0.0319 (4)	−0.0022 (3)	0.0078 (3)	0.0049 (3)
C10	0.0522 (7)	0.0260 (4)	0.0445 (6)	0.0008 (4)	0.0246 (6)	0.0127 (4)
C11	0.0150 (3)	0.0298 (4)	0.0236 (3)	−0.0021 (3)	0.0024 (2)	0.0001 (3)
C12	0.0192 (3)	0.0429 (6)	0.0357 (5)	0.0061 (3)	0.0048 (3)	−0.0054 (4)
C13	0.0190 (3)	0.0181 (3)	0.0212 (3)	0.0001 (2)	−0.0035 (2)	−0.0003 (2)
C14	0.0245 (4)	0.0375 (5)	0.0220 (3)	0.0003 (3)	−0.0013 (3)	0.0008 (3)
C15	0.0161 (3)	0.0193 (3)	0.0171 (2)	−0.0043 (2)	0.0016 (2)	−0.0015 (2)
C16	0.0181 (3)	0.0236 (3)	0.0224 (3)	−0.0064 (3)	0.0009 (2)	−0.0028 (2)
C21	0.0181 (3)	0.0181 (3)	0.0165 (2)	−0.0025 (2)	0.0012 (2)	−0.0010 (2)
C22	0.0181 (3)	0.0260 (3)	0.0203 (3)	−0.0013 (3)	0.0024 (2)	−0.0002 (3)
C23	0.0242 (3)	0.0294 (4)	0.0229 (3)	−0.0062 (3)	0.0076 (3)	−0.0047 (3)
C24	0.0406 (5)	0.0205 (3)	0.0182 (3)	−0.0061 (3)	0.0063 (3)	−0.0017 (2)
C25	0.0373 (5)	0.0236 (3)	0.0185 (3)	0.0052 (3)	−0.0025 (3)	−0.0007 (3)
C26	0.0220 (3)	0.0269 (3)	0.0199 (3)	0.0034 (3)	−0.0022 (3)	−0.0032 (3)
S1	0.01917 (7)	0.02049 (8)	0.01609 (7)	−0.00696 (6)	0.00176 (6)	−0.00168 (6)
O1	0.01402 (18)	0.0147 (2)	0.0195 (2)	−0.00223 (16)	−0.00084 (16)	0.00018 (17)
O2	0.0194 (2)	0.0207 (2)	0.0192 (2)	0.00408 (19)	−0.00146 (18)	0.00047 (18)
O3	0.0236 (3)	0.0151 (2)	0.0233 (2)	−0.00012 (19)	0.0073 (2)	0.00318 (18)
O4	0.01443 (19)	0.0171 (2)	0.0262 (2)	−0.00007 (16)	0.00425 (19)	−0.00096 (19)
O6	0.0165 (2)	0.0249 (3)	0.0192 (2)	−0.0036 (2)	−0.00175 (17)	−0.00031 (19)
O7	0.0300 (4)	0.0570 (6)	0.0298 (4)	0.0084 (4)	−0.0044 (3)	−0.0183 (4)
O8	0.0552 (6)	0.0161 (3)	0.0440 (4)	−0.0031 (3)	0.0193 (4)	−0.0005 (3)
O9	0.0258 (3)	0.0351 (4)	0.0557 (5)	−0.0130 (3)	0.0126 (4)	−0.0078 (4)
O10	0.0199 (2)	0.0286 (3)	0.0290 (3)	−0.0043 (2)	−0.0072 (2)	0.0033 (2)
N1	0.0187 (2)	0.0248 (3)	0.0163 (2)	−0.0079 (2)	0.00164 (19)	−0.0019 (2)
N2	0.0215 (3)	0.0238 (3)	0.0195 (3)	−0.0098 (2)	0.0014 (2)	−0.0020 (2)
N3	0.0246 (3)	0.0331 (4)	0.0276 (3)	−0.0065 (3)	−0.0043 (3)	−0.0066 (3)

### 2',3',4',6'-Tetra-O-acetyl-β-D-galactopyranosyl N'-cyano-N-phenylcarbamimidothioate (5b) Geometric parameters (Å, º)

**Table:**

C1—O1	1.4164 (9)	C11—O9	1.1999 (13)
C1—C2	1.5366 (10)	C11—O4	1.3588 (10)
C1—S1	1.8117 (7)	C11—C12	1.4970 (15)
C1—H1	1.0000	C12—H12A	0.9800
C2—O2	1.4357 (10)	C12—H12B	0.9800
C2—C3	1.5203 (11)	C12—H12C	0.9800
C2—H2	1.0000	C13—O10	1.2092 (11)
C3—O3	1.4354 (10)	C13—O6	1.3492 (10)
C3—C4	1.5203 (11)	C13—C14	1.4972 (13)
C3—H3	1.0000	C14—H14A	0.9800
C4—O4	1.4406 (9)	C14—H14B	0.9800
C4—C5	1.5223 (10)	C14—H14C	0.9800
C4—H4	1.0000	C15—N2	1.3084 (10)
C5—O1	1.4445 (9)	C15—N1	1.3382 (10)
C5—C6	1.5087 (11)	C15—S1	1.7613 (8)
C5—H5	1.0000	C16—N3	1.1653 (12)
C6—O6	1.4419 (10)	C16—N2	1.3203 (11)
C6—H6A	0.9900	C21—C26	1.3877 (12)
C6—H6B	0.9900	C21—C22	1.3950 (12)
C7—O7	1.2057 (13)	C21—N1	1.4250 (10)
C7—O2	1.3581 (10)	C22—C23	1.3893 (12)
C7—C8	1.4912 (14)	C22—H22	0.9500
C8—H8A	0.9800	C23—C24	1.3890 (15)
C8—H8B	0.9800	C23—H23	0.9500
C8—H8C	0.9800	C24—C25	1.3901 (17)
C9—O8	1.2048 (13)	C24—H24	0.9500
C9—O3	1.3544 (11)	C25—C26	1.3969 (13)
C9—C10	1.5007 (15)	C25—H25	0.9500
C10—H10A	0.9800	C26—H26	0.9500
C10—H10B	0.9800	N1—H01	0.891 (19)
C10—H10C	0.9800		
			
O1—C1—C2	111.00 (6)	H10A—C10—H10C	109.5
O1—C1—S1	108.95 (5)	H10B—C10—H10C	109.5
C2—C1—S1	106.00 (5)	O9—C11—O4	123.46 (9)
O1—C1—H1	110.3	O9—C11—C12	126.11 (9)
C2—C1—H1	110.3	O4—C11—C12	110.43 (8)
S1—C1—H1	110.3	C11—C12—H12A	109.5
O2—C2—C3	107.54 (6)	C11—C12—H12B	109.5
O2—C2—C1	107.06 (6)	H12A—C12—H12B	109.5
C3—C2—C1	110.01 (6)	C11—C12—H12C	109.5
O2—C2—H2	110.7	H12A—C12—H12C	109.5
C3—C2—H2	110.7	H12B—C12—H12C	109.5
C1—C2—H2	110.7	O10—C13—O6	123.11 (8)
O3—C3—C4	110.46 (6)	O10—C13—C14	126.16 (8)
O3—C3—C2	106.51 (6)	O6—C13—C14	110.72 (7)
C4—C3—C2	112.30 (6)	C13—C14—H14A	109.5
O3—C3—H3	109.2	C13—C14—H14B	109.5
C4—C3—H3	109.2	H14A—C14—H14B	109.5
C2—C3—H3	109.2	C13—C14—H14C	109.5
O4—C4—C3	110.63 (6)	H14A—C14—H14C	109.5
O4—C4—C5	108.69 (6)	H14B—C14—H14C	109.5
C3—C4—C5	108.58 (6)	N2—C15—N1	120.39 (7)
O4—C4—H4	109.6	N2—C15—S1	119.82 (6)
C3—C4—H4	109.6	N1—C15—S1	119.77 (6)
C5—C4—H4	109.6	N3—C16—N2	174.12 (10)
O1—C5—C6	107.47 (6)	C26—C21—C22	120.46 (8)
O1—C5—C4	109.91 (6)	C26—C21—N1	118.67 (7)
C6—C5—C4	112.51 (6)	C22—C21—N1	120.83 (7)
O1—C5—H5	109.0	C23—C22—C21	119.52 (8)
C6—C5—H5	109.0	C23—C22—H22	120.2
C4—C5—H5	109.0	C21—C22—H22	120.2
O6—C6—C5	106.42 (6)	C24—C23—C22	120.49 (9)
O6—C6—H6A	110.4	C24—C23—H23	119.8
C5—C6—H6A	110.4	C22—C23—H23	119.8
O6—C6—H6B	110.4	C23—C24—C25	119.74 (8)
C5—C6—H6B	110.4	C23—C24—H24	120.1
H6A—C6—H6B	108.6	C25—C24—H24	120.1
O7—C7—O2	122.93 (9)	C24—C25—C26	120.25 (9)
O7—C7—C8	126.07 (9)	C24—C25—H25	119.9
O2—C7—C8	110.99 (8)	C26—C25—H25	119.9
C7—C8—H8A	109.5	C21—C26—C25	119.53 (9)
C7—C8—H8B	109.5	C21—C26—H26	120.2
H8A—C8—H8B	109.5	C25—C26—H26	120.2
C7—C8—H8C	109.5	C15—S1—C1	104.12 (3)
H8A—C8—H8C	109.5	C1—O1—C5	110.78 (6)
H8B—C8—H8C	109.5	C7—O2—C2	118.03 (7)
O8—C9—O3	123.51 (9)	C9—O3—C3	117.19 (7)
O8—C9—C10	126.72 (9)	C11—O4—C4	116.66 (7)
O3—C9—C10	109.78 (8)	C13—O6—C6	116.09 (6)
C9—C10—H10A	109.5	C15—N1—C21	123.71 (7)
C9—C10—H10B	109.5	C15—N1—H01	114.7 (12)
H10A—C10—H10B	109.5	C21—N1—H01	121.4 (12)
C9—C10—H10C	109.5	C15—N2—C16	118.99 (7)
			
O1—C1—C2—O2	170.57 (6)	O1—C1—S1—C15	−66.61 (5)
S1—C1—C2—O2	−71.27 (6)	C2—C1—S1—C15	173.87 (5)
O1—C1—C2—C3	54.00 (8)	C2—C1—O1—C5	−62.61 (7)
S1—C1—C2—C3	172.17 (5)	S1—C1—O1—C5	−178.96 (5)
O2—C2—C3—O3	72.99 (7)	C6—C5—O1—C1	−171.62 (6)
C1—C2—C3—O3	−170.74 (6)	C4—C5—O1—C1	65.64 (7)
O2—C2—C3—C4	−165.98 (6)	O7—C7—O2—C2	−3.38 (14)
C1—C2—C3—C4	−49.71 (8)	C8—C7—O2—C2	176.27 (7)
O3—C3—C4—O4	51.85 (8)	C3—C2—O2—C7	−115.48 (7)
C2—C3—C4—O4	−66.88 (8)	C1—C2—O2—C7	126.34 (7)
O3—C3—C4—C5	171.05 (6)	O8—C9—O3—C3	1.08 (16)
C2—C3—C4—C5	52.31 (8)	C10—C9—O3—C3	−178.97 (10)
O4—C4—C5—O1	61.46 (8)	C4—C3—O3—C9	99.81 (9)
C3—C4—C5—O1	−58.94 (8)	C2—C3—O3—C9	−137.99 (8)
O4—C4—C5—C6	−58.25 (8)	O9—C11—O4—C4	2.34 (14)
C3—C4—C5—C6	−178.66 (6)	C12—C11—O4—C4	−177.24 (8)
O1—C5—C6—O6	71.79 (7)	C3—C4—O4—C11	−108.49 (8)
C4—C5—C6—O6	−167.08 (6)	C5—C4—O4—C11	132.38 (7)
C26—C21—C22—C23	0.78 (13)	O10—C13—O6—C6	7.97 (12)
N1—C21—C22—C23	178.38 (8)	C14—C13—O6—C6	−171.01 (8)
C21—C22—C23—C24	0.09 (14)	C5—C6—O6—C13	151.83 (7)
C22—C23—C24—C25	−0.97 (14)	N2—C15—N1—C21	7.25 (13)
C23—C24—C25—C26	1.00 (15)	S1—C15—N1—C21	−174.06 (7)
C22—C21—C26—C25	−0.76 (13)	C26—C21—N1—C15	−135.44 (9)
N1—C21—C26—C25	−178.40 (8)	C22—C21—N1—C15	46.92 (12)
C24—C25—C26—C21	−0.14 (14)	N1—C15—N2—C16	−175.83 (9)
N2—C15—S1—C1	−164.31 (7)	S1—C15—N2—C16	5.47 (12)
N1—C15—S1—C1	16.99 (8)		

### 2',3',4',6'-Tetra-O-acetyl-β-D-galactopyranosyl N'-cyano-N-phenylcarbamimidothioate (5b) Hydrogen-bond geometry (Å, º)

**Table:**

*D*—H···*A*	*D*—H	H···*A*	*D*···*A*	*D*—H···*A*
N1—H01···O1	0.891 (19)	2.112 (19)	2.8910 (9)	145.5 (17)
C3—H3···N3^i^	1.00	2.54	3.4648 (12)	153
C6—H6*B*···O8^ii^	0.99	2.62	3.5823 (12)	164
C10—H10*B*···O6^iii^	0.98	2.59	3.5652 (14)	172
C10—H10*C*···O7^iv^	0.98	2.56	3.4276 (17)	148
C23—H23···N3^v^	0.95	2.51	3.2506 (13)	134
C25—H25···S1^vi^	0.95	2.90	3.7968 (10)	157
N1—H01···O10^vii^	0.891 (19)	2.675 (19)	2.9710 (11)	100.6 (13)

Symmetry codes: (i) −*x*+2, *y*−1/2, −*z*+3/2; (ii) −*x*+1, *y*+1/2, −*z*+3/2; (iii) −*x*+1, *y*−1/2, −*z*+3/2; (iv) *x*−1/2, −*y*+1/2, −*z*+2; (v) −*x*+5/2, −*y*+1, *z*−1/2; (vi) −*x*+3/2, −*y*+1, *z*−1/2; (vii) *x*+1/2, −*y*+1/2, −*z*+1.

### 2',3',4',6'-Tetra-O-acetyl-β-D-galactopyranosyl N'-cyano-N-methylcarbamimidothioate (5c) Crystal data

**Table:**

C_17_H_23_N_3_O_9_S	*D*_x_ = 1.373 Mg m^−^^3^
*M**_r_* = 445.44	Cu *K*α radiation, λ = 1.54184 Å
Orthorhombic, *P*2_1_2_1_2_1_	Cell parameters from 79622 reflections
*a* = 7.34295 (7) Å	θ = 3.8–80.0°
*b* = 13.92258 (13) Å	µ = 1.81 mm^−^^1^
*c* = 21.07464 (18) Å	*T* = 100 K
*V* = 2154.52 (3) Å^3^	Lath, colourless
*Z* = 4	0.18 × 0.08 × 0.06 mm
*F*(000) = 936	

### 2',3',4',6'-Tetra-O-acetyl-β-D-galactopyranosyl N'-cyano-N-methylcarbamimidothioate (5c) Data collection

**Table:**

XtaLAB Synergy diffractometer	4696 independent reflections
Radiation source: micro-focus sealed X-ray tube, PhotonJet (Cu) X-ray Source	4656 reflections with *I* > 2σ(*I*)
Mirror monochromator	*R*_int_ = 0.047
Detector resolution: 10.0000 pixels mm^-1^	θ_max_ = 80.4°, θ_min_ = 3.8°
ω scans	*h* = −9→8
Absorption correction: multi-scan (CrysAlisPro; Rigaku OD, 2023)	*k* = −17→17
*T*_min_ = 0.694, *T*_max_ = 1.000	*l* = −26→26
101235 measured reflections	

### 2',3',4',6'-Tetra-O-acetyl-β-D-galactopyranosyl N'-cyano-N-methylcarbamimidothioate (5c) Refinement

**Table:**

Refinement on *F*^2^	Hydrogen site location: mixed
Least-squares matrix: full	H atoms treated by a mixture of independent and constrained refinement
*R*[*F*^2^ > 2σ(*F*^2^)] = 0.024	*w* = 1/[σ^2^(*F*_o_^2^) + (0.0391*P*)^2^ + 0.5241*P*] where *P* = (*F*_o_^2^ + 2*F*_c_^2^)/3
*wR*(*F*^2^) = 0.065	(Δ/σ)_max_ = 0.001
*S* = 1.05	Δρ_max_ = 0.21 e Å^−^^3^
4696 reflections	Δρ_min_ = −0.24 e Å^−^^3^
280 parameters	Absolute structure: Flack *x* determined using 1977 quotients [(*I*^+^)-(*I*^-^)]/[(*I*^+^)+(*I*^-^)] (Parsons *et al.*, 2013)
0 restraints	Absolute structure parameter: 0.000 (4)
Primary atom site location: dual	

### 2',3',4',6'-Tetra-O-acetyl-β-D-galactopyranosyl N'-cyano-N-methylcarbamimidothioate (5c) Special details

**Table:**

Geometry. The symmetry employed for this shelxl refinement is uniquely defined by the following loop, which should always be used as a source of symmetry information in preference to the above space-group names. They are only intended as comments.

### 2',3',4',6'-Tetra-O-acetyl-β-D-galactopyranosyl N'-cyano-N-methylcarbamimidothioate (5c) Fractional atomic coordinates and isotropic or equivalent isotropic displacement parameters (Å^2^)

**Table:**

	*x*	*y*	*z*	*U*_iso_*/*U*_eq_	
C1	0.7245 (2)	0.66817 (11)	0.24727 (7)	0.0175 (3)	
H1	0.647646	0.622077	0.222619	0.021*	
C2	0.7436 (2)	0.63486 (11)	0.31640 (7)	0.0175 (3)	
H2	0.821482	0.680873	0.340715	0.021*	
C3	0.5550 (2)	0.62966 (11)	0.34555 (7)	0.0186 (3)	
H3	0.481679	0.578900	0.323780	0.022*	
C4	0.4581 (2)	0.72607 (11)	0.34056 (7)	0.0185 (3)	
H4	0.329134	0.719336	0.355137	0.022*	
C5	0.4620 (2)	0.75995 (12)	0.27171 (7)	0.0190 (3)	
H5	0.386177	0.715479	0.245419	0.023*	
C6	0.3941 (2)	0.86165 (13)	0.26249 (8)	0.0220 (3)	
H6A	0.465331	0.906758	0.289018	0.026*	
H6B	0.406753	0.881043	0.217511	0.026*	
C7	0.9950 (2)	0.53207 (12)	0.34050 (8)	0.0212 (3)	
C8	1.0445 (3)	0.42928 (13)	0.34965 (10)	0.0297 (4)	
H8A	0.985654	0.404839	0.388139	0.045*	
H8B	1.176944	0.423482	0.353899	0.045*	
H8C	1.003474	0.391885	0.312942	0.045*	
C9	0.5360 (3)	0.51546 (12)	0.43004 (8)	0.0230 (3)	
C10	0.5724 (4)	0.50524 (16)	0.49966 (9)	0.0377 (5)	
H10A	0.699096	0.485345	0.506176	0.057*	
H10B	0.490580	0.456708	0.517579	0.057*	
H10C	0.551481	0.566968	0.520780	0.057*	
C11	0.4671 (3)	0.82059 (13)	0.43472 (8)	0.0261 (4)	
C12	0.5824 (3)	0.88888 (17)	0.47181 (9)	0.0372 (5)	
H12A	0.698702	0.858196	0.482204	0.056*	
H12B	0.519163	0.906535	0.511071	0.056*	
H12C	0.604794	0.946716	0.446483	0.056*	
C13	0.1541 (2)	0.92475 (13)	0.32696 (8)	0.0221 (3)	
C14	−0.0443 (3)	0.91212 (15)	0.34173 (10)	0.0327 (4)	
H14A	−0.109624	0.893846	0.303029	0.049*	
H14B	−0.093902	0.972604	0.358067	0.049*	
H14C	−0.058888	0.861664	0.373752	0.049*	
C15	0.9103 (2)	0.69674 (12)	0.13212 (8)	0.0181 (3)	
C16	1.2166 (2)	0.68567 (14)	0.11683 (8)	0.0249 (4)	
C17	0.7119 (3)	0.73704 (15)	0.04334 (8)	0.0285 (4)	
H17A	0.770265	0.689313	0.015862	0.043*	
H17B	0.762210	0.800769	0.034218	0.043*	
H17C	0.580422	0.737392	0.035412	0.043*	
S1	0.95046 (5)	0.67585 (3)	0.21342 (2)	0.01798 (9)	
O1	0.64503 (16)	0.76112 (8)	0.24695 (5)	0.0186 (2)	
O2	0.81975 (17)	0.54006 (8)	0.31925 (6)	0.0189 (2)	
O3	0.57416 (18)	0.60665 (8)	0.41145 (5)	0.0217 (2)	
O4	0.55196 (18)	0.79518 (8)	0.37979 (5)	0.0202 (2)	
O6	0.20487 (16)	0.86283 (9)	0.28113 (6)	0.0221 (2)	
O7	1.09046 (18)	0.60030 (10)	0.35057 (7)	0.0316 (3)	
O8	0.4844 (2)	0.45319 (9)	0.39517 (7)	0.0297 (3)	
O9	0.3195 (2)	0.79158 (11)	0.44988 (7)	0.0380 (4)	
O10	0.25533 (19)	0.98057 (9)	0.35237 (6)	0.0279 (3)	
N1	0.7460 (2)	0.71274 (11)	0.10956 (7)	0.0217 (3)	
H01	0.652 (4)	0.7055 (19)	0.1332 (13)	0.040 (7)*	
N2	1.0520 (2)	0.70085 (11)	0.09343 (6)	0.0213 (3)	
N3	1.3664 (2)	0.67346 (17)	0.13200 (8)	0.0384 (4)	

### 2',3',4',6'-Tetra-O-acetyl-β-D-galactopyranosyl N'-cyano-N-methylcarbamimidothioate (5c) Atomic displacement parameters (Å^2^)

**Table:**

	*U*^11^	*U*^22^	*U*^33^	*U*^12^	*U*^13^	*U*^23^
C1	0.0156 (7)	0.0199 (7)	0.0169 (7)	0.0000 (6)	0.0008 (6)	0.0010 (6)
C2	0.0188 (8)	0.0164 (7)	0.0173 (7)	−0.0002 (6)	0.0004 (6)	0.0014 (6)
C3	0.0205 (8)	0.0199 (7)	0.0153 (7)	−0.0012 (7)	0.0012 (6)	0.0018 (5)
C4	0.0177 (7)	0.0198 (7)	0.0180 (7)	−0.0008 (6)	0.0012 (6)	−0.0002 (6)
C5	0.0163 (7)	0.0222 (7)	0.0186 (7)	−0.0004 (7)	0.0015 (6)	0.0003 (6)
C6	0.0180 (8)	0.0252 (8)	0.0228 (8)	0.0036 (6)	0.0030 (6)	0.0029 (6)
C7	0.0198 (8)	0.0235 (8)	0.0202 (7)	−0.0002 (6)	−0.0033 (6)	0.0010 (6)
C8	0.0289 (9)	0.0234 (8)	0.0369 (9)	0.0040 (8)	−0.0100 (8)	0.0013 (7)
C9	0.0197 (8)	0.0231 (8)	0.0261 (8)	0.0008 (7)	0.0039 (7)	0.0060 (6)
C10	0.0536 (14)	0.0347 (10)	0.0249 (9)	−0.0017 (10)	0.0019 (9)	0.0107 (7)
C11	0.0326 (9)	0.0261 (8)	0.0194 (7)	0.0043 (8)	0.0044 (7)	−0.0010 (6)
C12	0.0434 (12)	0.0422 (11)	0.0260 (9)	0.0012 (10)	−0.0007 (8)	−0.0113 (8)
C13	0.0228 (8)	0.0221 (8)	0.0214 (7)	0.0035 (7)	−0.0002 (7)	0.0024 (6)
C14	0.0231 (9)	0.0388 (10)	0.0361 (10)	−0.0005 (8)	0.0074 (8)	−0.0075 (8)
C15	0.0176 (8)	0.0190 (7)	0.0176 (7)	−0.0005 (6)	0.0003 (6)	−0.0004 (6)
C16	0.0194 (8)	0.0387 (10)	0.0166 (7)	−0.0024 (7)	0.0024 (6)	−0.0017 (7)
C17	0.0240 (9)	0.0420 (10)	0.0196 (8)	0.0059 (8)	−0.0023 (7)	0.0020 (8)
S1	0.01381 (17)	0.02384 (18)	0.01629 (17)	−0.00034 (15)	−0.00027 (14)	0.00170 (13)
O1	0.0168 (5)	0.0193 (5)	0.0197 (5)	0.0016 (4)	0.0026 (4)	0.0023 (4)
O2	0.0177 (6)	0.0172 (5)	0.0219 (6)	0.0004 (4)	−0.0022 (4)	0.0009 (4)
O3	0.0269 (6)	0.0221 (5)	0.0162 (5)	−0.0004 (5)	0.0013 (5)	0.0028 (4)
O4	0.0213 (5)	0.0217 (5)	0.0175 (5)	0.0006 (5)	0.0018 (5)	−0.0017 (4)
O6	0.0164 (5)	0.0252 (6)	0.0246 (6)	0.0023 (5)	0.0002 (5)	−0.0026 (5)
O7	0.0221 (7)	0.0256 (6)	0.0472 (8)	−0.0029 (5)	−0.0103 (6)	−0.0004 (6)
O8	0.0332 (7)	0.0232 (6)	0.0327 (7)	−0.0053 (5)	−0.0017 (6)	0.0047 (5)
O9	0.0399 (8)	0.0419 (8)	0.0323 (7)	−0.0035 (7)	0.0172 (7)	−0.0059 (6)
O10	0.0266 (7)	0.0270 (6)	0.0300 (7)	−0.0003 (5)	−0.0013 (5)	−0.0039 (5)
N1	0.0153 (7)	0.0322 (7)	0.0175 (7)	0.0020 (6)	0.0006 (6)	0.0043 (6)
N2	0.0157 (7)	0.0297 (7)	0.0184 (6)	−0.0008 (6)	−0.0006 (5)	−0.0011 (5)
N3	0.0182 (7)	0.0710 (13)	0.0258 (7)	0.0004 (9)	0.0014 (6)	−0.0035 (9)

### 2',3',4',6'-Tetra-O-acetyl-β-D-galactopyranosyl N'-cyano-N-methylcarbamimidothioate (5c) Geometric parameters (Å, º)

**Table:**

C1—O1	1.4195 (19)	C9—C10	1.498 (3)
C1—C2	1.535 (2)	C10—H10A	0.9800
C1—S1	1.8093 (16)	C10—H10B	0.9800
C1—H1	1.0000	C10—H10C	0.9800
C2—O2	1.4346 (19)	C11—O9	1.200 (3)
C2—C3	1.517 (2)	C11—O4	1.361 (2)
C2—H2	1.0000	C11—C12	1.493 (3)
C3—O3	1.4321 (18)	C12—H12A	0.9800
C3—C4	1.523 (2)	C12—H12B	0.9800
C3—H3	1.0000	C12—H12C	0.9800
C4—O4	1.4438 (19)	C13—O10	1.201 (2)
C4—C5	1.526 (2)	C13—O6	1.347 (2)
C4—H4	1.0000	C13—C14	1.500 (3)
C5—O1	1.4421 (19)	C14—H14A	0.9800
C5—C6	1.513 (2)	C14—H14B	0.9800
C5—H5	1.0000	C14—H14C	0.9800
C6—O6	1.444 (2)	C15—N1	1.316 (2)
C6—H6A	0.9900	C15—N2	1.323 (2)
C6—H6B	0.9900	C15—S1	1.7628 (17)
C7—O7	1.199 (2)	C16—N3	1.158 (3)
C7—O2	1.367 (2)	C16—N2	1.322 (2)
C7—C8	1.489 (2)	C17—N1	1.458 (2)
C8—H8A	0.9800	C17—H17A	0.9800
C8—H8B	0.9800	C17—H17B	0.9800
C8—H8C	0.9800	C17—H17C	0.9800
C9—O8	1.198 (2)	N1—H01	0.86 (3)
C9—O3	1.358 (2)		
			
O1—C1—C2	108.51 (12)	O8—C9—C10	126.05 (16)
O1—C1—S1	108.74 (10)	O3—C9—C10	109.56 (15)
C2—C1—S1	107.94 (11)	C9—C10—H10A	109.5
O1—C1—H1	110.5	C9—C10—H10B	109.5
C2—C1—H1	110.5	H10A—C10—H10B	109.5
S1—C1—H1	110.5	C9—C10—H10C	109.5
O2—C2—C3	107.15 (13)	H10A—C10—H10C	109.5
O2—C2—C1	110.69 (13)	H10B—C10—H10C	109.5
C3—C2—C1	108.38 (13)	O9—C11—O4	123.51 (18)
O2—C2—H2	110.2	O9—C11—C12	125.92 (17)
C3—C2—H2	110.2	O4—C11—C12	110.57 (17)
C1—C2—H2	110.2	C11—C12—H12A	109.5
O3—C3—C2	108.29 (13)	C11—C12—H12B	109.5
O3—C3—C4	108.06 (13)	H12A—C12—H12B	109.5
C2—C3—C4	110.90 (13)	C11—C12—H12C	109.5
O3—C3—H3	109.9	H12A—C12—H12C	109.5
C2—C3—H3	109.9	H12B—C12—H12C	109.5
C4—C3—H3	109.9	O10—C13—O6	124.22 (17)
O4—C4—C3	108.95 (13)	O10—C13—C14	125.75 (18)
O4—C4—C5	109.25 (12)	O6—C13—C14	110.02 (16)
C3—C4—C5	109.23 (13)	C13—C14—H14A	109.5
O4—C4—H4	109.8	C13—C14—H14B	109.5
C3—C4—H4	109.8	H14A—C14—H14B	109.5
C5—C4—H4	109.8	C13—C14—H14C	109.5
O1—C5—C6	104.46 (13)	H14A—C14—H14C	109.5
O1—C5—C4	111.40 (13)	H14B—C14—H14C	109.5
C6—C5—C4	113.90 (13)	N1—C15—N2	119.42 (15)
O1—C5—H5	109.0	N1—C15—S1	122.16 (13)
C6—C5—H5	109.0	N2—C15—S1	118.34 (13)
C4—C5—H5	109.0	N3—C16—N2	174.04 (19)
O6—C6—C5	107.00 (14)	N1—C17—H17A	109.5
O6—C6—H6A	110.3	N1—C17—H17B	109.5
C5—C6—H6A	110.3	H17A—C17—H17B	109.5
O6—C6—H6B	110.3	N1—C17—H17C	109.5
C5—C6—H6B	110.3	H17A—C17—H17C	109.5
H6A—C6—H6B	108.6	H17B—C17—H17C	109.5
O7—C7—O2	122.93 (16)	C15—S1—C1	103.86 (8)
O7—C7—C8	126.55 (17)	C1—O1—C5	111.79 (12)
O2—C7—C8	110.51 (14)	C7—O2—C2	117.09 (13)
C7—C8—H8A	109.5	C9—O3—C3	117.96 (13)
C7—C8—H8B	109.5	C11—O4—C4	116.22 (14)
H8A—C8—H8B	109.5	C13—O6—C6	117.93 (14)
C7—C8—H8C	109.5	C15—N1—C17	122.85 (15)
H8A—C8—H8C	109.5	C15—N1—H01	120.6 (18)
H8B—C8—H8C	109.5	C17—N1—H01	116.4 (18)
O8—C9—O3	124.38 (16)	C16—N2—C15	118.82 (14)
			
O1—C1—C2—O2	178.70 (12)	S1—C1—O1—C5	178.36 (10)
S1—C1—C2—O2	−63.63 (14)	C6—C5—O1—C1	−175.22 (13)
O1—C1—C2—C3	61.46 (16)	C4—C5—O1—C1	61.38 (16)
S1—C1—C2—C3	179.13 (11)	O7—C7—O2—C2	−7.4 (2)
O2—C2—C3—O3	65.42 (15)	C8—C7—O2—C2	171.72 (14)
C1—C2—C3—O3	−175.09 (12)	C3—C2—O2—C7	−133.78 (14)
O2—C2—C3—C4	−176.17 (12)	C1—C2—O2—C7	108.23 (15)
C1—C2—C3—C4	−56.69 (16)	O8—C9—O3—C3	−1.3 (3)
O3—C3—C4—O4	51.99 (17)	C10—C9—O3—C3	177.46 (16)
C2—C3—C4—O4	−66.56 (16)	C2—C3—O3—C9	−103.98 (17)
O3—C3—C4—C5	171.26 (13)	C4—C3—O3—C9	135.83 (15)
C2—C3—C4—C5	52.72 (17)	O9—C11—O4—C4	−2.9 (3)
O4—C4—C5—O1	65.43 (17)	C12—C11—O4—C4	177.55 (15)
C3—C4—C5—O1	−53.66 (17)	C3—C4—O4—C11	−106.48 (16)
O4—C4—C5—C6	−52.41 (19)	C5—C4—O4—C11	134.26 (15)
C3—C4—C5—C6	−171.50 (14)	O10—C13—O6—C6	1.7 (3)
O1—C5—C6—O6	174.83 (12)	C14—C13—O6—C6	−177.37 (15)
C4—C5—C6—O6	−63.41 (17)	C5—C6—O6—C13	123.04 (16)
N1—C15—S1—C1	6.76 (16)	N2—C15—N1—C17	−1.3 (3)
N2—C15—S1—C1	−176.51 (13)	S1—C15—N1—C17	175.39 (14)
O1—C1—S1—C15	−70.91 (12)	N1—C15—N2—C16	179.27 (17)
C2—C1—S1—C15	171.56 (11)	S1—C15—N2—C16	2.4 (2)
C2—C1—O1—C5	−64.48 (15)		

### 2',3',4',6'-Tetra-O-acetyl-β-D-galactopyranosyl N'-cyano-N-methylcarbamimidothioate (5c) Hydrogen-bond geometry (Å, º)

**Table:**

*D*—H···*A*	*D*—H	H···*A*	*D*···*A*	*D*—H···*A*
N1—H01···O1	0.86 (3)	2.52 (3)	3.0637 (19)	122 (2)
N1—H01···N3^i^	0.86 (3)	2.14 (3)	2.880 (2)	144 (2)
C4—H4···O7^i^	1.00	2.41	3.225 (2)	138
C5—H5···N3^i^	1.00	2.47	3.258 (2)	136
C1—H1···O10^ii^	1.00	2.62	3.355 (2)	130
C8—H8*C*···O6^ii^	0.98	2.54	3.436 (2)	153
C8—H8*B*···O8^iii^	0.98	2.45	3.386 (3)	159

Symmetry codes: (i) *x*−1, *y*, *z*; (ii) −*x*+1, *y*−1/2, −*z*+1/2; (iii) *x*+1, *y*, *z*.

### 2',3',4',6'-Tetra-O-acetyl-β-D-galactopyranosyl N'-cyano-N-p-tolylcarbamimidothioate (5d) Crystal data

**Table:**

C_23_H_27_N_3_O_9_S	*D*_x_ = 1.371 Mg m^−^^3^
*M**_r_* = 521.53	Mo *K*α radiation, λ = 0.71073 Å
Orthorhombic, *P*2_1_2_1_2_1_	Cell parameters from 63582 reflections
*a* = 7.42044 (17) Å	θ = 2.3–38.4°
*b* = 14.9634 (4) Å	µ = 0.18 mm^−^^1^
*c* = 22.7580 (5) Å	*T* = 100 K
*V* = 2526.94 (11) Å^3^	Prism, colourless
*Z* = 4	0.15 × 0.05 × 0.04 mm
*F*(000) = 1096	

### 2',3',4',6'-Tetra-O-acetyl-β-D-galactopyranosyl N'-cyano-N-p-tolylcarbamimidothioate (5d) Data collection

**Table:**

XtaLAB Synergy diffractometer	12241 independent reflections
Radiation source: micro-focus sealed X-ray tube, PhotonJet (Mo) X-ray Source	11008 reflections with *I* > 2σ(*I*)
Mirror monochromator	*R*_int_ = 0.085
Detector resolution: 10.0000 pixels mm^-1^	θ_max_ = 36.3°, θ_min_ = 2.3°
ω scans	*h* = −12→12
Absorption correction: multi-scan (CrysAlisPro; Rigaku OD, 2023)	*k* = −24→24
*T*_min_ = 0.881, *T*_max_ = 1.000	*l* = −37→37
146926 measured reflections	

### 2',3',4',6'-Tetra-O-acetyl-β-D-galactopyranosyl N'-cyano-N-p-tolylcarbamimidothioate (5d) Refinement

**Table:**

Refinement on *F*^2^	Hydrogen site location: mixed
Least-squares matrix: full	H atoms treated by a mixture of independent and constrained refinement
*R*[*F*^2^ > 2σ(*F*^2^)] = 0.036	*w* = 1/[σ^2^(*F*_o_^2^) + (0.0589*P*)^2^ + 0.1217*P*] where *P* = (*F*_o_^2^ + 2*F*_c_^2^)/3
*wR*(*F*^2^) = 0.094	(Δ/σ)_max_ = 0.001
*S* = 1.04	Δρ_max_ = 0.45 e Å^−^^3^
12241 reflections	Δρ_min_ = −0.21 e Å^−^^3^
334 parameters	Absolute structure: Flack *x* determined using 4554 quotients [(*I*^+^)-(*I*^-^)]/[(*I*^+^)+(*I*^-^)] (Parsons *et al.*, 2013)
0 restraints	Absolute structure parameter: 0.005 (18)
Primary atom site location: dual	

### 2',3',4',6'-Tetra-O-acetyl-β-D-galactopyranosyl N'-cyano-N-p-tolylcarbamimidothioate (5d) Special details

**Table:**

Geometry. The symmetry employed for this shelxl refinement is uniquely defined by the following loop, which should always be used as a source of symmetry information in preference to the above space-group names. They are only intended as comments.

### 2',3',4',6'-Tetra-O-acetyl-β-D-galactopyranosyl N'-cyano-N-p-tolylcarbamimidothioate (5d) Fractional atomic coordinates and isotropic or equivalent isotropic displacement parameters (Å^2^)

**Table:**

	*x*	*y*	*z*	*U*_iso_*/*U*_eq_	
C1	0.37733 (14)	0.22950 (8)	0.76889 (5)	0.01328 (17)	
H1	0.305633	0.178158	0.753244	0.016*	
C2	0.41195 (15)	0.21795 (8)	0.83531 (5)	0.01282 (17)	
H2	0.493689	0.266177	0.849975	0.015*	
C3	0.23143 (15)	0.22161 (8)	0.86735 (5)	0.01317 (18)	
H3	0.156478	0.168366	0.856960	0.016*	
C4	0.13113 (15)	0.30760 (8)	0.85209 (5)	0.01346 (18)	
H4	0.008781	0.307097	0.870508	0.016*	
C5	0.11397 (15)	0.31525 (8)	0.78577 (5)	0.01416 (18)	
H5	0.036989	0.265376	0.770766	0.017*	
C6	0.03836 (17)	0.40360 (9)	0.76458 (6)	0.0189 (2)	
H6A	0.114753	0.453639	0.778354	0.023*	
H6B	0.034313	0.404954	0.721112	0.023*	
C7	0.67169 (18)	0.12911 (9)	0.85531 (6)	0.0180 (2)	
C8	0.7319 (2)	0.03566 (11)	0.86740 (9)	0.0312 (3)	
H8A	0.712097	0.021643	0.908968	0.047*	
H8B	0.860457	0.029971	0.858225	0.047*	
H8C	0.662784	−0.005941	0.842957	0.047*	
C9	0.15614 (17)	0.17555 (9)	0.96477 (5)	0.0169 (2)	
C10	0.18293 (19)	0.20008 (11)	1.02776 (6)	0.0230 (3)	
H10A	0.309037	0.216918	1.034168	0.034*	
H10B	0.152722	0.148837	1.052697	0.034*	
H10C	0.104629	0.250583	1.037745	0.034*	
C11	0.18220 (19)	0.41801 (9)	0.92642 (6)	0.0196 (2)	
C12	0.3059 (2)	0.49047 (10)	0.94601 (7)	0.0233 (3)	
H12A	0.393763	0.466129	0.973886	0.035*	
H12B	0.235980	0.537817	0.965146	0.035*	
H12C	0.369274	0.515173	0.911908	0.035*	
C13	−0.23441 (18)	0.48185 (9)	0.76781 (7)	0.0205 (2)	
C14	−0.4220 (2)	0.48191 (11)	0.79243 (9)	0.0291 (3)	
H14A	−0.488383	0.430260	0.777176	0.044*	
H14B	−0.483761	0.537043	0.780799	0.044*	
H14C	−0.416446	0.478483	0.835394	0.044*	
C15	0.56188 (15)	0.20458 (8)	0.65971 (5)	0.01335 (18)	
C16	0.86849 (16)	0.19725 (10)	0.65229 (5)	0.0178 (2)	
C21	0.36887 (15)	0.16707 (8)	0.57647 (5)	0.01467 (19)	
C22	0.46087 (17)	0.20570 (9)	0.52958 (5)	0.0180 (2)	
H22	0.544346	0.252735	0.536297	0.022*	
C23	0.42959 (18)	0.17486 (10)	0.47281 (6)	0.0199 (2)	
H23	0.494175	0.200657	0.441010	0.024*	
C24	0.30585 (18)	0.10711 (9)	0.46155 (6)	0.0187 (2)	
C25	0.21309 (19)	0.06976 (9)	0.50905 (6)	0.0189 (2)	
H25	0.127528	0.023683	0.502248	0.023*	
C26	0.24442 (17)	0.09919 (9)	0.56619 (5)	0.0172 (2)	
H26	0.181007	0.072996	0.598105	0.021*	
C27	0.2693 (2)	0.07582 (11)	0.39971 (6)	0.0270 (3)	
H27A	0.262273	0.010429	0.399076	0.040*	
H27B	0.367052	0.095674	0.373854	0.040*	
H27C	0.154905	0.101027	0.386004	0.040*	
S1	0.59555 (4)	0.23470 (2)	0.73362 (2)	0.01528 (6)	
O1	0.28752 (12)	0.31129 (6)	0.75797 (4)	0.01475 (15)	
O2	0.48923 (13)	0.13189 (6)	0.84644 (4)	0.01579 (16)	
O3	0.26992 (13)	0.22212 (7)	0.92918 (4)	0.01660 (16)	
O4	0.23358 (13)	0.38279 (6)	0.87373 (4)	0.01566 (15)	
O6	−0.14014 (13)	0.41145 (7)	0.78837 (5)	0.02144 (19)	
O7	0.76619 (13)	0.19386 (7)	0.85272 (5)	0.02141 (18)	
O8	0.04661 (18)	0.12360 (9)	0.94637 (5)	0.0309 (3)	
O9	0.05355 (18)	0.39191 (9)	0.95381 (6)	0.0328 (3)	
O10	−0.17235 (17)	0.53562 (8)	0.73421 (6)	0.0301 (2)	
N1	0.39889 (14)	0.19629 (8)	0.63591 (4)	0.01517 (17)	
H01	0.305 (3)	0.1970 (15)	0.6584 (10)	0.029 (6)*	
N2	0.70777 (13)	0.19005 (8)	0.62801 (4)	0.01625 (18)	
N3	1.01735 (16)	0.20315 (10)	0.66804 (5)	0.0245 (2)	

### 2',3',4',6'-Tetra-O-acetyl-β-D-galactopyranosyl N'-cyano-N-p-tolylcarbamimidothioate (5d) Atomic displacement parameters (Å^2^)

**Table:**

	*U*^11^	*U*^22^	*U*^33^	*U*^12^	*U*^13^	*U*^23^
C1	0.0109 (4)	0.0161 (4)	0.0128 (4)	−0.0002 (4)	0.0009 (3)	−0.0013 (4)
C2	0.0108 (4)	0.0145 (4)	0.0132 (4)	0.0007 (4)	0.0006 (3)	−0.0012 (3)
C3	0.0123 (4)	0.0162 (4)	0.0110 (4)	−0.0013 (4)	0.0008 (3)	−0.0013 (3)
C4	0.0103 (4)	0.0152 (4)	0.0149 (4)	−0.0012 (4)	0.0003 (3)	−0.0023 (4)
C5	0.0102 (4)	0.0172 (5)	0.0152 (4)	0.0003 (4)	−0.0001 (3)	−0.0001 (4)
C6	0.0130 (4)	0.0207 (5)	0.0230 (5)	0.0028 (4)	0.0003 (4)	0.0035 (4)
C7	0.0148 (5)	0.0223 (5)	0.0169 (5)	0.0034 (4)	−0.0025 (4)	−0.0026 (4)
C8	0.0245 (7)	0.0224 (6)	0.0467 (9)	0.0083 (5)	−0.0109 (7)	−0.0037 (6)
C9	0.0153 (5)	0.0220 (5)	0.0133 (4)	0.0021 (4)	0.0016 (4)	0.0027 (4)
C10	0.0184 (5)	0.0375 (7)	0.0130 (5)	0.0025 (5)	0.0009 (4)	0.0004 (5)
C11	0.0195 (5)	0.0181 (5)	0.0211 (5)	−0.0003 (4)	0.0019 (4)	−0.0057 (4)
C12	0.0276 (7)	0.0195 (5)	0.0228 (6)	−0.0037 (5)	−0.0036 (5)	−0.0041 (5)
C13	0.0165 (5)	0.0168 (5)	0.0282 (6)	0.0029 (4)	−0.0050 (5)	−0.0048 (5)
C14	0.0158 (6)	0.0227 (6)	0.0487 (9)	0.0024 (5)	0.0002 (6)	−0.0061 (6)
C15	0.0099 (4)	0.0174 (5)	0.0128 (4)	−0.0002 (4)	0.0001 (3)	−0.0003 (3)
C16	0.0121 (4)	0.0289 (6)	0.0124 (4)	0.0008 (4)	0.0012 (3)	−0.0034 (4)
C21	0.0106 (4)	0.0196 (5)	0.0138 (4)	0.0006 (4)	−0.0013 (3)	−0.0003 (4)
C22	0.0145 (5)	0.0241 (6)	0.0156 (5)	−0.0041 (4)	−0.0028 (4)	0.0031 (4)
C23	0.0173 (5)	0.0288 (6)	0.0137 (5)	−0.0015 (5)	−0.0017 (4)	0.0032 (4)
C24	0.0171 (5)	0.0236 (6)	0.0155 (5)	0.0031 (4)	−0.0026 (4)	−0.0020 (4)
C25	0.0171 (5)	0.0208 (5)	0.0188 (5)	−0.0012 (4)	−0.0020 (4)	−0.0027 (4)
C26	0.0132 (5)	0.0210 (5)	0.0174 (5)	−0.0013 (4)	−0.0004 (4)	−0.0004 (4)
C27	0.0308 (7)	0.0326 (7)	0.0174 (5)	−0.0001 (6)	−0.0041 (5)	−0.0041 (5)
S1	0.01011 (10)	0.02345 (13)	0.01230 (10)	−0.00192 (10)	0.00084 (8)	−0.00367 (9)
O1	0.0119 (3)	0.0170 (4)	0.0154 (4)	0.0007 (3)	0.0011 (3)	0.0014 (3)
O2	0.0130 (3)	0.0162 (4)	0.0182 (4)	0.0012 (3)	−0.0017 (3)	−0.0005 (3)
O3	0.0152 (4)	0.0242 (4)	0.0103 (3)	−0.0030 (3)	0.0004 (3)	−0.0004 (3)
O4	0.0142 (4)	0.0162 (4)	0.0167 (4)	−0.0021 (3)	0.0004 (3)	−0.0034 (3)
O6	0.0133 (4)	0.0204 (4)	0.0305 (5)	0.0035 (3)	0.0006 (4)	0.0043 (4)
O7	0.0153 (4)	0.0260 (5)	0.0230 (4)	−0.0010 (4)	−0.0028 (3)	−0.0015 (4)
O8	0.0343 (6)	0.0393 (6)	0.0191 (4)	−0.0193 (5)	0.0004 (4)	0.0046 (4)
O9	0.0288 (6)	0.0349 (6)	0.0347 (6)	−0.0088 (5)	0.0156 (5)	−0.0162 (5)
O10	0.0291 (5)	0.0246 (5)	0.0366 (6)	0.0077 (4)	0.0004 (5)	0.0068 (5)
N1	0.0094 (4)	0.0226 (5)	0.0135 (4)	−0.0004 (4)	−0.0010 (3)	−0.0016 (3)
N2	0.0094 (4)	0.0266 (5)	0.0128 (4)	0.0009 (4)	−0.0001 (3)	−0.0033 (4)
N3	0.0118 (4)	0.0434 (7)	0.0184 (5)	0.0005 (5)	0.0003 (3)	−0.0045 (5)

### 2',3',4',6'-Tetra-O-acetyl-β-D-galactopyranosyl N'-cyano-N-p-tolylcarbamimidothioate (5d) Geometric parameters (Å, º)

**Table:**

C1—O1	1.4155 (15)	C11—O4	1.3642 (16)
C1—C2	1.5431 (16)	C11—C12	1.489 (2)
C1—S1	1.8090 (11)	C12—H12A	0.9800
C1—H1	1.0000	C12—H12B	0.9800
C2—O2	1.4322 (15)	C12—H12C	0.9800
C2—C3	1.5261 (16)	C13—O10	1.2016 (19)
C2—H2	1.0000	C13—O6	1.3484 (16)
C3—O3	1.4359 (14)	C13—C14	1.501 (2)
C3—C4	1.5265 (17)	C14—H14A	0.9800
C3—H3	1.0000	C14—H14B	0.9800
C4—O4	1.4443 (14)	C14—H14C	0.9800
C4—C5	1.5190 (16)	C15—N2	1.3190 (15)
C4—H4	1.0000	C15—N1	1.3311 (15)
C5—O1	1.4361 (14)	C15—S1	1.7593 (12)
C5—C6	1.5150 (17)	C16—N3	1.1647 (16)
C5—H5	1.0000	C16—N2	1.3188 (16)
C6—O6	1.4356 (16)	C21—C22	1.3924 (17)
C6—H6A	0.9900	C21—C26	1.3927 (17)
C6—H6B	0.9900	C21—N1	1.4388 (16)
C7—O7	1.1975 (18)	C22—C23	1.3913 (18)
C7—O2	1.3695 (16)	C22—H22	0.9500
C7—C8	1.494 (2)	C23—C24	1.3917 (19)
C8—H8A	0.9800	C23—H23	0.9500
C8—H8B	0.9800	C24—C25	1.3980 (19)
C8—H8C	0.9800	C24—C27	1.5078 (19)
C9—O8	1.2000 (18)	C25—C26	1.3925 (18)
C9—O3	1.3617 (15)	C25—H25	0.9500
C9—C10	1.4930 (18)	C26—H26	0.9500
C10—H10A	0.9800	C27—H27A	0.9800
C10—H10B	0.9800	C27—H27B	0.9800
C10—H10C	0.9800	C27—H27C	0.9800
C11—O9	1.2052 (18)	N1—H01	0.87 (2)
			
O1—C1—C2	110.32 (9)	O9—C11—C12	124.70 (13)
O1—C1—S1	107.84 (8)	O4—C11—C12	111.86 (12)
C2—C1—S1	106.88 (7)	C11—C12—H12A	109.5
O1—C1—H1	110.6	C11—C12—H12B	109.5
C2—C1—H1	110.6	H12A—C12—H12B	109.5
S1—C1—H1	110.6	C11—C12—H12C	109.5
O2—C2—C3	107.41 (9)	H12A—C12—H12C	109.5
O2—C2—C1	109.91 (9)	H12B—C12—H12C	109.5
C3—C2—C1	108.53 (9)	O10—C13—O6	123.03 (13)
O2—C2—H2	110.3	O10—C13—C14	126.35 (13)
C3—C2—H2	110.3	O6—C13—C14	110.62 (13)
C1—C2—H2	110.3	C13—C14—H14A	109.5
O3—C3—C2	107.09 (9)	C13—C14—H14B	109.5
O3—C3—C4	108.39 (9)	H14A—C14—H14B	109.5
C2—C3—C4	110.46 (9)	C13—C14—H14C	109.5
O3—C3—H3	110.3	H14A—C14—H14C	109.5
C2—C3—H3	110.3	H14B—C14—H14C	109.5
C4—C3—H3	110.3	N2—C15—N1	120.52 (10)
O4—C4—C5	108.92 (10)	N2—C15—S1	116.65 (9)
O4—C4—C3	108.81 (9)	N1—C15—S1	122.82 (9)
C5—C4—C3	109.29 (9)	N3—C16—N2	173.13 (13)
O4—C4—H4	109.9	C22—C21—C26	119.94 (11)
C5—C4—H4	109.9	C22—C21—N1	121.23 (11)
C3—C4—H4	109.9	C26—C21—N1	118.83 (11)
O1—C5—C6	103.17 (10)	C23—C22—C21	119.49 (12)
O1—C5—C4	111.07 (9)	C23—C22—H22	120.3
C6—C5—C4	114.40 (10)	C21—C22—H22	120.3
O1—C5—H5	109.3	C22—C23—C24	121.51 (12)
C6—C5—H5	109.3	C22—C23—H23	119.2
C4—C5—H5	109.3	C24—C23—H23	119.2
O6—C6—C5	107.04 (10)	C23—C24—C25	118.27 (11)
O6—C6—H6A	110.3	C23—C24—C27	121.11 (13)
C5—C6—H6A	110.3	C25—C24—C27	120.61 (13)
O6—C6—H6B	110.3	C26—C25—C24	120.90 (12)
C5—C6—H6B	110.3	C26—C25—H25	119.6
H6A—C6—H6B	108.6	C24—C25—H25	119.6
O7—C7—O2	123.16 (13)	C25—C26—C21	119.88 (12)
O7—C7—C8	126.25 (13)	C25—C26—H26	120.1
O2—C7—C8	110.58 (12)	C21—C26—H26	120.1
C7—C8—H8A	109.5	C24—C27—H27A	109.5
C7—C8—H8B	109.5	C24—C27—H27B	109.5
H8A—C8—H8B	109.5	H27A—C27—H27B	109.5
C7—C8—H8C	109.5	C24—C27—H27C	109.5
H8A—C8—H8C	109.5	H27A—C27—H27C	109.5
H8B—C8—H8C	109.5	H27B—C27—H27C	109.5
O8—C9—O3	122.95 (12)	C15—S1—C1	106.62 (5)
O8—C9—C10	125.76 (13)	C1—O1—C5	112.36 (9)
O3—C9—C10	111.26 (12)	C7—O2—C2	116.69 (10)
C9—C10—H10A	109.5	C9—O3—C3	117.18 (10)
C9—C10—H10B	109.5	C11—O4—C4	116.97 (10)
H10A—C10—H10B	109.5	C13—O6—C6	114.31 (11)
C9—C10—H10C	109.5	C15—N1—C21	123.47 (10)
H10A—C10—H10C	109.5	C15—N1—H01	119.4 (15)
H10B—C10—H10C	109.5	C21—N1—H01	115.8 (16)
O9—C11—O4	123.42 (13)	C16—N2—C15	119.97 (10)
			
O1—C1—C2—O2	175.24 (9)	N1—C15—S1—C1	10.44 (12)
S1—C1—C2—O2	−67.78 (10)	O1—C1—S1—C15	−83.66 (8)
O1—C1—C2—C3	58.03 (12)	C2—C1—S1—C15	157.73 (8)
S1—C1—C2—C3	175.02 (7)	C2—C1—O1—C5	−61.80 (12)
O2—C2—C3—O3	68.38 (11)	S1—C1—O1—C5	−178.19 (7)
C1—C2—C3—O3	−172.83 (9)	C6—C5—O1—C1	−175.80 (10)
O2—C2—C3—C4	−173.79 (9)	C4—C5—O1—C1	61.19 (12)
C1—C2—C3—C4	−55.00 (12)	O7—C7—O2—C2	−1.84 (18)
O3—C3—C4—O4	52.67 (12)	C8—C7—O2—C2	178.96 (12)
C2—C3—C4—O4	−64.35 (11)	C3—C2—O2—C7	−141.16 (10)
O3—C3—C4—C5	171.50 (9)	C1—C2—O2—C7	100.94 (12)
C2—C3—C4—C5	54.47 (12)	O8—C9—O3—C3	12.60 (19)
O4—C4—C5—O1	62.60 (12)	C10—C9—O3—C3	−165.63 (11)
C3—C4—C5—O1	−56.15 (12)	C2—C3—O3—C9	−142.63 (10)
O4—C4—C5—C6	−53.69 (12)	C4—C3—O3—C9	98.19 (12)
C3—C4—C5—C6	−172.44 (10)	O9—C11—O4—C4	−3.0 (2)
O1—C5—C6—O6	177.48 (10)	C12—C11—O4—C4	175.80 (11)
C4—C5—C6—O6	−61.75 (13)	C5—C4—O4—C11	143.06 (11)
C26—C21—C22—C23	−1.10 (19)	C3—C4—O4—C11	−97.88 (12)
N1—C21—C22—C23	179.26 (12)	O10—C13—O6—C6	−2.8 (2)
C21—C22—C23—C24	1.1 (2)	C14—C13—O6—C6	176.91 (12)
C22—C23—C24—C25	−0.4 (2)	C5—C6—O6—C13	−172.45 (11)
C22—C23—C24—C27	178.37 (13)	N2—C15—N1—C21	3.38 (19)
C23—C24—C25—C26	−0.4 (2)	S1—C15—N1—C21	−176.54 (9)
C27—C24—C25—C26	−179.13 (13)	C22—C21—N1—C15	−50.03 (18)
C24—C25—C26—C21	0.4 (2)	C26—C21—N1—C15	130.33 (13)
C22—C21—C26—C25	0.37 (19)	N1—C15—N2—C16	−179.05 (13)
N1—C21—C26—C25	−179.98 (12)	S1—C15—N2—C16	0.87 (17)
N2—C15—S1—C1	−169.48 (10)		

### 2',3',4',6'-Tetra-O-acetyl-β-D-galactopyranosyl N'-cyano-N-p-tolylcarbamimidothioate (5d) Hydrogen-bond geometry (Å, º)

**Table:**

*D*—H···*A*	*D*—H	H···*A*	*D*···*A*	*D*—H···*A*
N1—H01···N3^i^	0.87 (2)	2.15 (2)	2.9259 (16)	150 (2)
C14—H14*A*···O1^i^	0.98	2.47	3.4320 (18)	165
C4—H4···O7^i^	1.00	2.51	3.1985 (15)	126
C5—H5···N3^i^	1.00	2.52	3.2414 (17)	129
C1—H1···O10^ii^	1.00	2.37	3.2765 (16)	151
C25—H25···O9^ii^	0.95	2.59	3.4223 (19)	147
C10—H10*A*···O9^iii^	0.98	2.45	3.1038 (19)	124
C10—H10*C*···O3^iv^	0.98	2.63	3.4217 (17)	138
C12—H12*A*···O8^iii^	0.98	2.53	3.4790 (19)	164

Symmetry codes: (i) *x*−1, *y*, *z*; (ii) −*x*, *y*−1/2, −*z*+3/2; (iii) *x*+1/2, −*y*+1/2, −*z*+2; (iv) *x*−1/2, −*y*+1/2, −*z*+2.

### 2',3',4',6'-Tetra-O-acetyl-β-D-galactopyranosyl N'-cyano-N-p-tolylcarbamimidothioate (5dCu) Crystal data

**Table:**

C_23_H_27_N_3_O_9_S	*D*_x_ = 1.371 Mg m^−^^3^
*M**_r_* = 521.53	Cu *K*α radiation, λ = 1.54184 Å
Orthorhombic, *P*2_1_2_1_2_1_	Cell parameters from 90210 reflections
*a* = 7.41967 (5) Å	θ = 3.5–80.0°
*b* = 14.96519 (12) Å	µ = 1.63 mm^−^^1^
*c* = 22.75622 (16) Å	*T* = 100 K
*V* = 2526.78 (3) Å^3^	Prism, colourless
*Z* = 4	0.15 × 0.05 × 0.04 mm
*F*(000) = 1096	

### 2',3',4',6'-Tetra-O-acetyl-β-D-galactopyranosyl N'-cyano-N-p-tolylcarbamimidothioate (5dCu) Data collection

**Table:**

XtaLAB Synergy diffractometer	5508 independent reflections
Radiation source: micro-focus sealed X-ray tube, PhotonJet (Cu) X-ray Source	5458 reflections with *I* > 2σ(*I*)
Mirror monochromator	*R*_int_ = 0.033
Detector resolution: 10.0000 pixels mm^-1^	θ_max_ = 80.6°, θ_min_ = 3.5°
ω scans	*h* = −9→9
Absorption correction: multi-scan (CrysAlisPro; Rigaku OD, 2023)	*k* = −18→19
*T*_min_ = 0.781, *T*_max_ = 1.000	*l* = −28→28
122360 measured reflections	

### 2',3',4',6'-Tetra-O-acetyl-β-D-galactopyranosyl N'-cyano-N-p-tolylcarbamimidothioate (5dCu) Refinement

**Table:**

Refinement on *F*^2^	Hydrogen site location: mixed
Least-squares matrix: full	H atoms treated by a mixture of independent and constrained refinement
*R*[*F*^2^ > 2σ(*F*^2^)] = 0.023	*w* = 1/[σ^2^(*F*_o_^2^) + (0.0299*P*)^2^ + 0.7621*P*] where *P* = (*F*_o_^2^ + 2*F*_c_^2^)/3
*wR*(*F*^2^) = 0.059	(Δ/σ)_max_ = 0.002
*S* = 1.04	Δρ_max_ = 0.22 e Å^−^^3^
5508 reflections	Δρ_min_ = −0.18 e Å^−^^3^
334 parameters	Absolute structure: Flack *x* determined using 2334 quotients [(*I*^+^)-(*I*^-^)]/[(*I*^+^)+(*I*^-^)] (Parsons *et al.*, 2013)
0 restraints	Absolute structure parameter: −0.004 (2)
Primary atom site location: dual	

### 2',3',4',6'-Tetra-O-acetyl-β-D-galactopyranosyl N'-cyano-N-p-tolylcarbamimidothioate (5dCu) Special details

**Table:**

Geometry. All esds (except the esd in the dihedral angle between two l.s. planes) are estimated using the full covariance matrix. The cell esds are taken into account individually in the estimation of esds in distances, angles and torsion angles; correlations between esds in cell parameters are only used when they are defined by crystal symmetry. An approximate (isotropic) treatment of cell esds is used for estimating esds involving l.s. planes.

### 2',3',4',6'-Tetra-O-acetyl-β-D-galactopyranosyl N'-cyano-N-p-tolylcarbamimidothioate (5dCu) Fractional atomic coordinates and isotropic or equivalent isotropic displacement parameters (Å^2^)

**Table:**

	*x*	*y*	*z*	*U*_iso_*/*U*_eq_	
C1	0.37718 (19)	0.22936 (11)	0.76887 (7)	0.0156 (3)	
H1	0.305463	0.178029	0.753220	0.019*	
C2	0.4117 (2)	0.21801 (10)	0.83539 (6)	0.0152 (3)	
H2	0.493541	0.266236	0.850011	0.018*	
C3	0.2315 (2)	0.22186 (10)	0.86723 (6)	0.0155 (3)	
H3	0.156595	0.168623	0.856807	0.019*	
C4	0.1309 (2)	0.30765 (11)	0.85211 (7)	0.0161 (3)	
H4	0.008592	0.307059	0.870571	0.019*	
C5	0.1138 (2)	0.31552 (11)	0.78568 (7)	0.0171 (3)	
H5	0.036670	0.265711	0.770640	0.020*	
C6	0.0386 (2)	0.40350 (11)	0.76458 (8)	0.0216 (3)	
H6A	0.115147	0.453474	0.778348	0.026*	
H6B	0.034706	0.404828	0.721102	0.026*	
C7	0.6716 (2)	0.12912 (12)	0.85530 (7)	0.0200 (3)	
C8	0.7317 (3)	0.03574 (13)	0.86753 (10)	0.0334 (4)	
H8A	0.706042	0.020803	0.908620	0.050*	
H8B	0.861516	0.030769	0.860319	0.050*	
H8C	0.666942	−0.005650	0.841684	0.050*	
C9	0.1561 (2)	0.17554 (11)	0.96477 (7)	0.0187 (3)	
C10	0.1830 (2)	0.20009 (13)	1.02771 (7)	0.0250 (4)	
H10A	0.304948	0.223927	1.033097	0.038*	
H10B	0.167381	0.146942	1.052348	0.038*	
H10C	0.094493	0.245531	1.039034	0.038*	
C11	0.1819 (2)	0.41827 (11)	0.92639 (8)	0.0216 (3)	
C12	0.3057 (3)	0.49040 (12)	0.94585 (8)	0.0252 (4)	
H12A	0.395173	0.465700	0.973058	0.038*	
H12B	0.236442	0.537242	0.965739	0.038*	
H12C	0.367223	0.515843	0.911615	0.038*	
C13	−0.2346 (2)	0.48187 (11)	0.76775 (8)	0.0227 (3)	
C14	−0.4219 (3)	0.48203 (13)	0.79244 (10)	0.0317 (4)	
H14A	−0.489544	0.431364	0.776357	0.048*	
H14B	−0.482462	0.537927	0.781767	0.048*	
H14C	−0.416279	0.476901	0.835333	0.048*	
C15	0.5619 (2)	0.20451 (10)	0.65955 (7)	0.0158 (3)	
C16	0.8689 (2)	0.19724 (12)	0.65229 (7)	0.0199 (3)	
C21	0.3689 (2)	0.16701 (11)	0.57631 (7)	0.0167 (3)	
C22	0.4604 (2)	0.20567 (12)	0.52972 (7)	0.0208 (3)	
H22	0.543454	0.252860	0.536484	0.025*	
C23	0.4295 (2)	0.17480 (12)	0.47293 (7)	0.0222 (3)	
H23	0.494441	0.200514	0.441181	0.027*	
C24	0.3057 (2)	0.10719 (11)	0.46157 (7)	0.0213 (3)	
C25	0.2134 (2)	0.06982 (12)	0.50901 (7)	0.0212 (3)	
H25	0.128093	0.023630	0.502222	0.025*	
C26	0.2446 (2)	0.09925 (11)	0.56614 (7)	0.0194 (3)	
H26	0.181204	0.073110	0.598069	0.023*	
C27	0.2696 (3)	0.07577 (14)	0.39966 (8)	0.0294 (4)	
H27A	0.259841	0.010468	0.399217	0.044*	
H27B	0.368852	0.094415	0.374026	0.044*	
H27C	0.156622	0.102027	0.385524	0.044*	
S1	0.59563 (5)	0.23477 (3)	0.73360 (2)	0.01751 (9)	
O1	0.28768 (14)	0.31131 (7)	0.75791 (5)	0.0168 (2)	
O2	0.48915 (15)	0.13193 (8)	0.84639 (5)	0.0178 (2)	
O3	0.26977 (15)	0.22223 (8)	0.92923 (5)	0.0185 (2)	
O4	0.23369 (15)	0.38296 (7)	0.87375 (5)	0.0173 (2)	
O6	−0.14007 (16)	0.41167 (8)	0.78826 (6)	0.0237 (3)	
O7	0.76611 (16)	0.19387 (9)	0.85272 (5)	0.0235 (3)	
O8	0.0469 (2)	0.12371 (10)	0.94639 (6)	0.0324 (3)	
O9	0.0531 (2)	0.39229 (10)	0.95357 (7)	0.0355 (3)	
O10	−0.17244 (19)	0.53560 (9)	0.73424 (7)	0.0325 (3)	
N1	0.39897 (18)	0.19627 (9)	0.63588 (6)	0.0174 (3)	
H01	0.302 (3)	0.2023 (15)	0.6577 (10)	0.028 (6)*	
N2	0.70775 (18)	0.19009 (10)	0.62802 (6)	0.0185 (3)	
N3	1.0174 (2)	0.20304 (12)	0.66802 (6)	0.0267 (3)	

### 2',3',4',6'-Tetra-O-acetyl-β-D-galactopyranosyl N'-cyano-N-p-tolylcarbamimidothioate (5dCu) Atomic displacement parameters (Å^2^)

**Table:**

	*U*^11^	*U*^22^	*U*^33^	*U*^12^	*U*^13^	*U*^23^
C1	0.0132 (7)	0.0195 (7)	0.0143 (6)	0.0002 (6)	0.0015 (5)	−0.0016 (6)
C2	0.0140 (7)	0.0164 (7)	0.0153 (7)	0.0020 (6)	0.0000 (6)	−0.0018 (5)
C3	0.0149 (7)	0.0199 (7)	0.0119 (6)	−0.0012 (6)	0.0003 (5)	−0.0012 (5)
C4	0.0125 (7)	0.0180 (7)	0.0178 (7)	−0.0019 (6)	0.0009 (5)	−0.0029 (6)
C5	0.0124 (7)	0.0211 (7)	0.0177 (7)	0.0003 (6)	0.0002 (6)	−0.0003 (6)
C6	0.0156 (7)	0.0249 (8)	0.0242 (8)	0.0016 (6)	0.0002 (6)	0.0027 (7)
C7	0.0166 (7)	0.0266 (9)	0.0167 (7)	0.0031 (7)	−0.0026 (6)	−0.0040 (6)
C8	0.0270 (9)	0.0255 (9)	0.0476 (11)	0.0081 (7)	−0.0101 (9)	−0.0044 (8)
C9	0.0157 (7)	0.0229 (8)	0.0176 (7)	0.0033 (6)	0.0018 (6)	0.0026 (6)
C10	0.0215 (8)	0.0375 (9)	0.0161 (7)	0.0034 (7)	0.0011 (6)	0.0003 (7)
C11	0.0224 (8)	0.0205 (8)	0.0219 (8)	0.0011 (7)	0.0015 (7)	−0.0047 (6)
C12	0.0290 (9)	0.0224 (8)	0.0243 (8)	−0.0034 (7)	−0.0028 (7)	−0.0041 (7)
C13	0.0202 (8)	0.0193 (7)	0.0287 (8)	0.0027 (6)	−0.0057 (7)	−0.0050 (7)
C14	0.0193 (9)	0.0263 (9)	0.0494 (11)	0.0034 (7)	−0.0007 (8)	−0.0064 (8)
C15	0.0142 (7)	0.0178 (7)	0.0155 (7)	0.0002 (6)	−0.0001 (6)	0.0005 (6)
C16	0.0166 (8)	0.0304 (8)	0.0127 (7)	0.0013 (7)	0.0030 (6)	−0.0040 (6)
C21	0.0127 (7)	0.0212 (8)	0.0161 (7)	0.0028 (6)	−0.0020 (6)	−0.0005 (6)
C22	0.0162 (7)	0.0257 (8)	0.0206 (8)	−0.0033 (6)	−0.0034 (6)	0.0023 (6)
C23	0.0186 (8)	0.0312 (9)	0.0167 (7)	−0.0003 (7)	−0.0013 (6)	0.0047 (6)
C24	0.0192 (8)	0.0255 (8)	0.0193 (8)	0.0046 (7)	−0.0028 (6)	−0.0015 (6)
C25	0.0180 (8)	0.0229 (8)	0.0228 (8)	0.0000 (7)	−0.0027 (6)	−0.0021 (6)
C26	0.0149 (7)	0.0236 (8)	0.0197 (7)	0.0000 (6)	0.0003 (6)	0.0007 (6)
C27	0.0328 (10)	0.0346 (10)	0.0210 (8)	0.0002 (8)	−0.0038 (8)	−0.0037 (7)
S1	0.01212 (16)	0.02639 (18)	0.01402 (15)	−0.00190 (14)	0.00099 (13)	−0.00383 (14)
O1	0.0138 (5)	0.0192 (5)	0.0174 (5)	0.0005 (4)	0.0012 (4)	0.0011 (4)
O2	0.0156 (5)	0.0184 (6)	0.0194 (5)	0.0016 (4)	−0.0014 (4)	−0.0005 (4)
O3	0.0168 (5)	0.0263 (6)	0.0123 (5)	−0.0028 (5)	0.0005 (4)	−0.0010 (4)
O4	0.0157 (5)	0.0182 (5)	0.0181 (5)	−0.0022 (4)	0.0004 (4)	−0.0032 (4)
O6	0.0149 (6)	0.0233 (6)	0.0329 (6)	0.0027 (5)	0.0007 (5)	0.0042 (5)
O7	0.0167 (5)	0.0289 (6)	0.0248 (6)	−0.0009 (5)	−0.0029 (5)	−0.0015 (5)
O8	0.0355 (8)	0.0406 (8)	0.0210 (6)	−0.0190 (6)	−0.0005 (5)	0.0048 (5)
O9	0.0311 (7)	0.0386 (8)	0.0367 (7)	−0.0096 (6)	0.0157 (6)	−0.0168 (6)
O10	0.0309 (7)	0.0272 (6)	0.0394 (7)	0.0075 (5)	0.0010 (6)	0.0071 (6)
N1	0.0119 (6)	0.0248 (7)	0.0156 (6)	0.0003 (5)	0.0006 (5)	−0.0016 (5)
N2	0.0119 (6)	0.0283 (7)	0.0154 (6)	0.0005 (5)	−0.0009 (5)	−0.0028 (5)
N3	0.0148 (7)	0.0465 (9)	0.0187 (7)	0.0009 (7)	0.0006 (5)	−0.0054 (6)

### 2',3',4',6'-Tetra-O-acetyl-β-D-galactopyranosyl N'-cyano-N-p-tolylcarbamimidothioate (5dCu) Geometric parameters (Å, º)

**Table:**

C1—O1	1.4168 (19)	C11—O4	1.364 (2)
C1—C2	1.545 (2)	C11—C12	1.485 (2)
C1—S1	1.8105 (15)	C12—H12A	0.9800
C1—H1	1.0000	C12—H12B	0.9800
C2—O2	1.4325 (18)	C12—H12C	0.9800
C2—C3	1.522 (2)	C13—O10	1.200 (2)
C2—H2	1.0000	C13—O6	1.347 (2)
C3—O3	1.4391 (17)	C13—C14	1.499 (3)
C3—C4	1.524 (2)	C14—H14A	0.9800
C3—H3	1.0000	C14—H14B	0.9800
C4—O4	1.4472 (18)	C14—H14C	0.9800
C4—C5	1.522 (2)	C15—N2	1.316 (2)
C4—H4	1.0000	C15—N1	1.329 (2)
C5—O1	1.4382 (18)	C15—S1	1.7628 (16)
C5—C6	1.508 (2)	C16—N3	1.162 (2)
C5—H5	1.0000	C16—N2	1.322 (2)
C6—O6	1.436 (2)	C21—C22	1.385 (2)
C6—H6A	0.9900	C21—C26	1.390 (2)
C6—H6B	0.9900	C21—N1	1.442 (2)
C7—O7	1.198 (2)	C22—C23	1.391 (2)
C7—O2	1.370 (2)	C22—H22	0.9500
C7—C8	1.493 (2)	C23—C24	1.390 (2)
C8—H8A	0.9800	C23—H23	0.9500
C8—H8B	0.9800	C24—C25	1.396 (2)
C8—H8C	0.9800	C24—C27	1.509 (2)
C9—O8	1.197 (2)	C25—C26	1.392 (2)
C9—O3	1.362 (2)	C25—H25	0.9500
C9—C10	1.492 (2)	C26—H26	0.9500
C10—H10A	0.9800	C27—H27A	0.9800
C10—H10B	0.9800	C27—H27B	0.9800
C10—H10C	0.9800	C27—H27C	0.9800
C11—O9	1.203 (2)	N1—H01	0.88 (2)
			
O1—C1—C2	110.21 (12)	O9—C11—C12	124.96 (16)
O1—C1—S1	107.63 (10)	O4—C11—C12	111.67 (15)
C2—C1—S1	106.90 (10)	C11—C12—H12A	109.5
O1—C1—H1	110.7	C11—C12—H12B	109.5
C2—C1—H1	110.7	H12A—C12—H12B	109.5
S1—C1—H1	110.7	C11—C12—H12C	109.5
O2—C2—C3	107.65 (12)	H12A—C12—H12C	109.5
O2—C2—C1	109.67 (12)	H12B—C12—H12C	109.5
C3—C2—C1	108.45 (12)	O10—C13—O6	122.86 (16)
O2—C2—H2	110.3	O10—C13—C14	126.39 (17)
C3—C2—H2	110.3	O6—C13—C14	110.75 (15)
C1—C2—H2	110.3	C13—C14—H14A	109.5
O3—C3—C2	107.07 (12)	C13—C14—H14B	109.5
O3—C3—C4	108.34 (12)	H14A—C14—H14B	109.5
C2—C3—C4	110.77 (12)	C13—C14—H14C	109.5
O3—C3—H3	110.2	H14A—C14—H14C	109.5
C2—C3—H3	110.2	H14B—C14—H14C	109.5
C4—C3—H3	110.2	N2—C15—N1	120.77 (14)
O4—C4—C5	108.77 (13)	N2—C15—S1	116.54 (12)
O4—C4—C3	108.72 (12)	N1—C15—S1	122.69 (12)
C5—C4—C3	109.29 (12)	N3—C16—N2	173.22 (16)
O4—C4—H4	110.0	C22—C21—C26	120.15 (15)
C5—C4—H4	110.0	C22—C21—N1	121.13 (14)
C3—C4—H4	110.0	C26—C21—N1	118.72 (14)
O1—C5—C6	103.30 (12)	C21—C22—C23	119.44 (15)
O1—C5—C4	110.99 (12)	C21—C22—H22	120.3
C6—C5—C4	114.51 (14)	C23—C22—H22	120.3
O1—C5—H5	109.3	C24—C23—C22	121.54 (15)
C6—C5—H5	109.3	C24—C23—H23	119.2
C4—C5—H5	109.3	C22—C23—H23	119.2
O6—C6—C5	107.22 (13)	C23—C24—C25	118.15 (15)
O6—C6—H6A	110.3	C23—C24—C27	121.17 (16)
C5—C6—H6A	110.3	C25—C24—C27	120.67 (16)
O6—C6—H6B	110.3	C26—C25—C24	120.94 (16)
C5—C6—H6B	110.3	C26—C25—H25	119.5
H6A—C6—H6B	108.5	C24—C25—H25	119.5
O7—C7—O2	123.14 (16)	C21—C26—C25	119.78 (15)
O7—C7—C8	126.27 (16)	C21—C26—H26	120.1
O2—C7—C8	110.58 (15)	C25—C26—H26	120.1
C7—C8—H8A	109.5	C24—C27—H27A	109.5
C7—C8—H8B	109.5	C24—C27—H27B	109.5
H8A—C8—H8B	109.5	H27A—C27—H27B	109.5
C7—C8—H8C	109.5	C24—C27—H27C	109.5
H8A—C8—H8C	109.5	H27A—C27—H27C	109.5
H8B—C8—H8C	109.5	H27B—C27—H27C	109.5
O8—C9—O3	122.97 (15)	C15—S1—C1	106.58 (7)
O8—C9—C10	125.85 (16)	C1—O1—C5	112.40 (11)
O3—C9—C10	111.15 (14)	C7—O2—C2	116.73 (13)
C9—C10—H10A	109.5	C9—O3—C3	117.27 (12)
C9—C10—H10B	109.5	C11—O4—C4	116.87 (12)
H10A—C10—H10B	109.5	C13—O6—C6	114.65 (14)
C9—C10—H10C	109.5	C15—N1—C21	123.34 (13)
H10A—C10—H10C	109.5	C15—N1—H01	120.3 (15)
H10B—C10—H10C	109.5	C21—N1—H01	116.0 (15)
O9—C11—O4	123.36 (16)	C15—N2—C16	120.18 (13)
			
O1—C1—C2—O2	175.42 (11)	N1—C15—S1—C1	10.56 (16)
S1—C1—C2—O2	−67.89 (14)	O1—C1—S1—C15	−83.79 (11)
O1—C1—C2—C3	58.12 (16)	C2—C1—S1—C15	157.82 (10)
S1—C1—C2—C3	174.81 (10)	C2—C1—O1—C5	−61.91 (15)
O2—C2—C3—O3	68.36 (15)	S1—C1—O1—C5	−178.15 (9)
C1—C2—C3—O3	−173.05 (12)	C6—C5—O1—C1	−175.75 (13)
O2—C2—C3—C4	−173.70 (12)	C4—C5—O1—C1	61.07 (16)
C1—C2—C3—C4	−55.11 (16)	O7—C7—O2—C2	−2.0 (2)
O3—C3—C4—O4	52.97 (15)	C8—C7—O2—C2	178.69 (14)
C2—C3—C4—O4	−64.20 (15)	C3—C2—O2—C7	−141.14 (13)
O3—C3—C4—C5	171.55 (12)	C1—C2—O2—C7	101.05 (15)
C2—C3—C4—C5	54.39 (16)	O8—C9—O3—C3	12.5 (2)
O4—C4—C5—O1	62.77 (16)	C10—C9—O3—C3	−165.64 (13)
C3—C4—C5—O1	−55.79 (16)	C2—C3—O3—C9	−142.40 (13)
O4—C4—C5—C6	−53.70 (17)	C4—C3—O3—C9	98.09 (15)
C3—C4—C5—C6	−172.26 (13)	O9—C11—O4—C4	−3.1 (2)
O1—C5—C6—O6	177.48 (12)	C12—C11—O4—C4	175.72 (14)
C4—C5—C6—O6	−61.70 (17)	C5—C4—O4—C11	142.86 (14)
C26—C21—C22—C23	−1.3 (2)	C3—C4—O4—C11	−98.22 (15)
N1—C21—C22—C23	179.15 (15)	O10—C13—O6—C6	−3.0 (2)
C21—C22—C23—C24	1.4 (3)	C14—C13—O6—C6	176.98 (14)
C22—C23—C24—C25	−0.7 (3)	C5—C6—O6—C13	−172.35 (14)
C22—C23—C24—C27	178.40 (17)	N2—C15—N1—C21	3.4 (2)
C23—C24—C25—C26	−0.1 (3)	S1—C15—N1—C21	−176.61 (12)
C27—C24—C25—C26	−179.24 (16)	C22—C21—N1—C15	−50.1 (2)
C22—C21—C26—C25	0.5 (2)	C26—C21—N1—C15	130.35 (17)
N1—C21—C26—C25	−179.94 (15)	N1—C15—N2—C16	−179.01 (17)
C24—C25—C26—C21	0.2 (3)	S1—C15—N2—C16	1.0 (2)
N2—C15—S1—C1	−169.42 (13)		

### 2',3',4',6'-Tetra-O-acetyl-β-D-galactopyranosyl N'-cyano-N-p-tolylcarbamimidothioate (5dCu) Hydrogen-bond geometry (Å, º)

**Table:**

*D*—H···*A*	*D*—H	H···*A*	*D*···*A*	*D*—H···*A*
N1—H01···N3^i^	0.88 (2)	2.13 (2)	2.926 (2)	151 (2)
C14—H14*A*···O1^i^	0.98	2.48	3.433 (2)	165
C4—H4···O7^i^	1.00	2.50	3.1976 (19)	126
C5—H5···N3^i^	1.00	2.52	3.243 (2)	129
C1—H1···O10^ii^	1.00	2.37	3.274 (2)	151
C25—H25···O9^ii^	0.95	2.59	3.420 (2)	147
C10—H10*A*···O9^iii^	0.98	2.55	3.103 (2)	116
C10—H10*C*···O3^iv^	0.98	2.56	3.423 (2)	147
C12—H12*A*···O8^iii^	0.98	2.53	3.483 (2)	163

Symmetry codes: (i) *x*−1, *y*, *z*; (ii) −*x*, *y*−1/2, −*z*+3/2; (iii) *x*+1/2, −*y*+1/2, −*z*+2; (iv) *x*−1/2, −*y*+1/2, −*z*+2.
